# Regiodivergence in the Cycloadditions between a Cyclic Nitrone and Carbonyl-Type Dipolarophiles

**DOI:** 10.1021/acs.joc.5c00727

**Published:** 2025-07-29

**Authors:** Alberto Esteban, Carlos T. Nieto, Narciso M. Garrido, Francisca Sanz, David Díez

**Affiliations:** † Organic Chemistry Department, Chemical Sciences Faculty, 16779 University of Salamanca, Plaza de los Caídos 1-5, 37008 Salamanca, Spain; ‡ X-ray Diffraction Service, Nucleus Platform, University of Salamanca, Plaza de los Caídos 1-5, 37008 Salamanca, Spain

## Abstract

The present report details the 1,3-dipolar cycloaddition between a chiral cyclic nitrone (**1**) and different carbonyl-type dipolarophiles, thus confirming the regiodivergence observed due to the cyclic nature of the dipolarophile source. An *ortho* regioselectivity is observed in the resulting isoxazolidines when the dipolarophile is acyclic, whereas the *meta* adducts are obtained from the cyclic dipolarophiles. This evidence is supported by both electronic calculations and X-ray diffraction.

## Graphical abstract

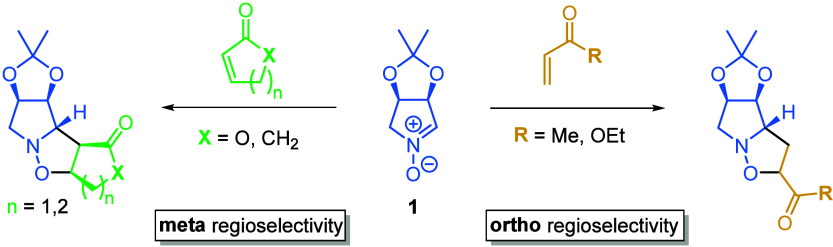

## Introduction

Since Huisgen established the concertedness of 1,3-dipolar cycloadditions in the 1960s,^1–4^ a plethora of discussions have emerged regarding the mechanism that governs this process.^5–8^ However, this has not prevented this reaction from becoming one of the most versatile synthetic methods for obtaining functionalized five-membered rings to date.^9–12^ A diverse range of 1,3-dipoles have been subjected to investigation in this type of reaction,^13^ with the nitrones emerging as a particularly intriguing case.^14–17^ This particular type of dipole leads to the formation of isoxazolidine rings,^18^ which have been demonstrated to be effective staring materials in diversity-oriented synthesis.^19,20^ In particular, cyclic nitrone **1**, used in this study, has been extensively used as a 1,3-dipole to afford bioactive scaffolds.^21^ Given the significance of isoxazolidines as versatile synthetic materials for the further synthesis of biological compounds,^12,23,24^ the development of scaffoldings of this nature has assumed a pivotal role.

Computational support has become an indispensable tool for the justification of experimental outcomes from 1,3-dipolar cycloadditions, illuminating the regioselectivity and stereoselectivity of these transformations.^25–27^ In this regard, the application of density functional theory (DFT) studies played a key role in confirming the concerted nature of the process along with experimental outcomes.^28,29^ Recently, our group has employed DFT to elucidate the underlying chemistry governing the 1,3-dipolar cycloaddition reactions between cyclic nitrones and *trans*- and *cis*-β-nitrostyrenes,^30^ thereby extending the earlier research conducted by Domingo and colleagues in this field.^31^

Despite the extensive research conducted over many years, several key questions regarding the regioselectivity and stereoselectivity of these reactions remain unanswered. Concerning 1,3-dipolar cycloadditions between cyclic nitrones and carbonyl-type dipolarophiles, the experimental outcomes vary, depending on the cyclic nature of the olefin. For cyclic carbonyl-type dipolarophiles, a *meta* direction of the electron-withdrawing group is observed,^15,32–34^ whereas for the acyclic ones, an *ortho* channel is preferred (Scheme 1).^35–39^ This behavior has been documented in numerous publications that have examined this type of cycloaddition, yet no definitive explanation for it has been provided.

**Scheme 1:**
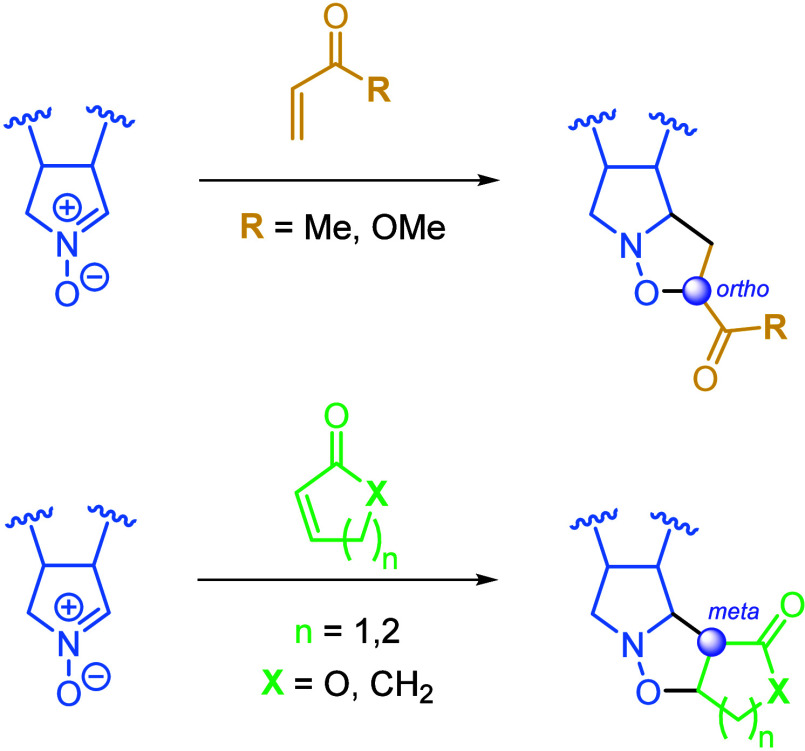
Regiodivergence Observed Due to the Cyclic Nature of the Dipolarophile

For this purpose, the 1,3-dipolar cycloaddition reaction between common starting material **1** and a range of diverse cyclic and acyclic carbonyl-type dipolarophiles was evaluated, and DFT calculations were also conducted in order to provide a computational explanation that would shed light on the experimental results, as has been the case in previous studies with these reactions.

## Results and Discussion

Our research group has a robust background in the utilization of cyclic nitrones for the synthesis of bicyclic isoxazolidine scaffolds via 1,3-dipolar cycloadditions. Several dipolarophiles have been evaluated to date, including vinyl sulfones and β-nitrostyrenes.^18,30^ However, in the case of carbonyl-type dipolarophiles, the results were found to be intriguing. The 1,3-dipolar cycloaddition reactions between nitrone **1** and these types of dipolarophiles have led to the discovery that the regioselectivity of the reaction is dependent on the cyclic nature of the original dipolarophile, as previously stated.

A series of studies were conducted with the aim of uncovering the aforementioned findings through the execution of these cycloadditions with an assortment of acyclic (methyl vinyl ketone and ethyl acrylate) and cyclic carbonyl-type dipolarophiles (2-cyclopenten-1-one, 2-cyclohexen-1-one, 2­(5*H*)-furanone, and 5,6-dihydro-2*H*-pyran-2-one). The objective was twofold: (1) to demonstrate the regiodivergence inherent to the cyclical nature of the dipolarophile and (2) to optimize these processes, given the demonstrated effectiveness of isoxazolidines in the subsequent synthesis of bioactive compounds. In order to achieve this objective, the proposed reactions were monitored by ^1^H NMR utilizing 1,3,5-trimethoxybenzene as an internal standard, with two variables being manipulated: temperature (25 or 85 °C) and time (6 or 24 h). The experimental design is outlined in Table 1, which depicts the four different isoxazoline compounds that could be obtained from these processes. The *syn* adducts (referring to the acetonide group) are not produced from this particular nitrone due to the steric influence of the acetonide group; instead, only *anti* adducts are observed.^18,30^ Moreover, the 1,3-dipolar cycloadditions were conducted in toluene, which has previously been demonstrated to be a more suitable solvent for these processes in kinetic terms.^18,30^ The outcomes of the experiments are listed in Table 1.

**Table 1:**
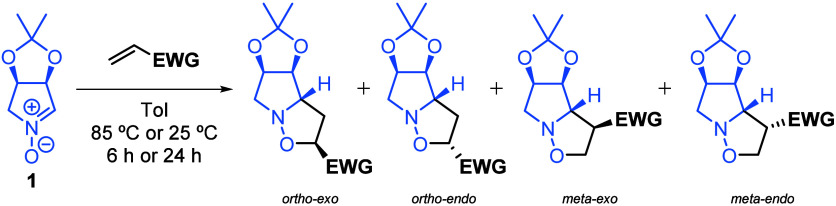

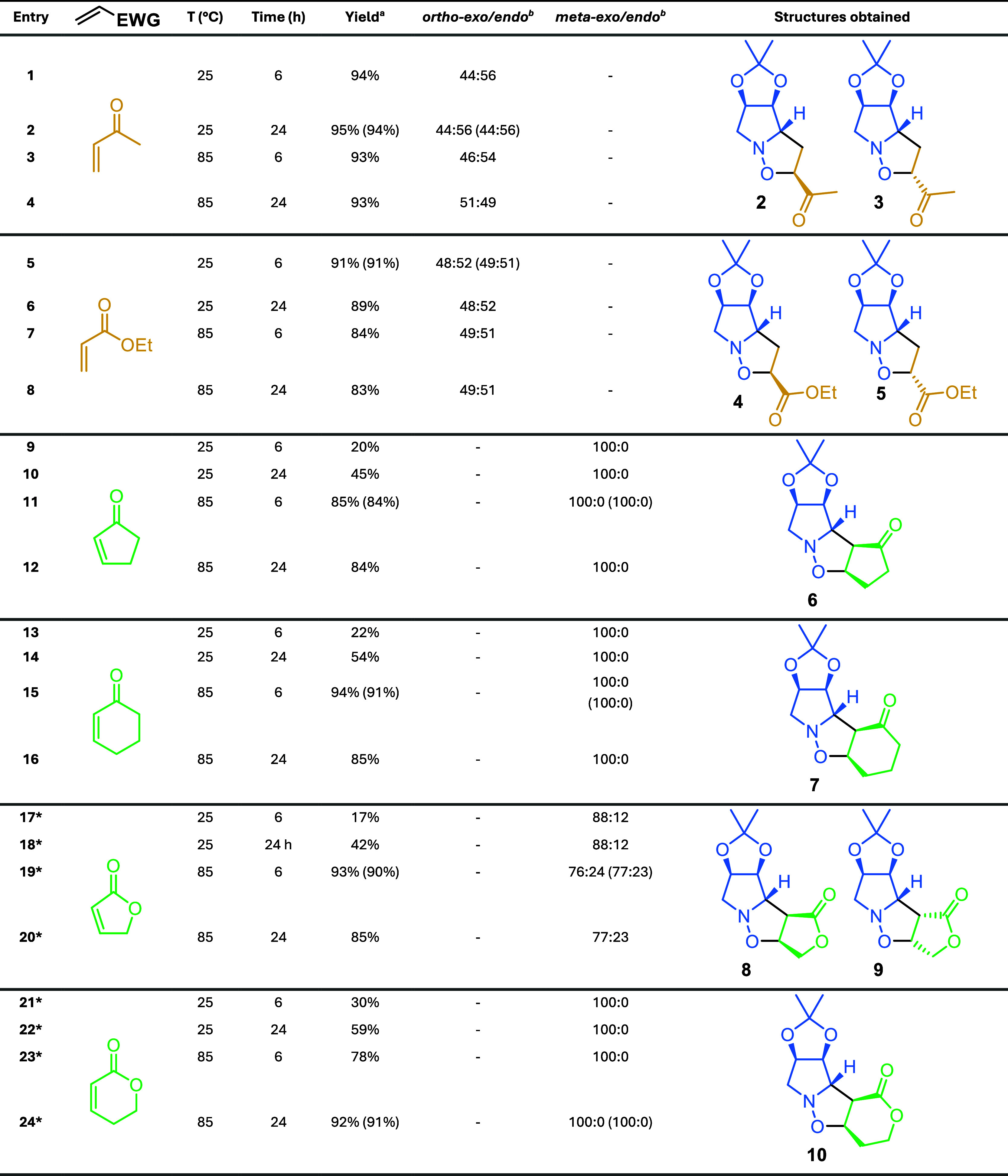
Cycloaddition Reaction of Nitrone **1** with Different Dipolarophiles (1.5 or *1.1 equiv) in Toluene-*d*_8_ (0.25 M) at 25 or 85 °C^c^

a Isolated yield in parentheses.

b Isolated ratio in parentheses.

c NMR yield recorded at 6 and 24 h. 1,3,5-Trimethoxybenzene used as an internal standard.

The experimental data presented in Table 1 suggest that the observed regiodivergence is inferred by the cyclic nature of the dipolarophilic source. When an acyclic carbonyl-type dipolarophile is employed in the reaction, the cycloadducts obtained present the electron-withdrawing group in the *ortho* position of the isoxazolidine ring (entries 1–8). Conversely, when a cyclic carbonyl-type dipolarophile is used instead, the products obtained exhibit *meta* regiochemistry (entries 9–24).

In the case of the cycloaddition reactions between **1** and acyclic carbonyl-type dipolarophiles (methyl vinyl ketone and ethyl acrylate), two distinct species were identified for each synthetic pathway, corresponding to *exo* (**2** and **4**) and *endo* adducts (**3** and **5**). Significant yields were achieved in both reactions, with a nearly equal 1:1 ratio of *exo* to *endo* adducts under all of the studied parameters. The optimal setup for the reaction with methyl vinyl ketone was found to be 25 °C for 24 h (entry 2), while the most favorable conditions for ethyl acrylate were determined to be 25 °C for 6 h (entry 5).

In contrast, when the cycloaddition is conducted with cyclic carbonyl-type dipolarophiles, only *exo* adducts are observed (**6**, **7**, and **10**), with the exception of 2­(5*H*)-furanone, which yields a mixture of *exo* and *endo* compounds in a 77:23 isolated ratio (**8** and **9**) (entry 19). In general, better yields were observed at 85 °C than at 25 °C in these processes, suggesting that the cycloadditions with cyclic carbonyl-type dipolarophiles require greater energy input. In the case of the reactions with 2-cyclopenten-1-one, 2-cyclohexen-1-one, and 2­(5*H*)-furanone, the most favorable conditions were determined to be 85 °C for 6 h (entries 11, 15, and 19, respectively), whereas for 5,6-dihydro-2*H*-pyran-2-one, the optimal setup was found to be 85 °C for 24 h (entry 24).

The cycloadditions between **1** and lactone-derived dipolarophiles such as 2­(5*H*)-furanone and 5,6-dihydro-2*H*-pyran-2-one were conducted with 1.1 equiv of the dipolarophile, as 1.5 equiv resulted in the formation of insoluble mixtures that impeded the monitoring of the reaction by ^1^H NMR. This type of reaction has been previously reported by our research group.^40^

It is noteworthy that the *meta*/*ortho* orientations differ between the two categories of dipolarophiles in these reactions as do the observed yields and *endo*:*exo* ratios. Higher yields were obtained at 25 °C for acyclic dipolarophiles, whereas cyclic dipolarophiles required higher temperatures (85 °C) to achieve significant yields (>80%), as shown in Table 1, suggesting that acyclic dipolarophiles are more reactive than cyclic ones. Regarding the product ratios, the *exo* adduct predominates for all cyclic dipolarophiles, while acyclic dipolarophiles yield an equal mixture of *endo* and *exo* isomers. These experimental results are further explained with computational support, as will be discussed in greater detail in the following section.

When the 1,3-dipolar cycloaddition between **1** and carbonyl-type dipolarophiles was conducted at a higher temperature (85 °C), a slight decrease in the yield of the reaction was observed over time (Table 1), with the yields at 6 and 24 h differing to some extent. This finding can be attributed to the decomposition of nitrone **1**, a phenomenon that occurs over time under heating through a cycloreversion process from the adducts formed as reported in previous experiments.^30,33^

The structures of the synthesized isoxazolidines were unambiguously confirmed through X-ray diffraction, which facilitated the determination of the stereochemistry of the products. This method proved to be crucial in overcoming the challenges of establishing the correct stereochemical configuration in five-membered rings solely on the basis of coupling constants (Figure 1).

**Figure 1:**
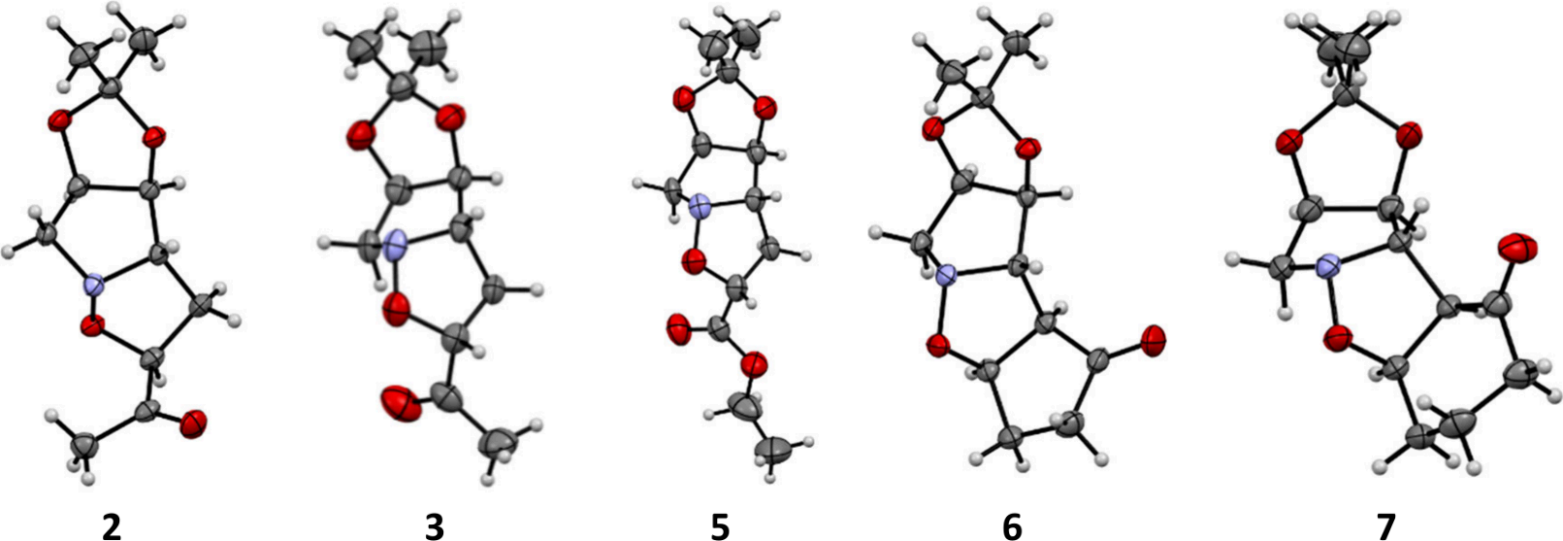
X-ray structures obtained from compounds **2**, **3**, and **5**–**7**. The X-ray structures of **8**–**10** were confirmed previously by our group.^40^

### Computational Analysis

As proposed previously, every 1,3-dipolar cycloaddition between the nitrone **1** and any dipolarophile may lead to four different regioisomeric products (labeling depicted in Figure 2).

**Figure 2:**
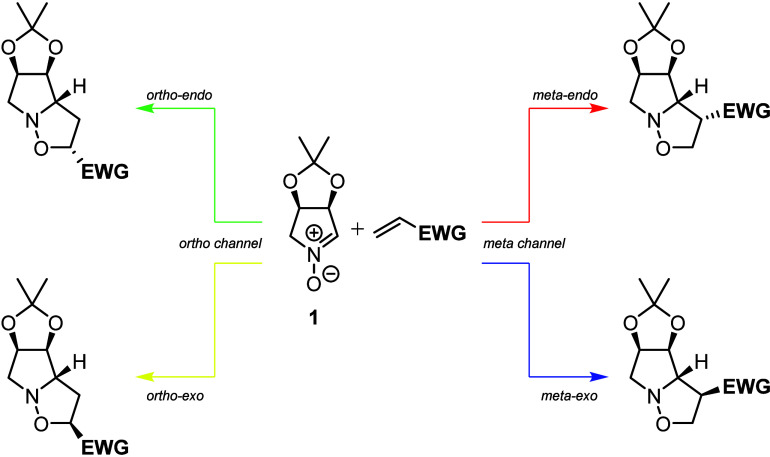
Regioisomeric outcomes of the 1,3-dipolar cycloadditions that were the objects of our work.

The theoretical results for the cycloaddition of nitrone **1** and methyl vinyl ketone (see Figure 3) demonstrate that *ortho* channels are the preferred reaction pathways. A 0.19 kcal/mol difference between the *exo* and *endo* approaches leads to a predicted 6:4 ratio between the two diastereomers that closely resembles that obtained experimentally (Table 1). This finding was attributed to the lower energy barrier of the transition states and the higher stability of the products, in comparison to the *meta* channels. A high degree of coherence between the theoretical and experimental outcomes is found, as only adducts **2** and **3** were obtained in this system.

**Figure 3:**
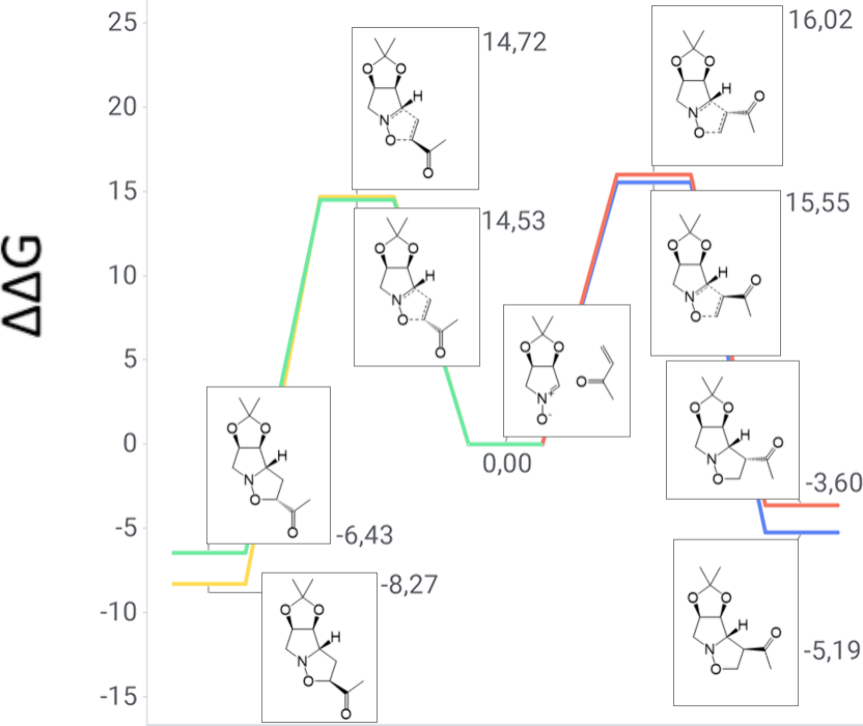
Gibbs free energy diagram of nitrone **1** and methyl vinyl ketone reaction channels (units of kilocalories per mole).

In the case of the cycloaddition system involving nitrone **1** and ethyl acrylate, both *exo* and *endo ortho* channels exhibit TS energies comparable to those of the *meta* ones (see Figure 4), with nearly isoenergetic paths. Although all products are exergonic, depicting a scenario in which all four routes are spontaneous, *ortho* isoxazolidines are the more likely pathway due to higher stability. However, as the difference between the *ortho-exo* and *ortho-endo* TS is 0.22 kcal/mol, a majority of the predicted products will be both *ortho* cycloadducts with a 6:4 ratio in a fashion similar to that of methyl vinyl ketone, close to the experimentally observed ratio of 1:1 (Table 1). The computational prediction is in agreement with the experimental observation, as only cycloadducts **4** and **5** were obtained.

**Figure 4:**
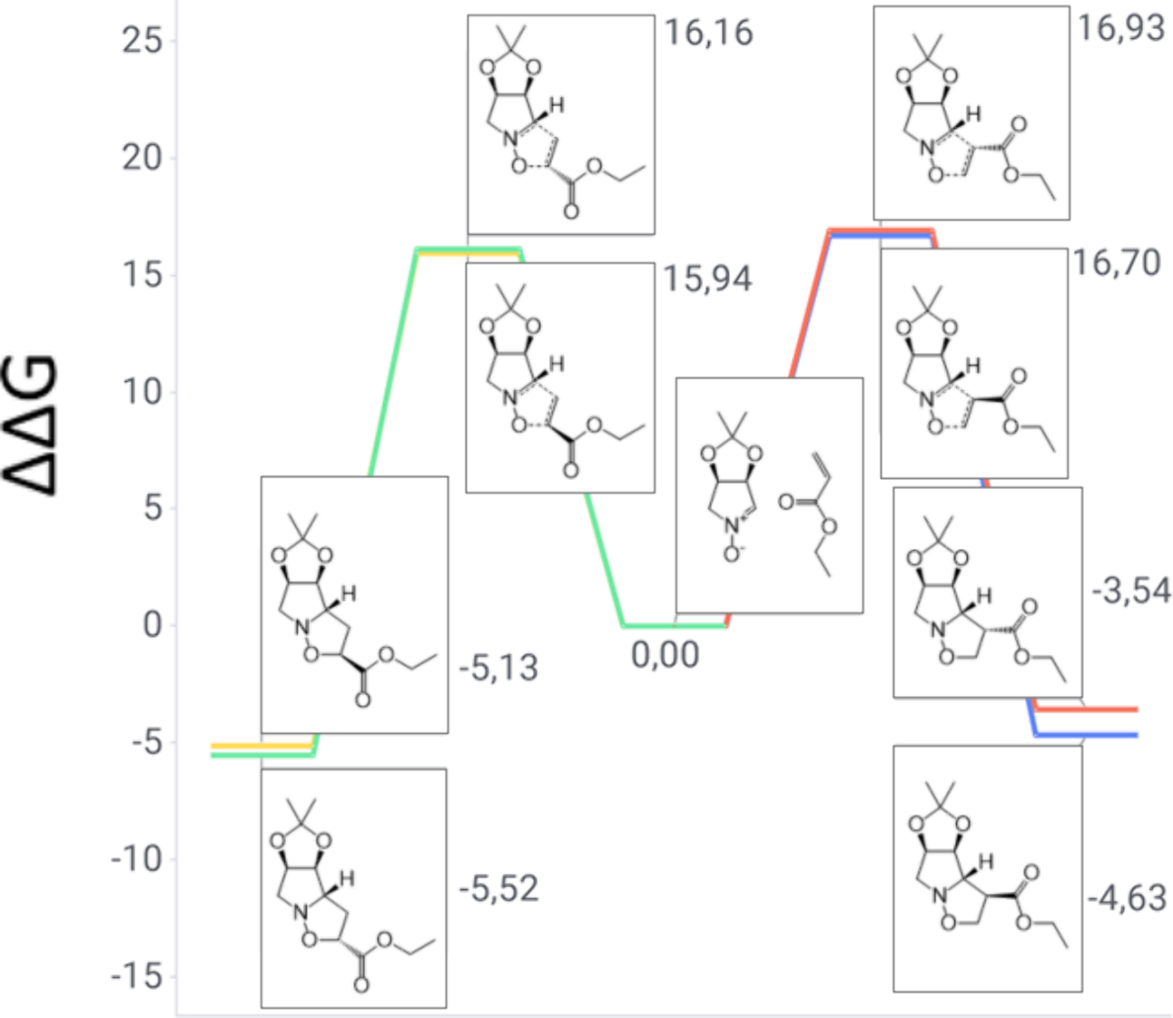
Gibbs free energy diagram of nitrone **1** and ethyl acrylate reaction channels (units of kilocalories per mole).

Regioisomeric reaction channels between nitrone **1** and cyclopentenone are depicted in Figure 5. The predicted product is the *meta-exo* derivative with a 17.48 kcal/mol TS barrier. This demonstrates the classical nitrone oxygen attack on the *meta* center of the Michael acceptor. Additionally, the product is the only one exhibiting an overall negative reaction free energy, thus indicating a spontaneous process. A similar outcome is detected when analyzing the behavior of cyclohexanone instead of cyclopentenone in the cycloadditive transformation (Figure 6). The primary reaction outcome is the *meta-exo* product, due to a TS barrier of 16.94 kcal/mol, which fulfills the classical attack between molecules as the driving force. As in the previous case, this product is the only spontaneous one in the regioisomeric group. These two features justify the experimental outcome, as only products **6** and **7** were observed in these processes.

**Figure 5:**
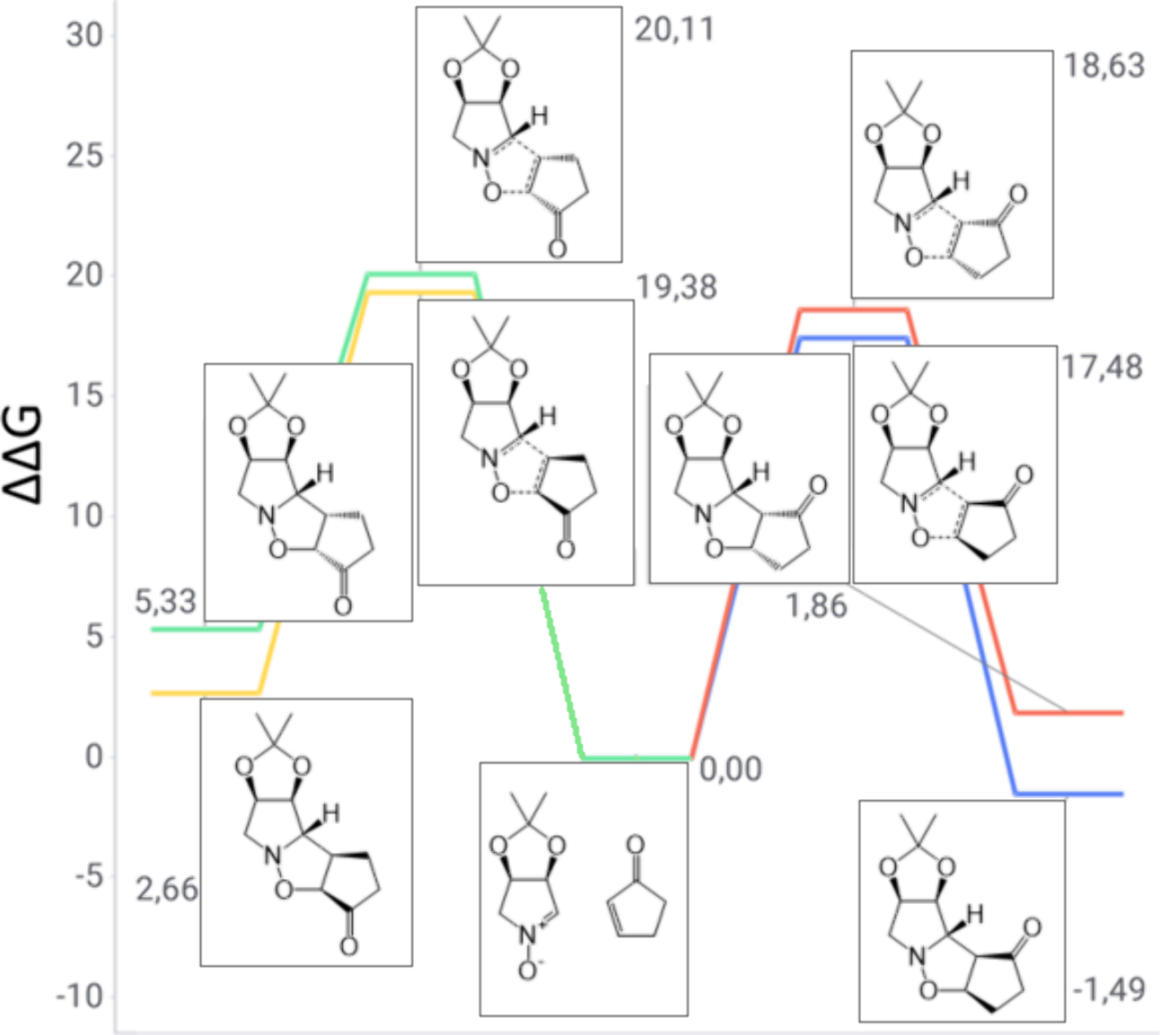
Gibbs free energy diagram of nitrone **1** and cyclopentanone reaction channels (units of kilocalories per mole).

**Figure 6:**
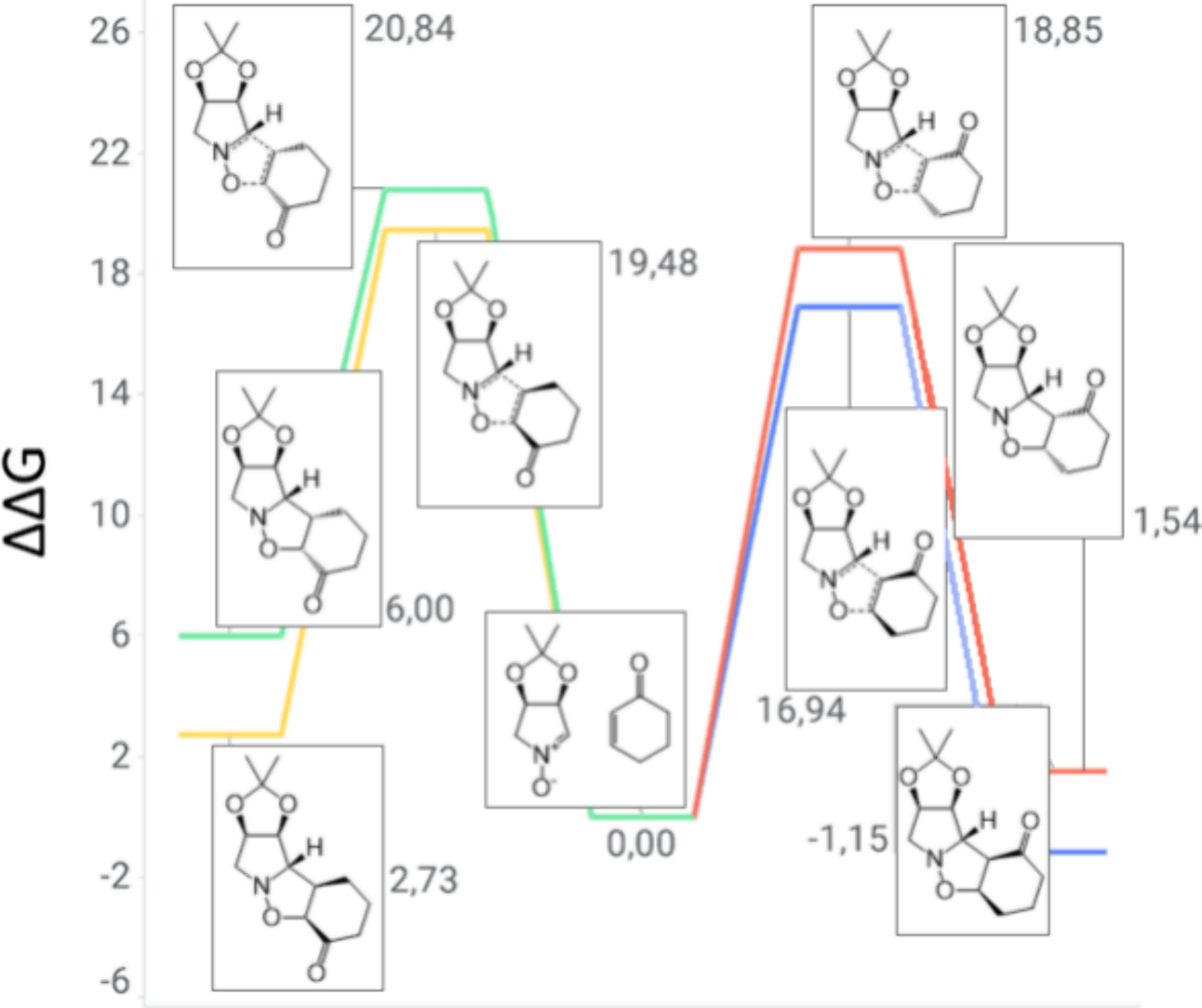
Gibbs free energy diagram of nitrone **1** and cyclohexanone reaction channels (units of kilocalories per mole).

Moving forward to lactone homologues, the reaction with 2­(5*H*)-furanone presents a deviation from the behaviors previously observed in cases involving cyclic dipolarophiles (Figure 7). As outlined in the Experimental Section, both *meta* channel TS spectra are found to be almost isoenergetic, exhibiting a marginal difference of 0.34 kcal/mol between saddle points. A straightforward Maxwell–Boltzmann population analysis of the available data yielded a 64:34 ratio predicted at 25 °C. It is evident that both transformations are exergonic, yielding nonreversible products at the conclusion of the process and hence transferring the predicted ratio from the transition states to the products. Despite the discrepancy between this ratio and the experimentally observed one (77:23 at 25 °C), it is noteworthy that the methodology employed was capable of detecting nearly isoenergetic transition states. A satisfactory degree of coherence is observed between the theoretical and experimental outcomes, as only adducts **8** and **9** were detected within this system.

**Figure 7:**
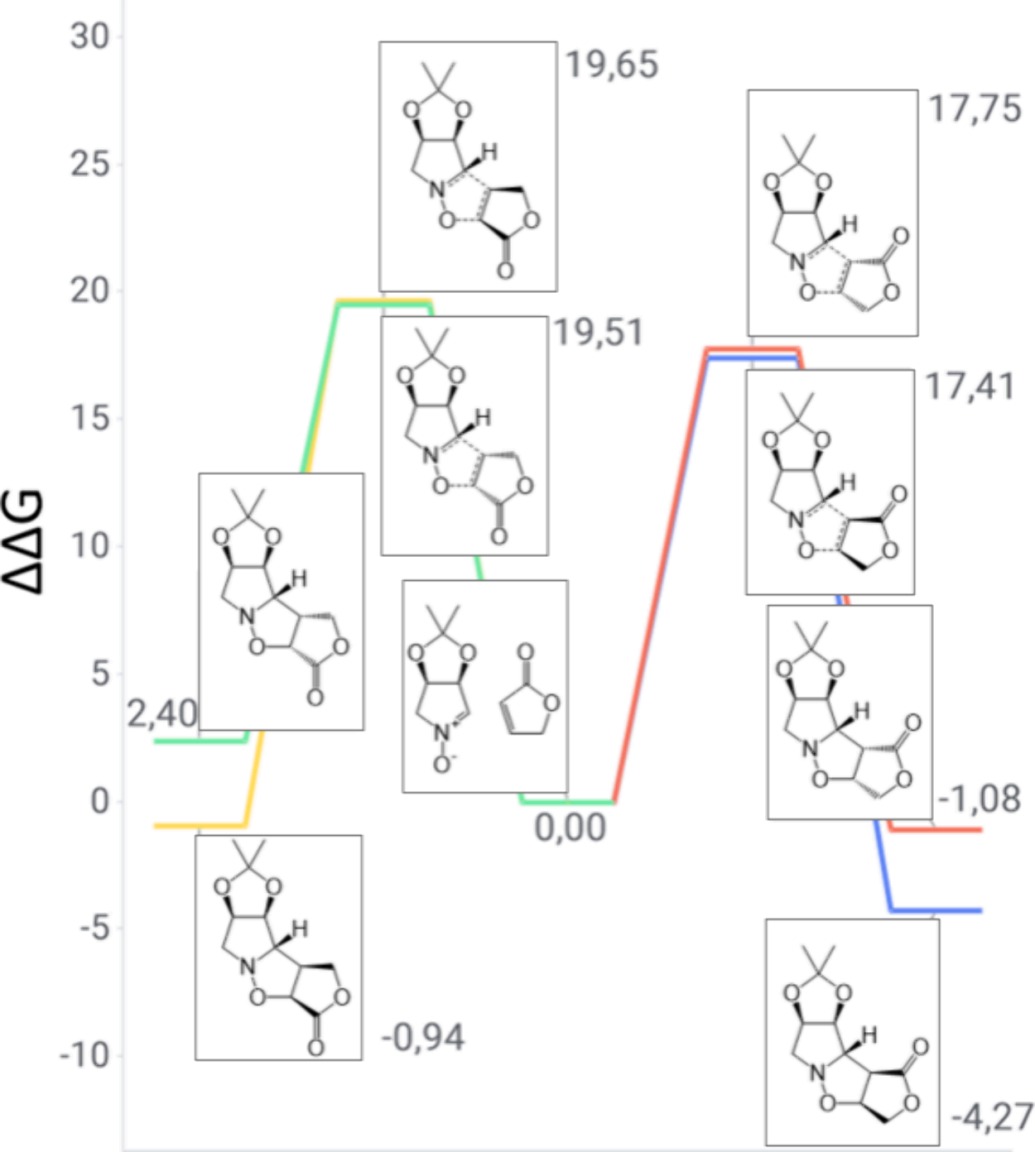
Gibbs free energy diagram of nitrone **1** and 2­(5*H*)-furanone reaction channels (units of kilocalories per mole).

The calculations of the reaction between 5,6-dihydro-2*H*-pyran-2-one and nitrone **1** reveal the same behavior as observed in the reactions with 2-cyclopenten-1-one and 2-cyclohexen-1-one (Figure 8). The *meta-exo* product is the preferred TS with a Gibbs free energy barrier of 16.55 kcal/mol, which is in close agreement with the experimental findings, as only product **10** was obtained. However, note that both *meta* channel transformations are now spontaneous. Nevertheless, only the *exo* isomer is observed due to a significant TS barrier difference between the two *meta* branches of approximately 2 kcal/mol.

**Figure 8:**
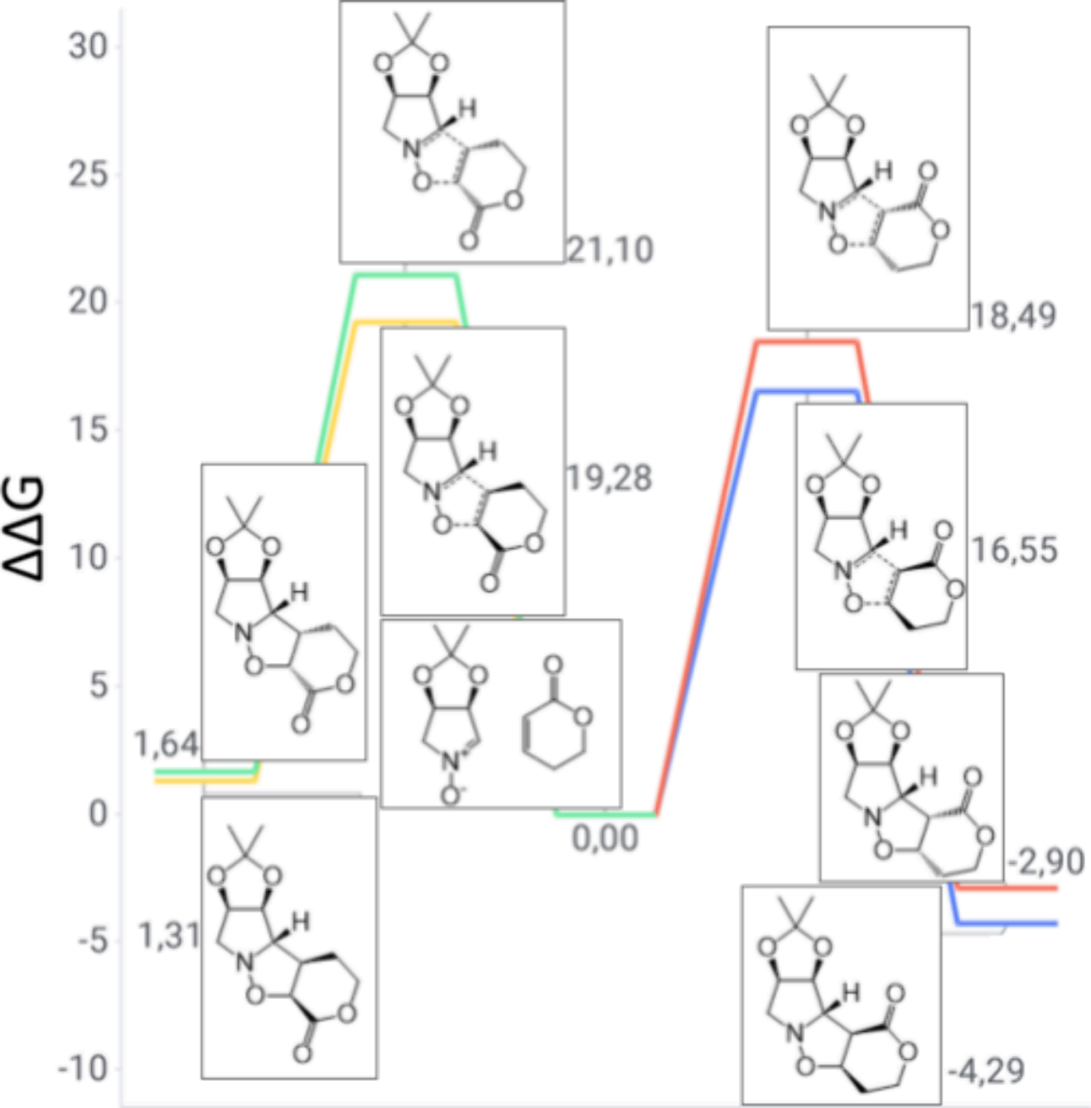
Gibbs free energy diagram of nitrone **1** and 5,6-dihydro-2*H*-pyran-2-one reaction channels (units of kilocalories per mole).

Classically, these kinds of observations are normally supported by the use of conceptual density functional (CDF) theory. In CDF theory, chemical reactivity is rationalized in terms of descriptors such as electronegativity, electronic chemical potential, hardness, and softness, incorporating the HOMO/LUMO energies as well from frontier molecular orbital theory (FMOT).

The electronic chemical potential (μ) is defined as the arithmetic mean of the HOMO and LUMO energies (μ = (ε_HOMO_ + ε_LUMO_)/2) and reflects the overall tendency of a molecule to donate electron density. In contrast, the global electrophilicity index (ω), given by the expression ω = μ^2^/(2η), provides a measure of the molecule’s ability to accept electrons. Here, η represents the chemical hardness, defined as the energy gap between the LUMO and HOMO orbitals (η = ε_LUMO_ – ε_HOMO_).

Global reactivity indices were calculated for nitrone **1** and a series of dipolarophiles, as summarized in Table 2. The electronic chemical potential (μ) of nitrone **1** (−0.142 au) is higher than that of all dipolarophiles considered, including methyl vinyl ketone, ethyl acrylate, and various cyclic enones and lactones. This suggests that nitrone **1** has a stronger tendency to donate electron density. Conversely, the global electrophilicity index (ω) is significantly higher for the dipolarophiles (ω = 0.057–0.068 au) than for nitrone **1** (ω = 0.046 au), indicating their greater capacity to accept electron density. These results support the interpretation that the electronic flow is directed from nitrone **1** toward the dipolarophile substrates, in agreement with a nucleophile–electrophile interaction model in which nitrone **1** acts as the nucleophile and the α,β-unsaturated carbonyl compounds behave as electrophilic dipolarophiles.

**Table 2:** HOMO and LUMO Energies, Electronic Chemical Potentials, Chemical Hardness Values, and Global Electrophilicity Indices of Reactants (in arbitrary units)

molecule	HOMO	LUMO	μ	η	ω
**1**	–0.251	–0.032	–0.142	0.219	0.046
methyl vinyl ketone	–0.275	–0.063	–0.169	0.211	0.068
ethyl acrylate	–0.300	–0.052	–0.176	0.248	0.063
cyclopentanone	–0.265	–0.049	–0.157	0.216	0.057
cyclohexanone	–0.264	–0.055	–0.160	0.210	0.061
2(5*H*)-furanone	–0.304	–0.049	–0.176	0.255	0.061
5,6-dihydro-2*H*-pyran-2-one	–0.295	–0.053	–0.174	0.242	0.062

After the global nucleophilic and electrophilic character was evaluated, a local analysis was carried out based on Fukui indices. The Fukui functions were calculated as *f* ^+^_k_ = ρ_k_(*N* + 1) – ρ_k_(*N*) and *f* ^–^_k_ = ρ_k_(*N*) – ρ_k_(*N* – 1), describing the susceptibility of a given atom to nucleophilic and electrophilic attack, respectively. The corresponding local indices of electrophilicity ω^+^_k_ and nucleophilicity ω^–^_k_ were obtained by weighting the Fukui values with the global electrophilicity of the molecule ω^±^_k_ = ωf^±^_k_.

As shown in Table 3, nitrone **1** exhibits its highest electrophilic susceptibility at C3 (ω^+^ = 0.020), while the most nucleophilic site corresponds to the O1 site (ω^–^ = 0.022). This suggests that O1 is the dominant nucleophilic center in the dipole. For all dipolarophiles evaluated, β-carbon C2 of the conjugated systems generally shows higher ω^+^ values than α-carbon C1, like methyl vinyl ketone (for C2, ω^+^ = 0.010) or ethyl acrylate (for C2, ω^+^ = 0.013). Cyclic enones also follow this trend, as seen in cyclopentanone, cyclohexanone, 2­(5*H*)-furanone, and 5,6-dihydro-2*H*-pyran-2-one.

**Table 3:**
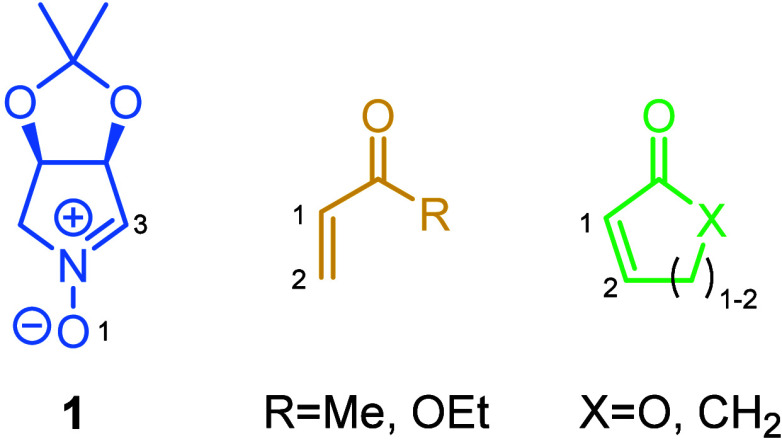
Fukui Functions and Local Electrophilicity Indices for the Centers of the Species Involved in the 1,3-Dipolar Cycloaddition (in arbitrary units)

	**1**		methyl vinyl ketone		ethyl acrylate		cyclopentanone	
	O1	C3	C1	C2	C1	C2	C1	C2
*f* ^+^	0.170	0.445	0.374	0.153	0.419	0.202	0.380	0.136
*f* ^–^	0.468	0.350	0.006	0.094	0.004	0.068	0.016	0.096
ω^+^	0.008	0.020	0.025	0.010	0.026	0.013	0.022	0.008
ω^–^	0.022	0.016	0.000	0.006	0.000	0.004	0.001	0.005

	cyclohexanone		2(5*H*)-furanone		5,6-dihydro-2*H*-pyran-2-one	
	C1	C2	C1	C2	C1	C2
*f* ^+^	0.340	0.128	0.399	0.218	0.383	0.196
*f* ^–^	0.014	0.085	0.126	0.202	0.173	0.232
ω^+^	0.021	0.008	0.024	0.013	0.024	0.012
ω^–^	0.001	0.005	0.008	0.012	0.011	0.014

Table 4 compiles the key bond distances (O–C and C–C) in the transition states and corresponding products of the 1,3-dipolar cycloadditions between nitrone **1** and a series of dipolarophiles. To evaluate the synchronicity of each pathway, the differences between the TS and product bond lengths were quantified and expressed as a percentage of asynchrony.

**Table 4:** Bond Distances, Bond Differences, and Average Final Bond Distances (in angstroms) of the Minimized Structures

dipolarophile	type	product	*d*(O1–C1)	*d*(C2–C3)	Δ*d*(O1–C1)	Δ*d*(C2–C3)	%
methyl vinyl ketone	TS	*meta-endo*	1.86	2.35	0.43	0.78	29
		*meta-exo*	1.92	2.31	0.49	0.75	21
		*ortho-endo*	2.03	2.69	0.59	1.15	32
		*ortho-exo*	2.06	2.29	0.66	0.76	7
	product	*meta-endo*	1.43	1.57			
		*meta-exo*	1.43	1.56			
		*ortho-endo*	1.44	1.54			
		*ortho-exo*	1.4	1.53			
ethyl acrylate	TS	*meta-endo*	1.92	2.29	0.49	0.72	19
		*meta-exo*	2.05	2.29	0.62	0.73	8
		*ortho-endo*	2.03	2.3	0.59	0.76	13
		*ortho-exo*	1.96	2.27	0.56	0.74	14
	product	*meta-endo*	1.43	1.57			
		*meta-exo*	1.43	1.56			
		*ortho-endo*	1.44	1.54			
		*ortho-exo*	1.4	1.53			
cyclopentanone	TS	*meta-endo*	2.21	1.94	0.78	0.41	31
		*meta-exo*	2.04	2.26	0.63	0.74	8
		*ortho-endo*	1.99	2.3	0.56	0.75	15
		*ortho-exo*	1.98	2.24	0.54	0.69	12
	product	*meta-endo*	1.43	1.53			
		*meta-exo*	1.41	1.52			
		*ortho-endo*	1.43	1.55			
		*ortho-exo*	1.44	1.55			
cyclohexanone	TS	*meta-endo*	2.21	1.95	0.79	0.41	32
		*meta-exo*	2.04	2.27	0.6	0.72	9
		*ortho-endo*	2.02	2.24	0.6	0.69	7
		*ortho-exo*	2.04	2.27	0.63	0.74	8
	product	*meta-endo*	1.42	1.54			
		*meta-exo*	1.44	1.55			
		*ortho-endo*	1.42	1.55			
		*ortho-exo*	1.41	1.53			
2(5*H*)-furanone	TS	*meta-endo*	2.2	1.96	0.77	0.42	29
		*meta-exo*	2.05	2.2	0.64	0.68	3
		*ortho-endo*	2.02	2.21	0.61	0.68	5
		*ortho-exo*	2.05	2.2	0.66	0.67	1
	product	*meta-endo*	1.43	1.54			
		*meta-exo*	1.41	1.52			
		*ortho-endo*	1.41	1.53			
		*ortho-exo*	1.39	1.53			
5,6-dihydro-2*H*-pyran-2-one	TS	*meta-endo*	2.21	1.98	0.8	0.44	29
		*meta-exo*	2.07	2.23	0.63	0.67	3
		*ortho-endo*	2.03	2.19	0.63	0.67	3
		*ortho-exo*	2.07	2.23	0.63	0.68	4
	product	*meta-endo*	1.41	1.54			
		*meta-exo*	1.44	1.56			
		*ortho-endo*	1.4	1.52			
		*ortho-exo*	1.44	1.55			

The reactions exhibit moderate to significant asynchrony, with values ranging from 1% to 32%. This indicates that the two bonds forming the isoxazolidine ring do not evolve in a perfectly synchronized fashion during the cycloaddition, and thus, the mechanisms are not fully concerted. In general, the *meta-endo* pathways tend to be more asynchronous than their *meta-exo* counterparts. For instance, *meta-endo* methyl vinyl ketone shows 29% asynchrony versus 21% for the *meta-exo* form. This trend is consistent across other systems: ethyl acrylate (19% vs 8%), cyclopentanone (31% vs 8%), cyclohexanone (32% vs 9%), 2­(5*H*)-furanone (29% vs 3%), and 5,6-dihydro-2*H*-pyran-2-one (29% vs 3%). These differences suggest that *endo* pathways involve a more delayed or uneven bond formation, often with the C–C bond lagging behind the O–C bond due to steric constraints or electronic repulsions.

The *ortho-endo* reactions consistently show the highest asynchrony, reaching 32% in the methyl vinyl ketone. This is followed by cyclopentanone (15%) and ethyl acrylate (13%). These values indicate that in *ortho-endo* trajectories the approach geometry introduces additional strain or unfavorable interactions—particularly between the nitrone oxygen and the carbonyl group of the dipolarophile—which may hinder a concerted, balanced formation of the two bonds.

In contrast, the *ortho-exo* pathways are generally among the most synchronous configurations but with more variability than initially expected. While 2­(5*H*)-furanone exhibits the lowest asynchrony overall (1%) and that of 5,6-dihydro-2*H*-pyran-2-one remains low (4%), other systems such as ethyl acrylate and cyclopentanone reach values of 14% and 12%, respectively. These variations suggest that although *exo* orientations can favor a more balanced orbital alignment, additional factors—such as ring size, conjugation, and substituent effects—also influence the degree of bond formation synchrony in these transition states.

Taken together, both Fukui indices and degrees of synchronicity predict the classical two-center interaction between O1 of nitrone **1** and β-carbon C2 of the dipolarophiles. These regioselective interactions would lead to the formation of *meta* channel 5-substituted isoxazolidines as the major cycloadducts but offer a limited explanation about the observed *ortho* channel adducts. Not all predicted preferences based on Fukui indices are consistent with experimental observations. This discrepancy underscores the limitations of relying solely on ground state electronic descriptors for regioselectivity prediction. As these are neither quantum-mechanical observables nor explicit functionals of the electron density, their validity is under investigation.

In a closely related case,^41^ the reaction of pyrroline 1-oxide with ethyl acrylate was theoretically investigated by means of TS analyses and quantum chemical topology. First, it is noted that the level of theory used impacts the *exo*:*endo* ratio. Second, carrying out electron localization function calculations (ELF)^42^ from bonding evolution theory (BET),^43^ the authors observed differential behavior of the different electron basins in the course of the intrinsic reaction pathway (IRC), justifying the likelihood of the *ortho* channels; however, the origins in regiochemical reactivity for this specific system have not yet been described. Work is in progress in this regard in our group.

## Conclusions

The different regioselectivities observed in the 1,3-dipolar cycloaddition reactions between cyclic nitrones and carbonyl-type dipolarophiles are herein demonstrated with a particular case (nitrone **1**). The optimization studies verify the regiodivergence observed in previous reports, confirming unequivocally the structures formed by X-ray diffraction. The 1,3-dipolar cycloaddition performed using acyclic carbonyl-type dipolarophiles led to the formation of *ortho*-substituted isoxazolidines (**2**–**5**), whereas a cyclic one is utilized to produce the *meta* adducts (**6**–**10**).

Computational studies were essential to rationalize this regiodivergence. Electronic structure calculations demonstrated that both kinetic and thermodynamic factors are responsible for the formation of the experimentally observed isoxazolidines, aligning well with the empirical data. Importantly, the joint analysis of Fukui indices and the degree of synchronicity supports the classical two-center interaction between O1 of nitrone **1** and the β-carbon (C2) of the dipolarophile. This would typically favor the formation of *meta* channel 5-substituted isoxazolidines as the major products. However, such descriptors fall short of explaining the experimentally observed *ortho* channel products in reactions with acyclic dipolarophiles. This discrepancy highlights the limitations of relying solely on ground state reactivity indices, such as Fukui functions, to predict regioselectivity outcomes. These indices are neither quantum-mechanical observables nor explicit functionals of the electron density, and their predictive validity remains under critical evaluation.

Altogether, the study reinforces the idea that DFT calculations, particularly those incorporating transition state analysis, are indispensable for unraveling the complex mechanistic behavior of 1,3-dipolar cycloadditions and capture effects that go beyond frontier molecular orbital theory.

Further computational investigations are being pursued to uncover the theoretical constraints that govern the experimentally observed selectivity patterns.

## Experimental Section

### Experimental Methods

Unless otherwise stated, all chemicals were purchased as the highest purity commercially available and used without further purification. IR spectra were recorded on a BOMEM 100 FT-IR or an AVATAR 370 FT-IR Thermo Nicolet spectrophotometer. ^1^H and ^13^C NMR spectra were recorded at room temperature using either a Varian Mercury 200 MHz or a Bruker Avance NEO 400 MHz instrument with a Prodigy CPPBBO BB-H&F z-gradient cryo-probe spectrometer (NMR Service, NUCLEUS, University of Salamanca). Chemical shifts (δ) are given in parts per million with the solvent signal as the internal standard unless otherwise stated (CHCl_3_ 7.26 ppm for ^1^H NMR, CDCl_3_ 77.0 ppm for ^13^C NMR), and coupling constants (*J*) are given in hertz. MS was performed on a VG-TS 250 spectrometer with an ionizing voltage of 70 eV. Mass spectra are presented as *m*/*z* (% relative intensity). HRMS were recorded on a VG Platform (Fisons) spectrometer using chemical ionization (ammonia as gas) or fast atom bombardment (FAB). For some of the samples, a QSTAR XL spectrometer was employed for electrospray ionization (ESI). A triple quadrupole (TQ) was used as a mass analyzer. Optical rotations were determined on a PerkinElmer 241 polarimeter in a 1 dm cell. Hexane was distilled prior to use.

### Computational Details

QM-type transition state studies were carried out using NWChem^44^ (as the QM algorithm) and XTB^45^ (as the semiempirical one) engines nested in the autodE package.^46^ autodE is a double-ended, fully automated transition state (TS) search algorithm with conformational screening of the TS, reactants, and products. autodE requires only 1D SMILES representation of the starting materials and products, as well as the appropriate level of theory, to conduct the conformational analysis and optimization of the starting and ending species. A suitable mapping of the reactive centers is carried out, leading to prospective TS saddle points. Forward conformational analysis and optimization yield suitable TS entities. For this work, the standard GFN2-XTB method^45^ for XTB (defined as low_opt in autodE) and the PBE0-D3BJ/def2-SVP method^47,48^ (defined as opt in autodE) for NWChem were used as conformational search/optimization levels of theory. Finally, all isolated entities were subjected to single-point accurate DFT energy calculations by means of PBE0-D3BJ/def2-TZVP. All calculations were carried out at 25 °C in a toluene solution through the polarizable continuum model (PCM) integrated in NWChem. Posterior vibrational normal-mode analysis was carried out to verify that reactants and products were stationary points (zero imaginary frequencies), and transition structures had one and only one imaginary frequency. Fukui indices were calculated for the collection of reagents (nitrone **1** and dipolarophiles).

### Synthesis of Starting Material (3*S*,4*R*)-3,4-Isopropylidenedioxypyrrolidine-1-oxide (**1**)

Nitrone **1** was synthesized following a previously reported procedure.^49^

### General Procedure for Cycloaddition Optimization

To a solution of nitrone **1** (100 mg, 0.636 mmol, 1 equiv) in toluene-*d*_8_ (2.55 mL, 0.25 M) was added the respective diplolarophile (0.954 mmol, 1.5 equiv, or 0.700 mmol, 1.1 equiv). The mixture was stirred at 25 or 85 °C, and the NMR yield was obtained at 6 and 24 h using 1,3,5-trimethoxybenzene (1 equiv) as the internal standard.

### Cycloaddition of **1** with Methyl Vinyl Ketone

To a solution of nitrone **1** (100 mg, 0.636 mmol) in toluene (2.55 mL) was added 77.4 μL of methyl vinyl ketone (0.954 mmol). The mixture was stirred at 25 °C for 24 h. The solution was concentrated in vacuo, and the resulting crude product was purified by flash chromatography on silica gel (hexane/EtOAc, 9:1 to 1:1) to obtain isoxazolidines **2** (59.8 mg, 41%) and **3** (76.1 mg, 53%) as white crystalline solids.

#### 1-((2*S*,3a*S*,4*S*,5*R*)-4,5-Isopropylidenedioxyhexahydropyrrolo­[1,2-*b*]­isoxazol-2-yl)­ethan-1-one (**2**)

[α]_D_^20^ = +16.8 (*c* = 1.0, CHCl_3_); IR (cm^–1^) 2988, 2921, 1707, 1374, 1353, 1214, 1075, 868, 795; ^1^H NMR (400 MHz, CDCl_3_) δ 4.98 (1H, td, *J* = 6.2, 4.5 Hz, H-5), 4.56 (1H, dd, *J* = 6.2, 4.0 Hz, H-4), 4.35 (1H, dd, *J* = 9.4, 4.3 Hz, H-2), 3.67 (1H, dt, *J* = 7.9, 4.0 Hz, H-3a), 3.55 (1H, dd, *J* = 14.0, 6.2 Hz, H_b_-6), 3.27 (1H, dd, *J* = 14.0, 4.5 Hz, H_a_-6), 2.74 (1H, ddd, *J* = 13.1, 7.9, 4.3 Hz, H_a_-3), 2.41 (1H, ddd, *J* = 13.1, 9.4, 4.0 Hz, H_b_-3), 2.26 (3H, s, CO-Me), 1.51 (3H, s, C-Me_b_), 1.32 (3H, s, C-Me_a_); ^13^C­{^1^H} NMR (100 MHz, CDCl_3_) δ 210.6 (CO), 113.7 (C-Me_2_), 85.9 (CH-4), 81.5 (CH-2), 81.3 (CH-5), 71.0 (CH-3a), 61.2 (CH_2_-6), 36.2 (CH_2_-3), 27.2 (C-Me_b_), 25.8 (CH_3_), 25.1 (C-Me_a_); HRMS (ESI) *m*/*z* [M + H]^+^ calcd for C_11_H_18_NO_4_ 228.1230, found 228.1238.

#### 1-((2*R*,3a*S*,4*S*,5*R*)-4,5-Isopropylidenedioxyhexahydropyrrolo­[1,2-*b*]­isoxazol-2-yl)­ethan-1-one (**3**)

[α]_D_^20^ = −24.7 (*c* = 0.9, CHCl_3_); IR (cm^–1^) 2988, 2938, 1713, 1657, 1553, 1381, 1214, 1090, 863, 753; ^1^H NMR (400 MHz, CDCl_3_) δ 4.94 (1H, ddd, *J* = 6.4, 5.2, 4.1 Hz, H-5), 4.55 (1H, dd, *J* = 6.4, 2.2 Hz, H-4), 4.45 (1H, dd, *J* = 8.7, 8.1 Hz, H-2), 3.82 (1H, ddd, *J* = 8.1, 6.6, 2.2 Hz, H-3a), 3.35 (1H, dd, *J* = 12.4, 5.2 Hz, H_b_-6), 3.32 (1H, dd, *J* = 12.4, 4.1 Hz, H_a_-6), 2.68 (1H, ddd, *J* = 13.0, 8.7, 8.1 Hz, Ha-3), 2.23 (1H, m, H_b_-3), 2.23 (3H, s, CO-Me), 1.50 (3H, s, C-Me_b_), 1.30 (3H, s, C-Me_a_); ^13^C­{^1^H} NMR (100 MHz, CDCl_3_) δ 206.6 (CO), 113.1 (C-Me_2_), 84.4 (CH-4), 83.5 (CH-2), 80.4 (CH-5), 71.8 (CH-3a), 61.0 (CH_2_-6), 35.3 (CH_2_-3), 27.3 (CH_3_), 26.9 (C-Me_b_), 25.0 (C-Me_a_); HRMS (ESI) *m*/*z* [M + H]^+^ calcd for C_11_H_18_NO_4_ 228.1230, found 228.1234.

### Cycloaddition of **1** with Ethyl Acrylate

To a solution of nitrone **1** (100 mg, 0.636 mmol) in toluene (2.55 mL) was added 103.9 μL of ethyl acrylate (0.954 mmol). The mixture was stirred at 25 °C for 6 h. The solution was concentrated in vacuo, and the resulting crude product was purified by flash chromatography on silica gel (hexane/EtOAc, 9:1 to 1:1) to obtain isoxazolidines **4** (72.9 mg, 45%) as an amorphous white solid and **5** (75.9 mg, 46%) as a white crystalline solid.

#### Ethyl (2*S*,3a*S*,4*S*,5*R*)-4,5-Isopropylidenedioxyhexahydropyrrolo­[1,2-*b*]­isoxazole-2-carboxylate (**4**)

[α]_D_^20^ = +29.4 (*c* = 0.4, CHCl_3_); IR (cm^–1^) 2984, 2932, 1744, 1457, 1374, 1275, 1206, 1073, 857; ^1^H NMR (400 MHz, CDCl_3_) δ 4.98 (1H, td, *J* = 6.0, 4.2 Hz, H-5), 4.58 (2H, m, H-2 and H-4), 4.23 (2H, m, O-CH_2_CH_3_), 3.79 (1H, dt, *J* = 7.7, 4.6 Hz, H-3a), 3.55 (1H, dd, *J* = 14.0, 6.0 Hz, H_b_-6), 3.33 (1H, dd, *J* = 14.0, 4.2 Hz, H_a_-6), 2.79 (1H, ddd, *J* = 13.0, 7.7, 4.5 Hz, H_a_-3), 2.52 (1H, ddd, *J* = 13.0, 9.3, 4.6 Hz, H_b_-3), 1.51 (3H, s, C-Me_b_), 1.32 (3H, s, C-Me_a_), 1.29 (3H, t, *J* = 7.2 Hz, O-CH_2_ CH_3_); ^13^C­{^1^H} NMR (100 MHz, CDCl_3_) δ 171.5 (COOEt), 113.7 (C-Me_2_), 85.5 (CH-4), 81.1 (CH-5), 75.3 (CH-2), 71.1 (CH-3a), 61.7 (O-CH_2_CH_3_), 61.4 (CH_2_-6), 37.2 (CH_2_-3), 27.1 (C-Me_b_), 25.2 (C-Me_a_), 14.3 (O-CH_2_ CH_3_); HRMS (ESI) *m*/*z* [M + H]^+^ calcd for C_11_H_20_NO_5_ 258.1336, found 258.1340.

#### Ethyl (2*R*,3a*S*,4*S*,5*R*)-4,5-Isopropylidenedioxyhexahydropyrrolo­[1,2-*b*]­isoxazole-2-carboxylate (**5**)

[α]_D_^20^ = −2.9 (*c* = 0.6, CHCl_3_); IR (cm^–1^) 2984, 2938, 1748, 1470, 1380, 1272, 1210, 1069, 1034, 868, 737; ^1^H NMR (400 MHz, CDCl_3_) δ 5.01 (1H, td, *J* = 6.1, 3.4 Hz, H-5), 4.65 (1H, dd, *J* = 6.1, 2.2 Hz, H-4), 4.54 (1H, dd, *J* = 8.3, 7.6 Hz, H-2), 4.22 (2H, m, O-CH_2_CH_3_), 3.86 (1H, m, H-3a), 3.60 (1H, dd, *J* = 13.6, 6.1 Hz, H_b_-6), 3.42 (1H, dd, *J* = 13.6, 3.4 Hz, H_a_-6), 2.82 (1H, dt, *J* = 13.0, 8.3 Hz, H_a_-3), 2.32 (1H, ddd, *J* = 13.0, 7.6, 6.5 Hz, H_b_-3), 1.51 (3H, s, C-Me_b_), 1.31 (3H, s, C-Me_a_), 1.29 (3H, t, *J* = 7.1 Hz, O-CH_2_ CH_3_); ^13^C­{^1^H} NMR (100 MHz, CDCl_3_) δ 171.4 (COOEt), 113.2 (C-Me_2_), 83.9 (CH-4), 80.4 (CH-5), 77.4 (CH-2), 71.8 (CH-3a), 61.8 (O-CH_2_CH_3_), 61.0 (CH_2_-6), 37.4 (CH_2_-3), 26.9 (C-Me_b_), 25.1 (C-Me_a_), 14.3 (O-CH_2_ CH_3_); HRMS (ESI) *m*/*z* [M + H]^+^ calcd for C_11_H_20_NO_5_ 258.1336, found 258.1341.

### Cycloaddition of **1** with 2-Cyclopenten-1-one

To a solution of **1** (100 mg, 0.636 mmol) in toluene (2.55 mL) was added 80 μL of 2-cyclopenten-1-one (0.954 mmol). The mixture was then stirred at 85 °C for 6 h. Then, the solution was concentrated in vacuo, and the resulting crude product was purified by flash chromatography on silica gel (hexane/EtOAc, 9:1 to 1:1) to obtain isoxazolidine **6** in 84% yield (127.9 mg) as a white crystalline solid.

#### (2*R*,3*R*,3a*S*,4*S*,5*R*)-4,5-Isopropylidenedioxyoctahydro-1*H*-cyclopenta­[*d*]­pyrrolo­[1,2-*b*]­isoxazol-8-one (**6**)

[α]_D_^20^ = −130.6 (*c* = 0.4, CHCl_3_); IR (cm^–1^) 2986, 2925, 1734, 1376, 1268, 1212, 1165, 1058, 1025, 928, 866; ^1^H NMR (400 MHz, CDCl_3_) δ 4.89 (2H, m, H-2 and H-5), 4.67 (1H, dd, *J* = 6.5, 2.7 Hz, H-4), 3.76 (1H, t, *J* = 2.7 Hz, H-3a), 3.35 (1H, dd, *J* = 13.4, 3.5 Hz, H_a_-6), 3.32 (1H, dd, *J* = 13.4, 5.2 Hz, H_b_-6), 2.97 (1H, dd, *J* = 6.3, 2.7 Hz, H-3), 2.55 (1H, dt, *J* = 18.4, 9.9 Hz, H_b_-9), 2.27 (1H, m, H_a_-9), 2.15 (2H, m, H-10), 1.49 (3H, s, C-Me_b_), 1.30 (3H, s, C-Me_a_); ^13^C­{^1^H} NMR (100 MHz, CDCl_3_) δ 216.4 (CO), 113.3 (C-Me_2_), 84.5 (CH-4), 80.2 (CH-5), 79.2 (CH-2), 76.7 (CH-3a), 59.9 (CH_2_-6), 59.6 (CH-3), 36.1 (CH_2_-9), 26.9 (C-Me_b_), 26.7 (CH_2_-10), 25.2 (C-Me_a_); HRMS (ESI) *m*/*z* [M + H]^+^ calcd for C_12_H_18_NO_4_ 240.1230, found 240.1223.

### Cycloaddition of **1** with 2-Cyclohexen-1-one

To a solution of nitrone **1** (100 mg, 0.636 mmol) in toluene (2.55 mL) was added 92.4 μL of 2-cyclohexen-1-one (0.954 mmol). The mixture was then stirred at 85 °C for 6 h. The solution was concentrated in vacuo, and the resulting crude product was purified by flash chromatography on silica gel (hexane/EtOAc, 9:1 to 1:1) to obtain isoxazolidine **7** in 91% yield (146.6 mg) as a white crystalline solid.

#### (2*R*,3*R*,3a*S*,4*S*,5*R*)-4,5-Isopropylidenedioxydecahydrobenzo­[*d*]­pyrrolo­[1,2-*b*]­isoxazole-8-one (**7**)

[α]_D_^20^ = −41.8 (*c* = 0.2, CHCl_3_); IR (cm^–1^) 2986, 2940, 1707, 1381, 1235, 1210, 1067, 859; ^1^H NMR (400 MHz, CDCl_3_) δ 4.83 (1H, td, *J* = 6.1, 2.3 Hz, H-5), 4.68 (1H, dd, *J* = 6.1, 1.3 Hz, H-4), 4.58 (1H, dt, *J* = 7.2, 4.7 Hz, H-2), 4.03 (1H, d, *J* = 7.2 Hz, H-3a), 3.47 (1H, dd, *J* = 12.9, 2.3 Hz, H_a_-6), 3.08 (1H, ddd, *J* = 12.9, 6.1, 0.6 Hz, H_b_-6), 2.89 (1H, t, *J* = 7.2 Hz, H-3), 2.49 (1H, dt, *J* = 17.3, 5.5 Hz, H_b_-9), 2.32 (1H, dddd, *J* = 17.3, 9.6, 5.9, 0.8 Hz, H_a_-9), 2.00 (1H, m, H_b_-10), 1.88 (2H, m, H-11), 1.73 (1H, m, H_a_-10), 1.50 (3H, s, C-Me_b_), 1.29 (3H, s, C-Me_a_); ^13^C­{^1^H} NMR (100 MHz, CDCl_3_) δ 208.2 (CO), 112.3 (C-Me_2_), 82.6 (CH-4), 79.2 (CH-5), 77.0 (CH-2), 73.7 (CH-3a), 60.7 (CH_2_-6), 57.4 (CH-3), 39.2 (CH_2_-9), 27.2 (CH_2_-11), 26.7 (C-Me_b_), 25.1 (C-Me_a_), 18.6 (CH_2_-10); ESI-HRMS calcd for C_13_H_20_NO_4_ (M + H)^+^ *m*/*z* 254.1387, found *m*/*z* 254.1395; HRMS (ESI) *m*/*z* [M + H]^+^ calcd for C_11_H_20_NO_5_ 258.1336, found 258.1340.

### Cycloaddition of **1** with 2­(5*H*)-Furanone

To a solution of nitrone **1** (100 mg, 0.636 mmol) in toluene (2.55 mL) was added 49.7 μL of 2­(5*H*)-furanone (0.70 mmol). The mixture was then stirred at 85 °C for 6 h. The solution was concentrated in vacuo, and the resulting crude product was purified by flash chromatography on silica gel (hexane/EtOAc, 9:1 to 1:1) to obtain isoxazolidines **8** (106.4 mg, 69%) and **9** (31.7 mg, 21%) as white crystalline solids.

#### γ-Lactone of (2*S*,3*R*,3a*S*,4*S*,5*R*)-2-Hydroxymethyl-4,5-isopropylidenedioxyhexahydropyrrolo­[1,2-*b*]­isoxazol-3-carboxylic Acid (**8**)

The spectroscopic data have been reported previously.^40^

#### γ-Lactone of (2*R*,3*S*,3a*S*,4*S*,5*R*)-2-Hydroxymethyl-4,5-isopropylidenedioxyhexahydropyrrolo­[1,2-*b*]­isoxazol-3-carboxylic Acid (**9**)

The spectroscopic data have been reported previously.^40^

### Cycloaddition of **1** with 5,6-Dihydro-2*H*-pyran-2-one

To a solution of nitrone **1** (100 mg, 0.636 mmol) in toluene (2.55 mL) was added 92.4 μL of 5,6-dihydro-2*H*-pyran-2-one (0.70 mmol). The mixture was then stirred at 85 °C for 24 h. The solution was concentrated in vacuo, and the resulting crude product was purified by flash chromatography on silica gel (hexane/EtOAc, 9:1 to 1:1) to obtain isoxazolidine **10** in 91% yield (147.8 mg) as a white crystalline solid.

#### δ-Lactone of (2*R*,3*S*,3a*S*,4*S*,5*R*)-2-(2-Hydroxyethyl)-4,5-isopropylidenedioxyhexahydropyrrolo­[1,2-*b*]­isoxazol-3-carboxylic Acid (**10**)

The spectroscopic data have been reported previously.^40^

## Supplementary Material





## Data Availability

The data underlying this study are available in the published article and its .

## Notes

Characterization spectra and DFT calculations (PDF)
FAIR data, including the primary NMR FID files, for compounds **1–7** (ZIP)
