# Supplementary material for: Regiodivergence in the Cycloadditions between a Cyclic Nitrone and Carbonyl-Type Dipolarophiles
Source: J Org Chem. 2025 Jul 29;90(31):11020–32. doi: 10.1021/acs.joc.5c00727 (PMC12340946; doi:10.1021/acs.joc.5c00727)
Supplement: Supplementary file 1 [file jo5c00727_si_001.pdf]

Supporting Information for

STUDY OF THE REGIODIVERGENCE IN THE CYCLOADDITIONS BETWEEN A CYCLIC NITRONE AND CARBONYL-TYPE DIPOLAROPHILES

Alberto Esteban, [a] *Carlos T. Nieto,* [a] *Narciso M. Garrido,* [a] *Francisca Sanz,*[b] *and David Díez*[a,]*

[a]Organic chemistry Department, Chemical Sciences Faculty, University of Salamanca. Plaza de los Caídos 1-5, 37008, Salamanca, Spain.

[b]X-Ray diffraction service, Nucleus Platform, University of Salamanca. Plaza de los Caídos 1-5, 37008, Salamanca, Spain.

*Corresponding author's e-mail address: ddm@usal.es. Fax: +34 923 294574.

Table of contents

NMR (^{1}H, ^{13}C, HSQC, HMBC and COSY), IR and HRMS spectra……………..……S3-S32

3D representation of the obtained transition states……………………………...S33-S38

Cartesian coordinates for the optimized geometries………………………………S39-S89

Procedure for the single crystal preparation..S90

X-Ray Crystallography...S90

a. Crystal Data S91-S95

b. Molecular structures of the title compounds..S96-S99

References……………………………………………………………………...S100

NMR (^{1}H, ^{13}C{^{1}H}, HSQC, HMBC and COSY), IR and HRMS spectra

^{1}H NMR (400MHz, $CDCl_3$) and ^{13}C{^{1}H} NMR (100MHz, $CDCl_3$) spectra of **2**

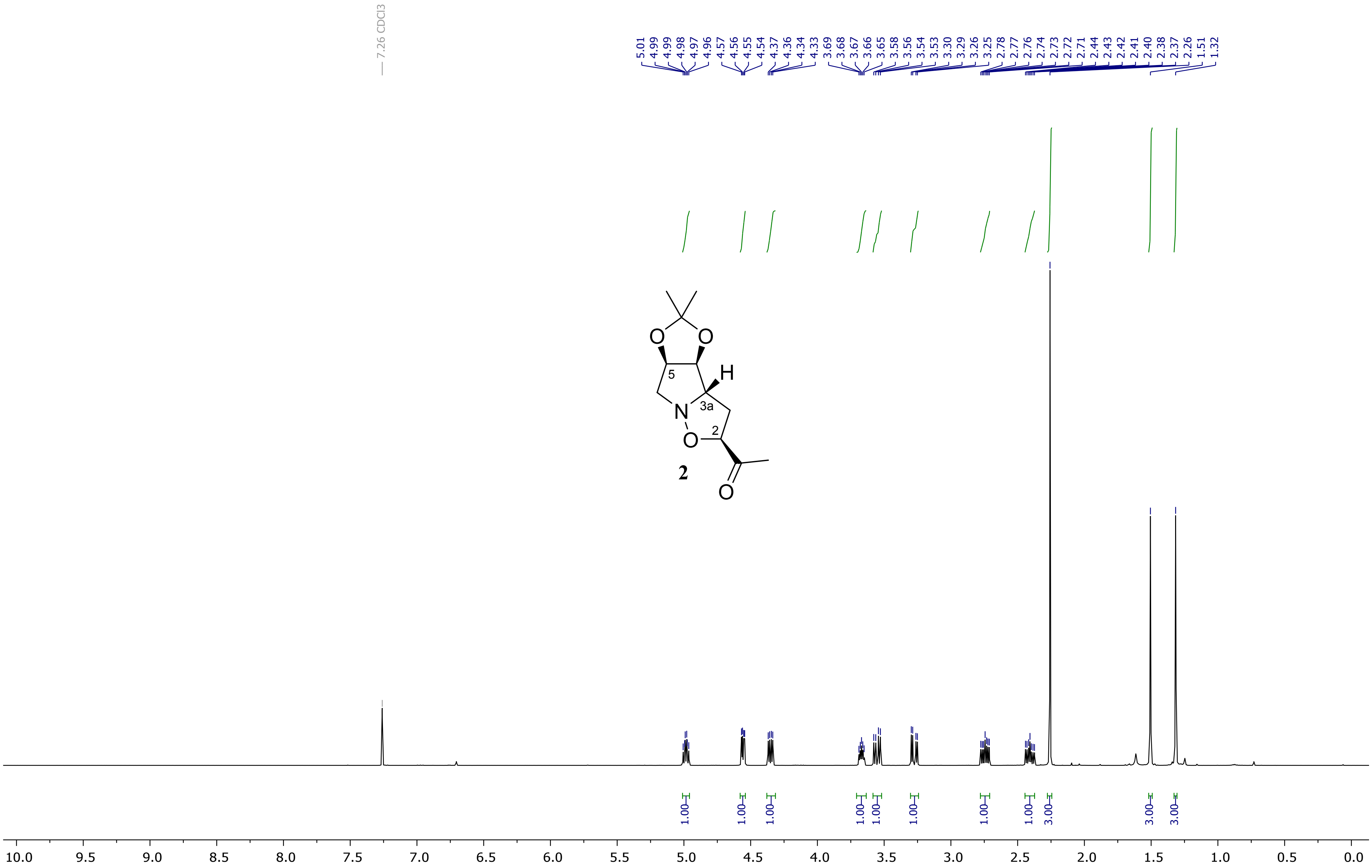

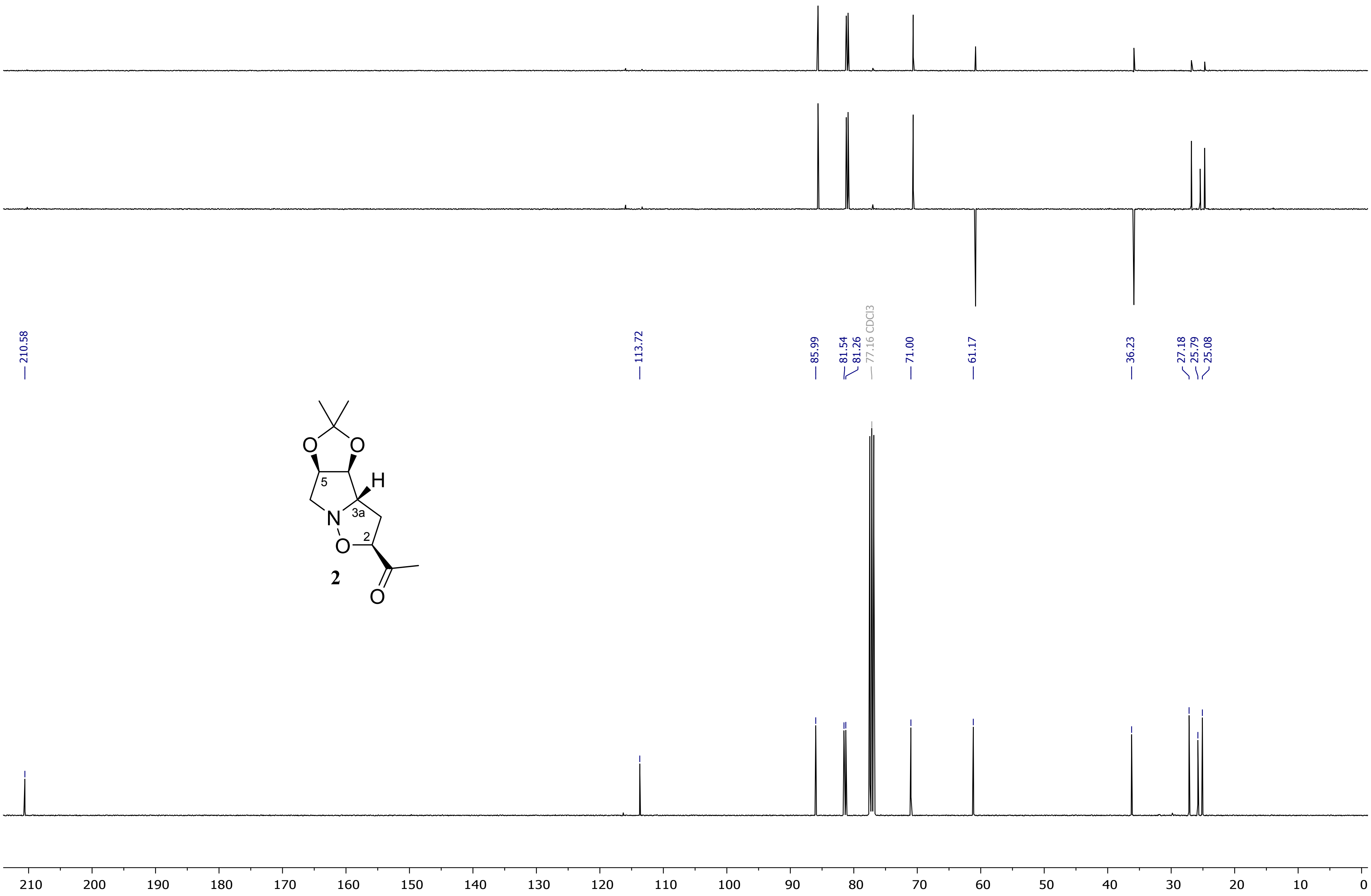

210.58
113.72
85.99
81.54
81.26
77.16 CDCl3
71.00
61.17
36.23
27.18
25.79
25.08
O
O
H
5
N
3a
O
2
O
2
210 200 190 180 170 160 150 140 130 120 110 100 90 80 70 60 50 40 30 20 10 0

2D NMR spectra HSQC, HMBC and COSY of **2**

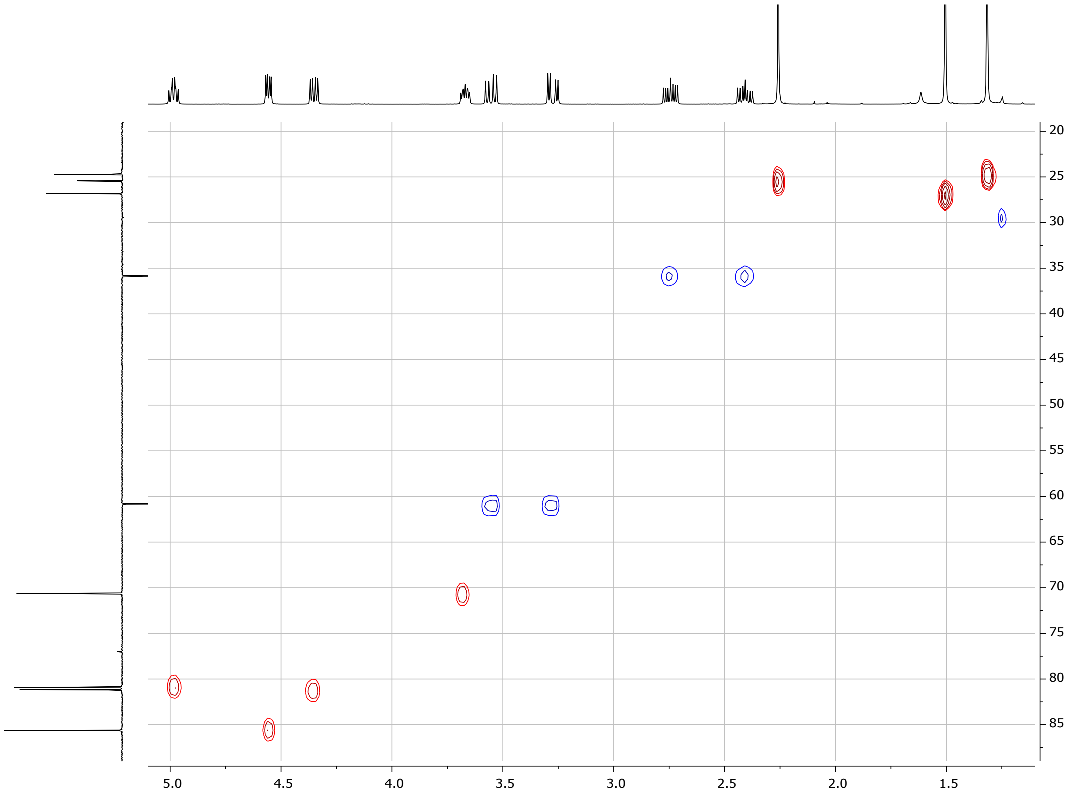

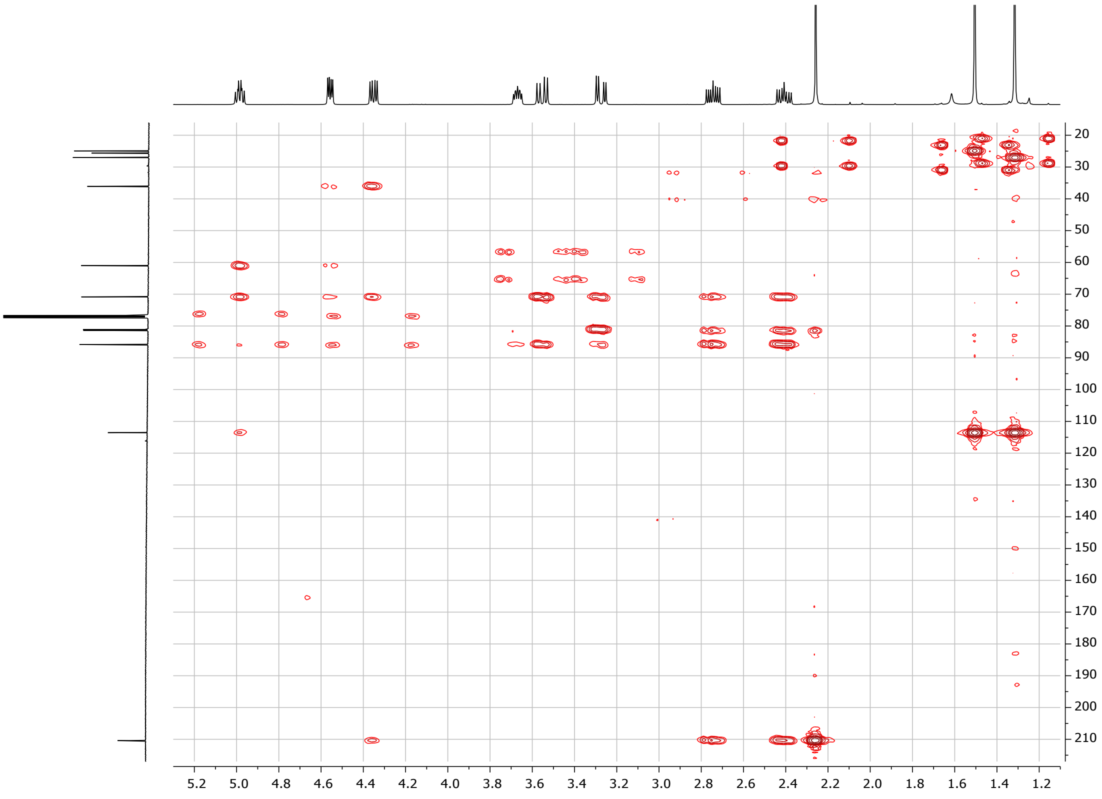

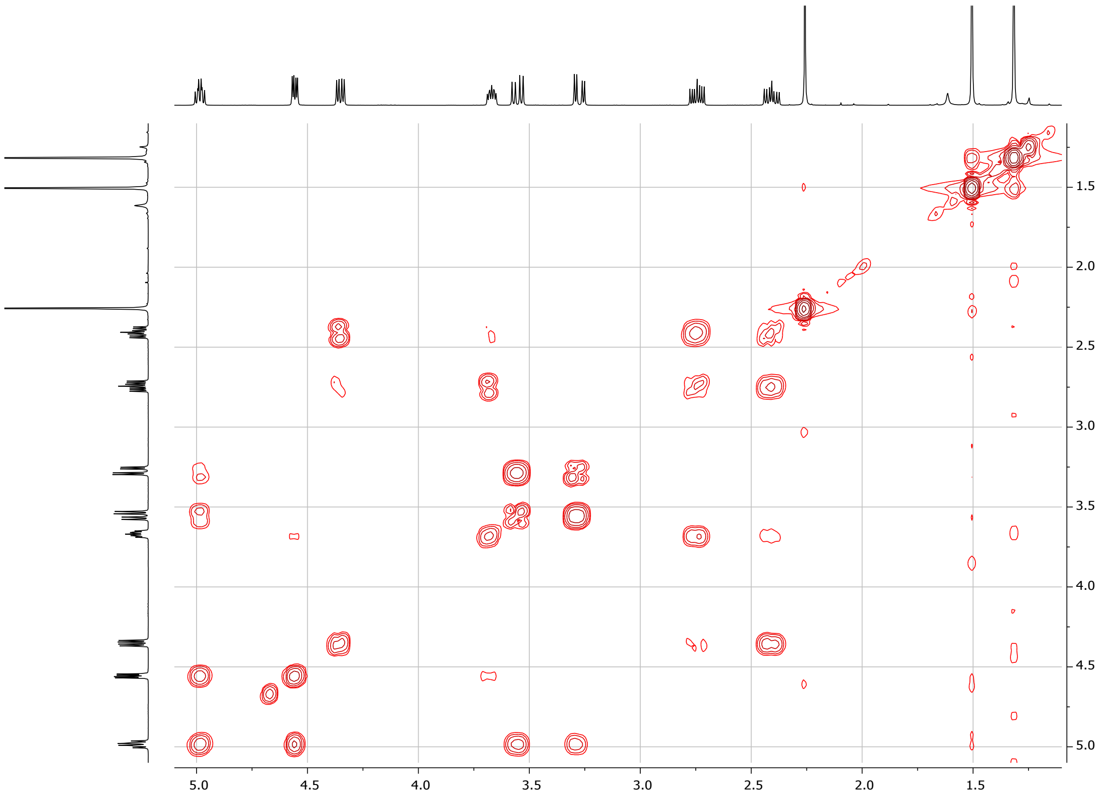

IR spectra of **2**

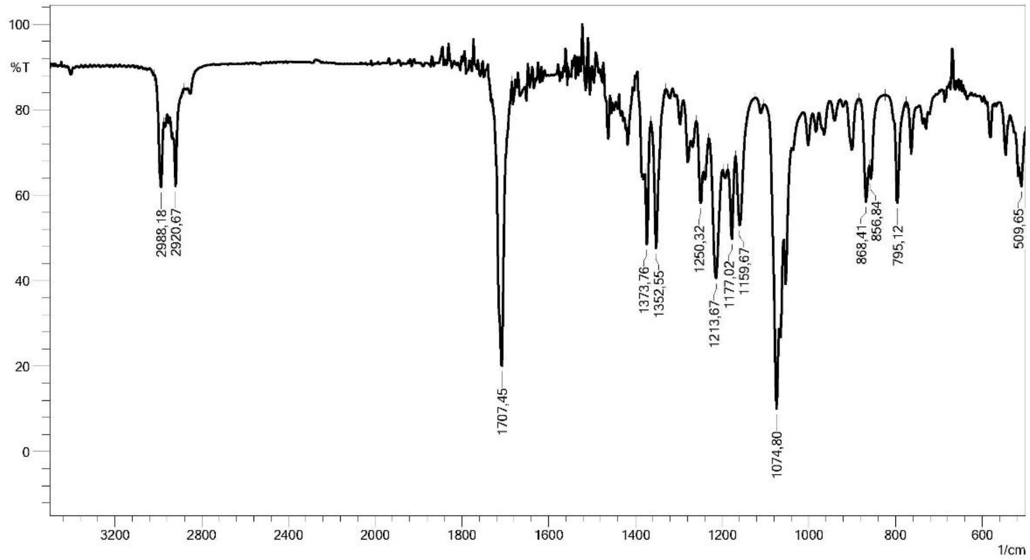

HRMS spectra of **2**

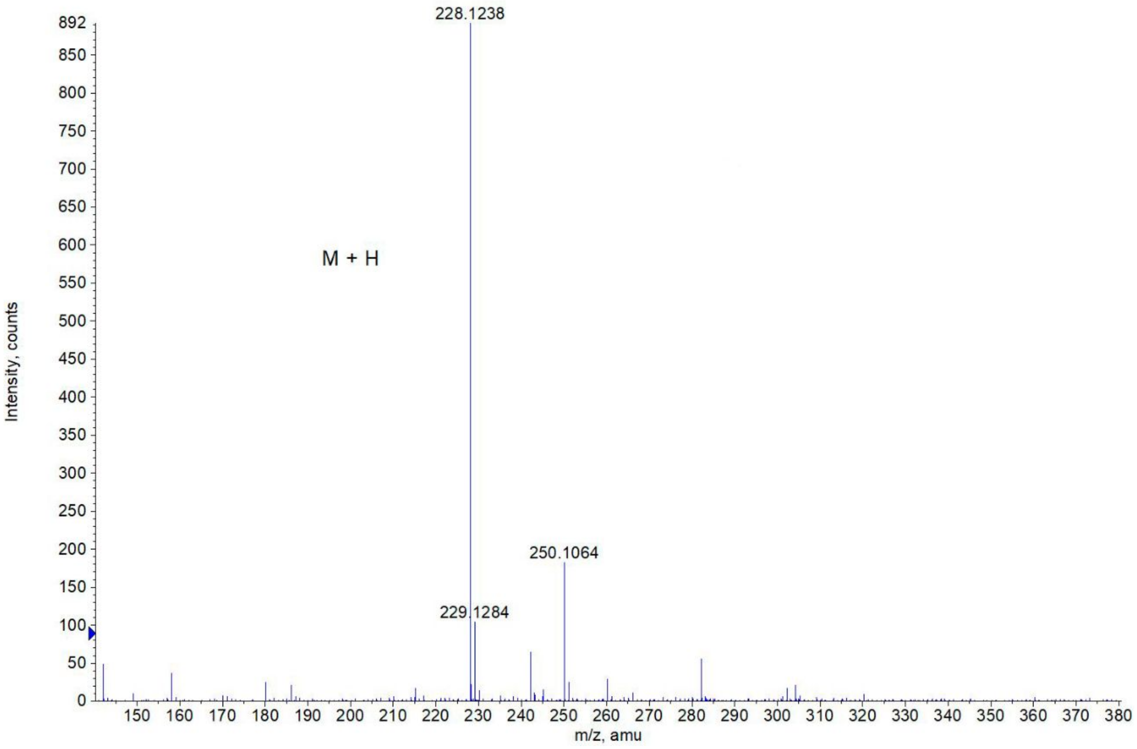

^{1}H NMR (400MHz, $CDCl_3$) and $^{13}C\{^{1}H\}$ NMR (100MHz, $CDCl_3$) spectra of **3**

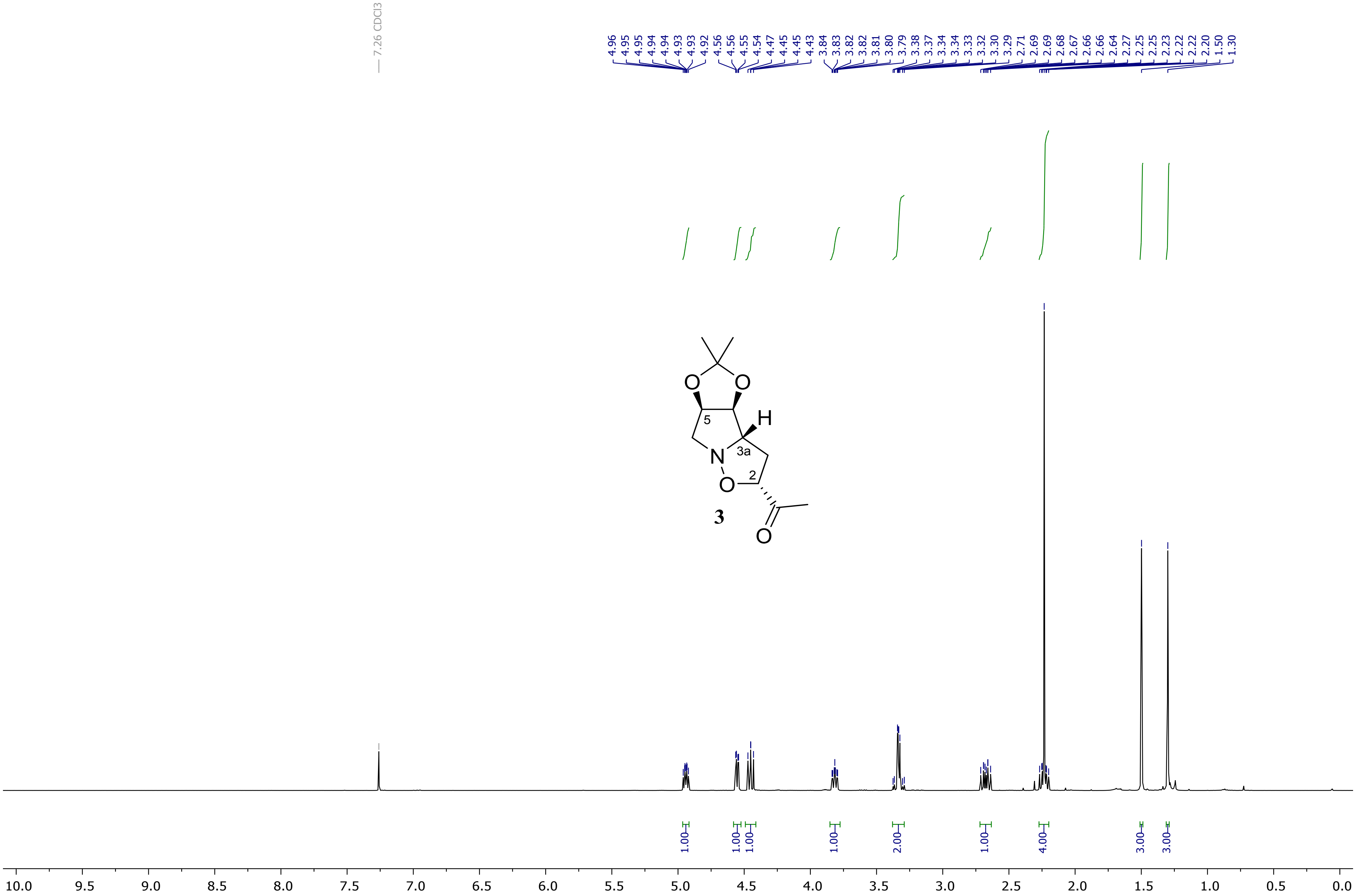

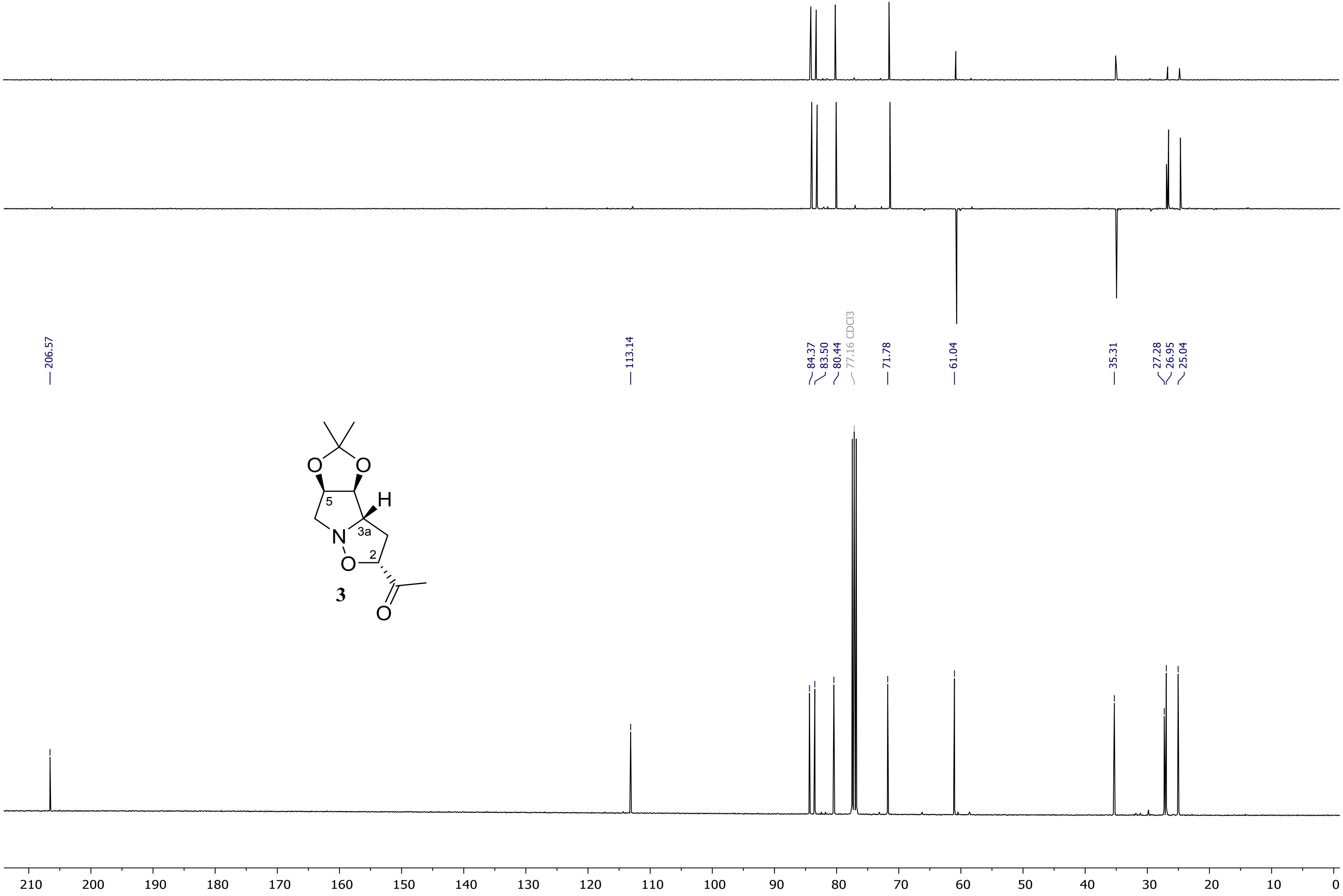
206.57
113.14
84.37
83.50
80.44
77.16 CDCl3
71.78
61.04
35.31
27.28
26.95
25.04
O
O
H
5
N
3a
O
2
O
3
210 200 190 180 170 160 150 140 130 120 110 100 90 80 70 60 50 40 30 20 10 0

2D NMR spectra HSQC, HMBC and COSY of **3**

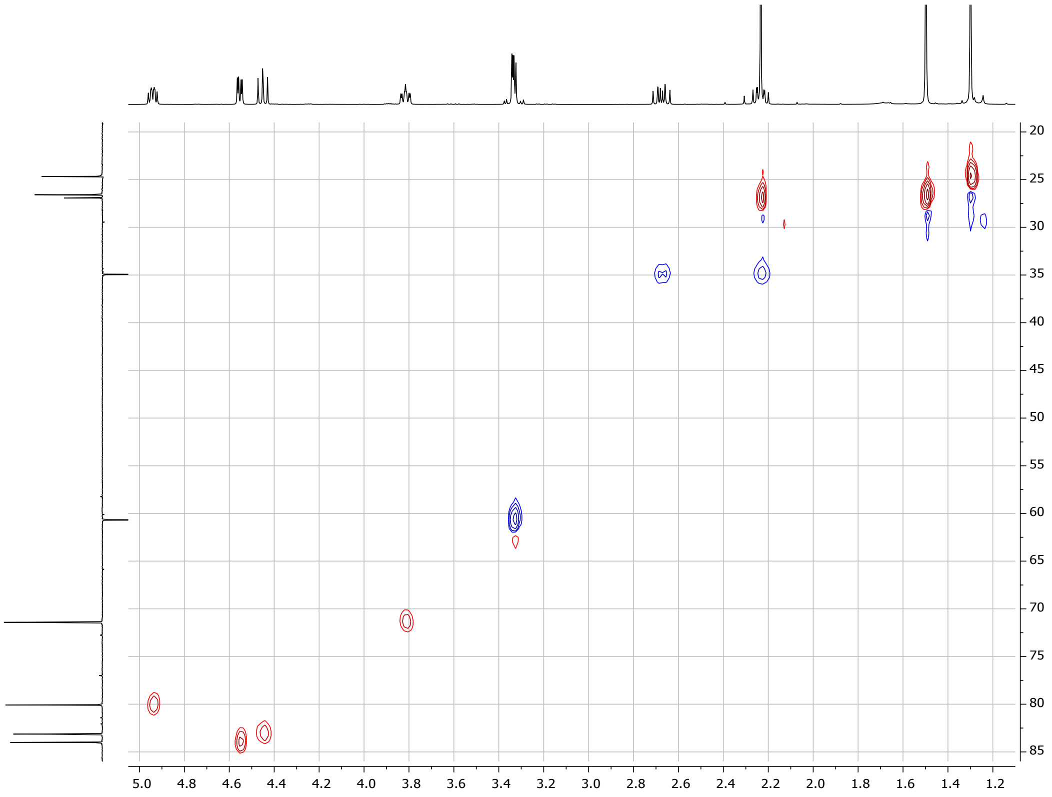

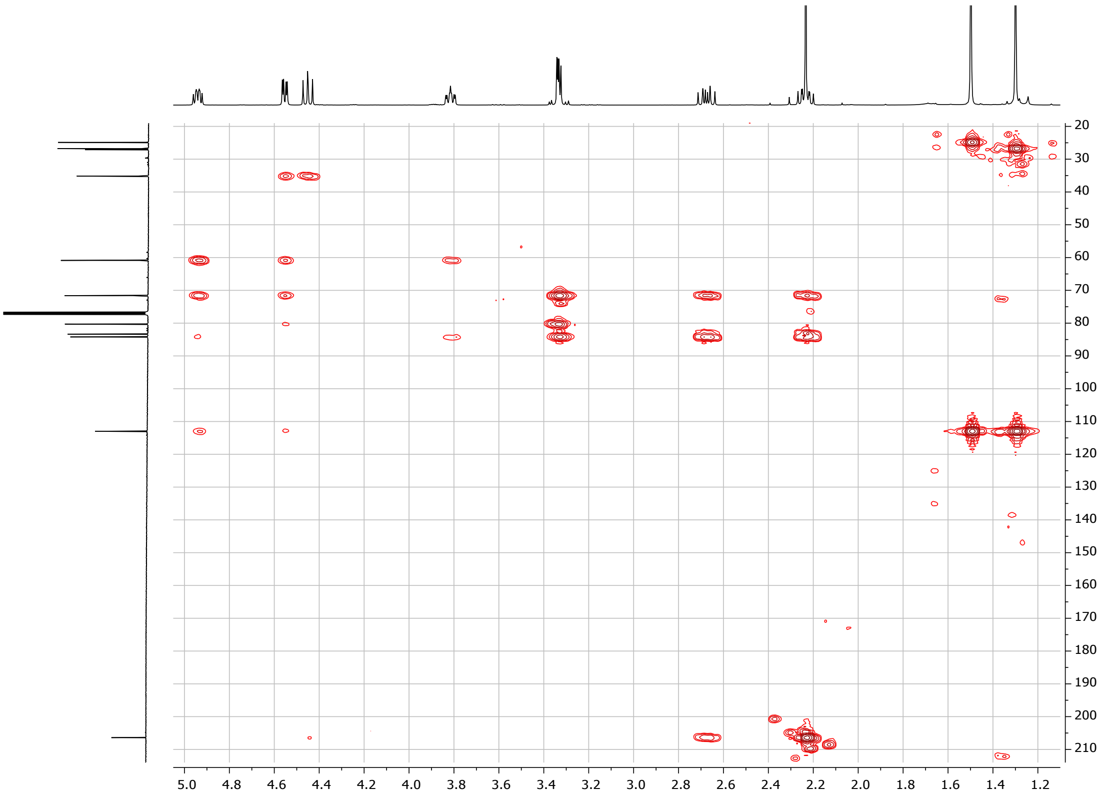

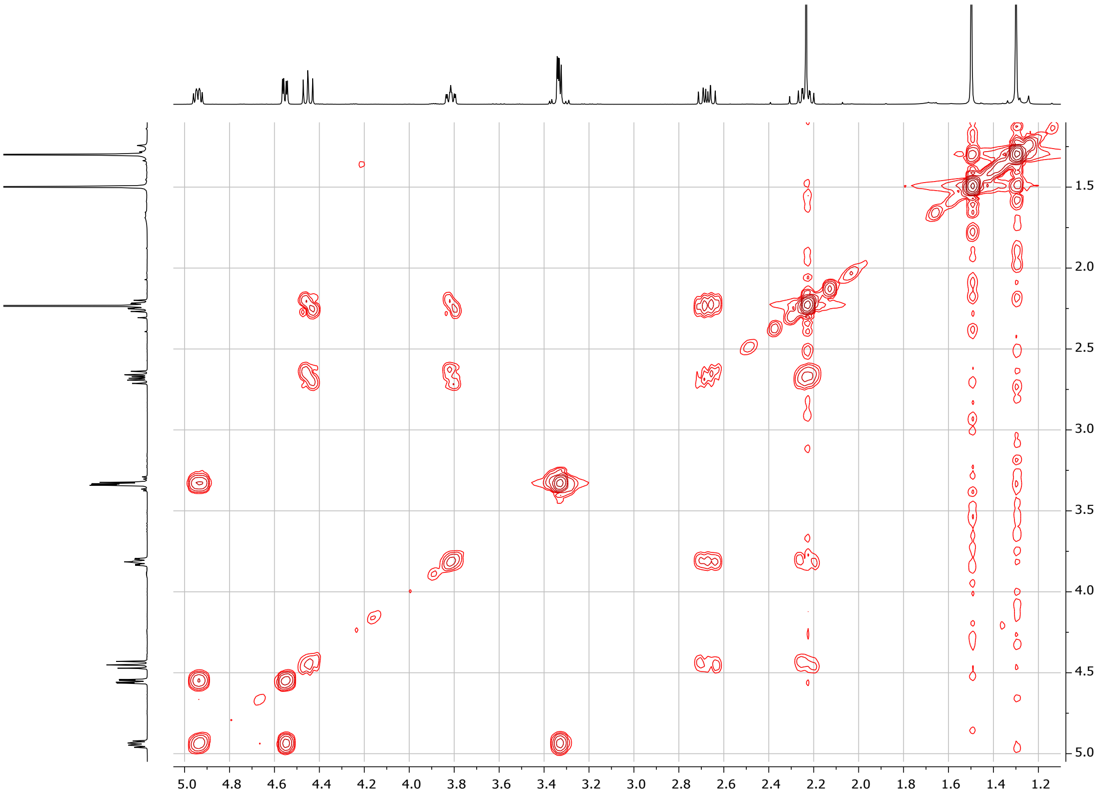

IR spectra of **3**

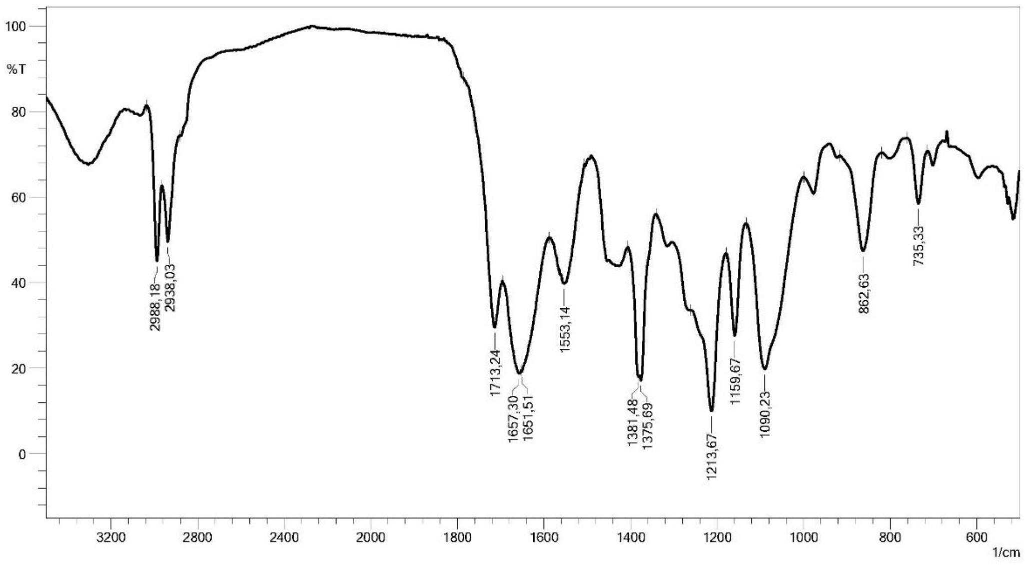

HRMS spectra of **3**

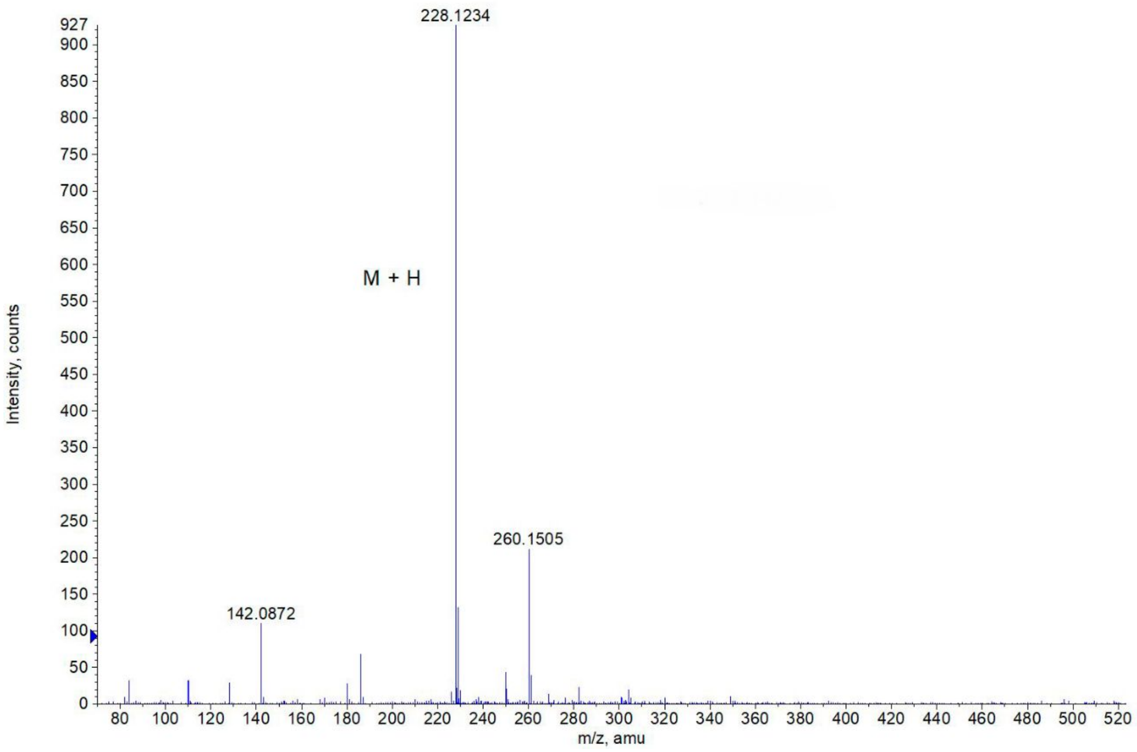

^{1}H NMR (400MHz, $CDCl_3$) and $^{13}C\{^{1}H\}$ NMR (100MHz, $CDCl_3$) spectra of **4**

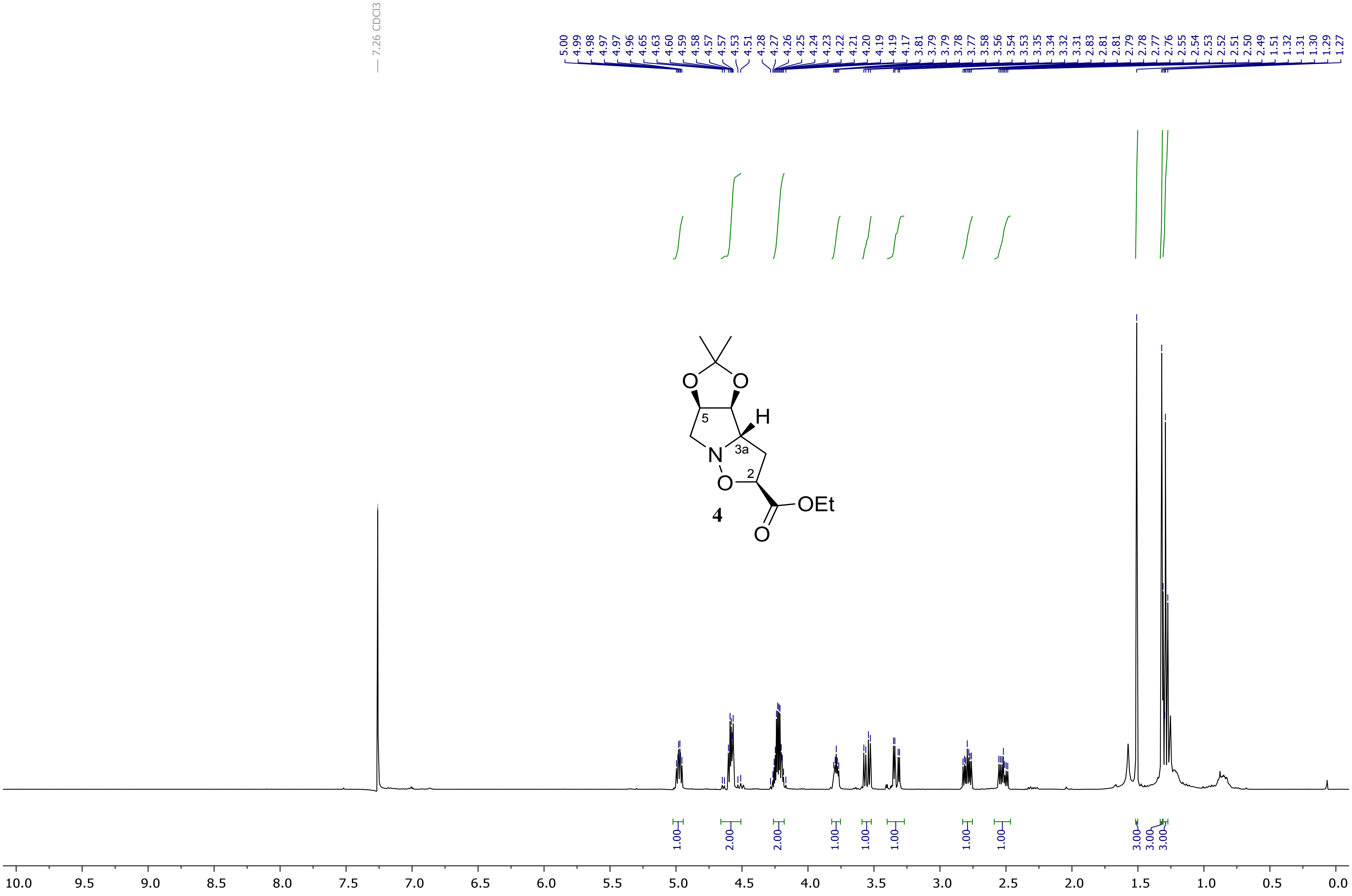

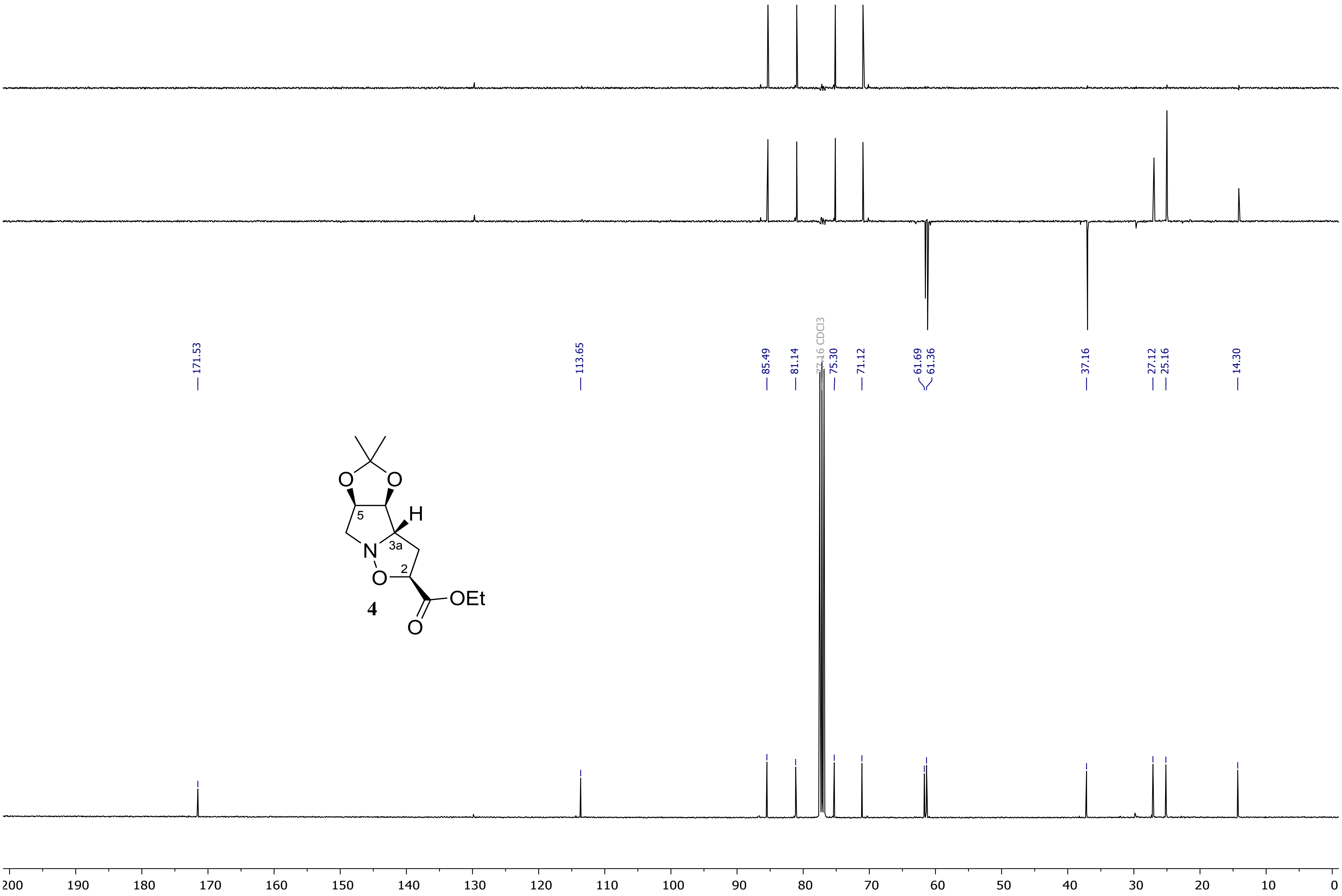

171.53
113.65
85.49
81.14
77.16 CDCl3
75.30
71.12
61.69
61.36
37.16
27.12
25.16
14.30
O
O
H
5
N
3a
2
O
OEt
O
200
190
180
170
160
150
140
130
120
110
100
90
80
70
60
50
40
30
20
10
0

4

2D NMR spectra HSQC, HMBC and COSY of **4**

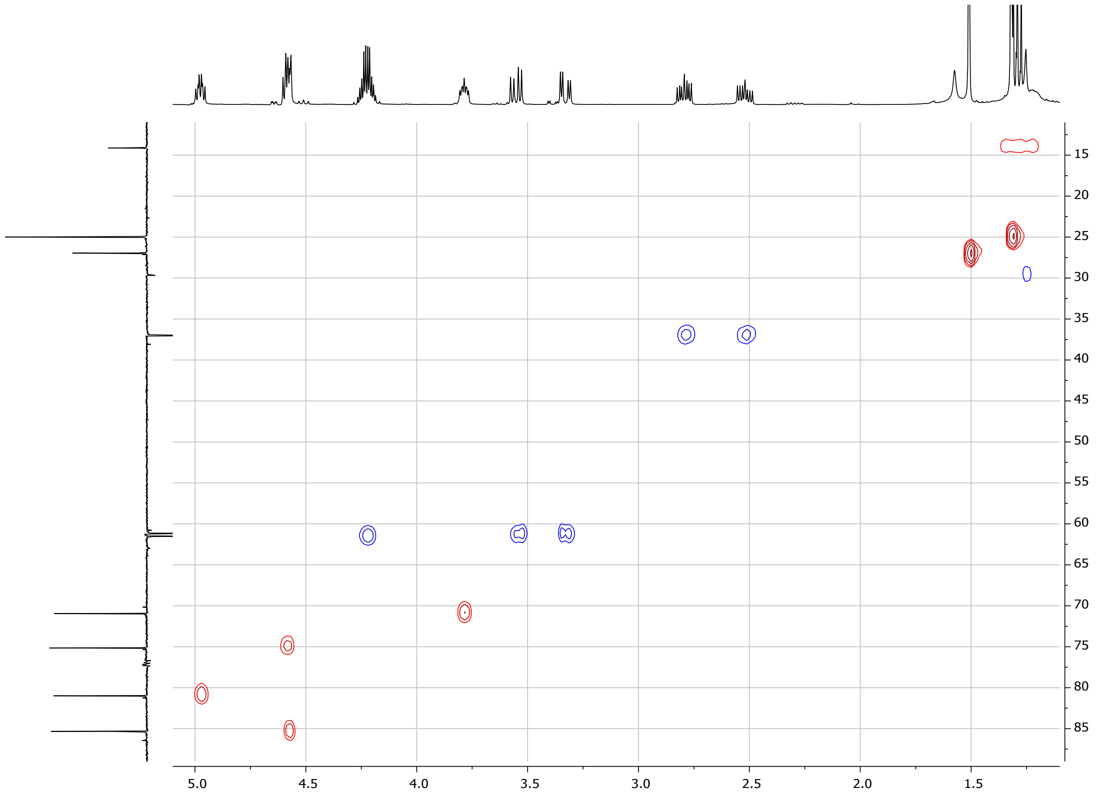

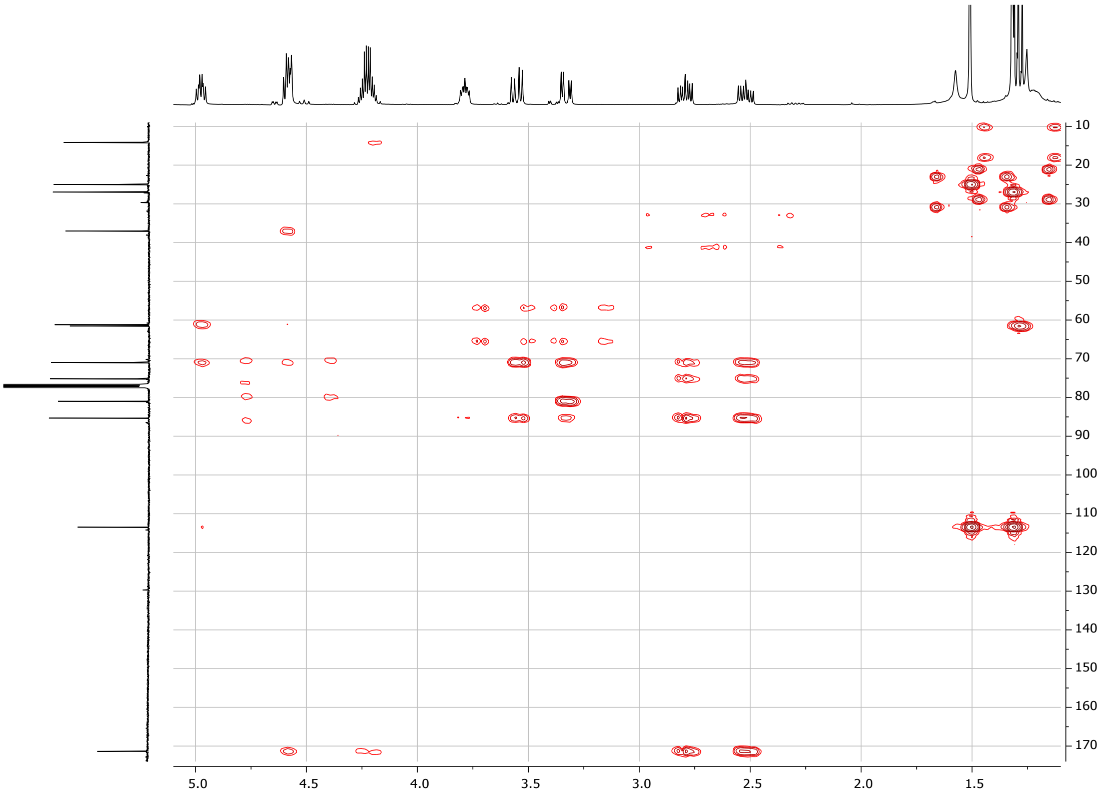

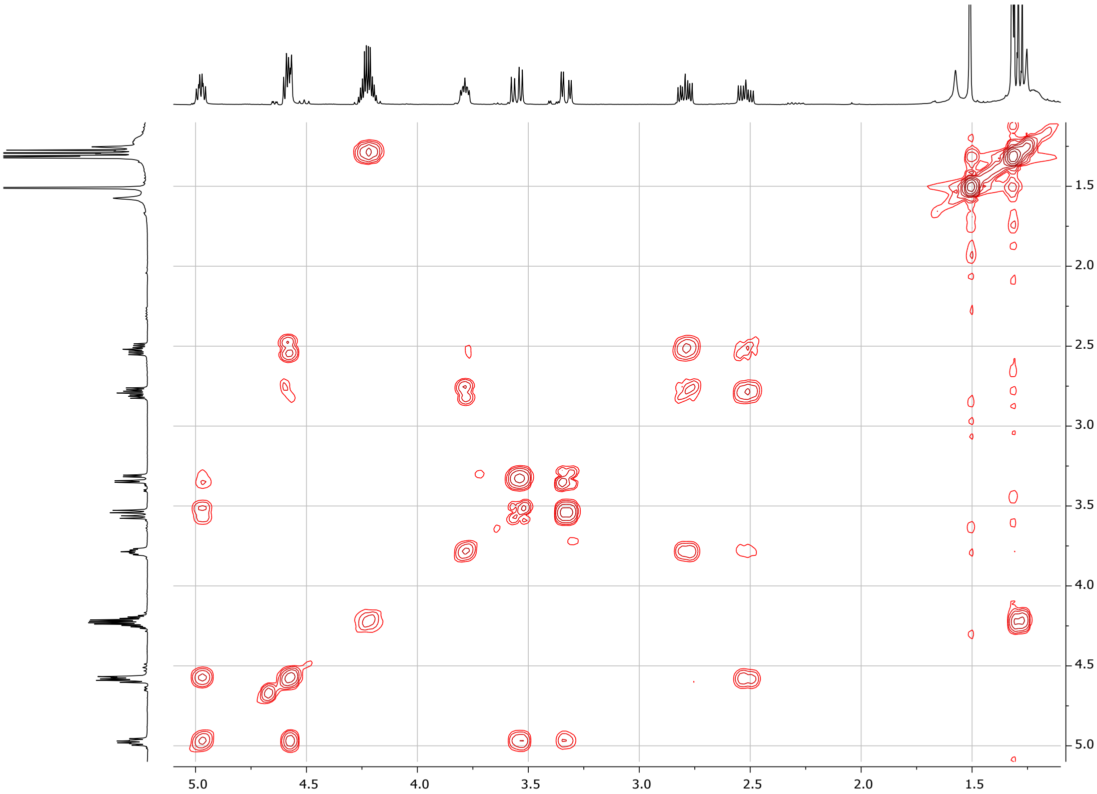

IR spectra of **4**

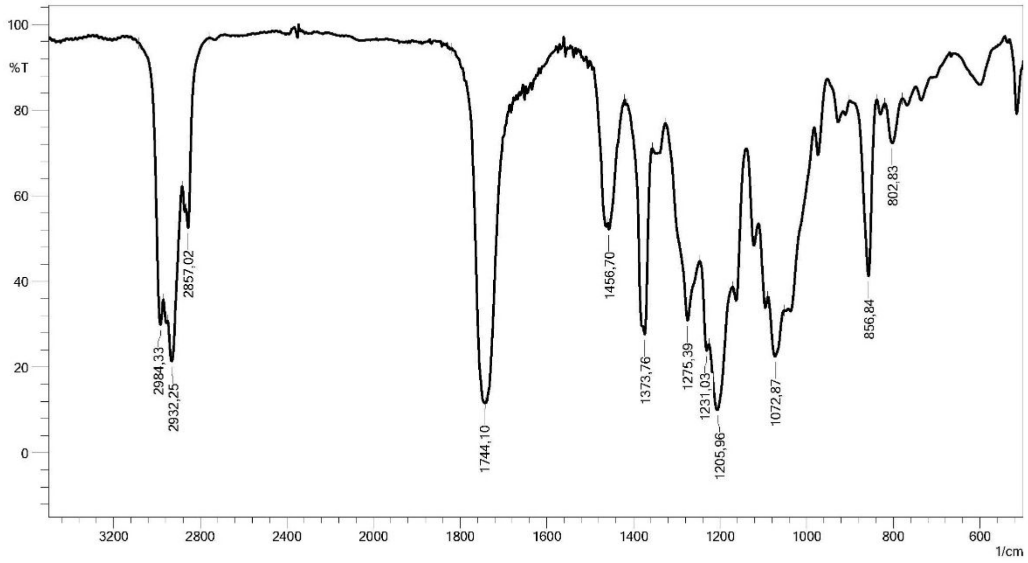

HRMS spectra of **4**

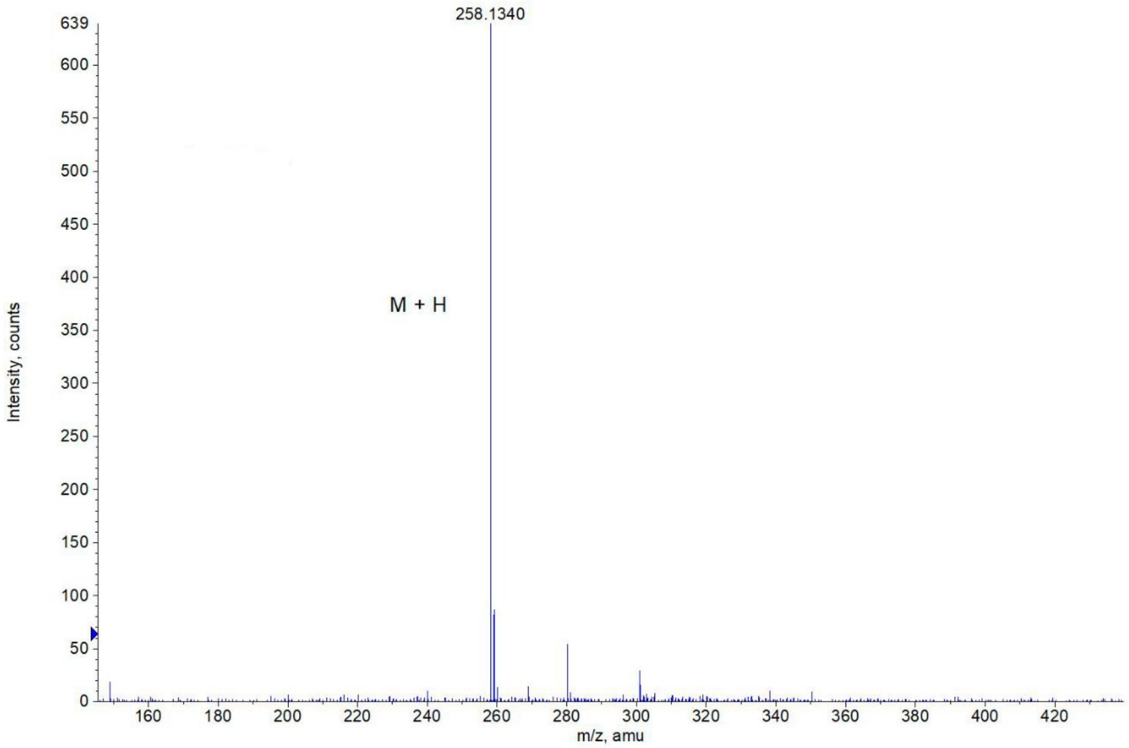

^{1}H NMR (400MHz, $CDCl_3$) and $^{13}C\{^{1}H\}$ NMR (100MHz, $CDCl_3$) spectra of **5**

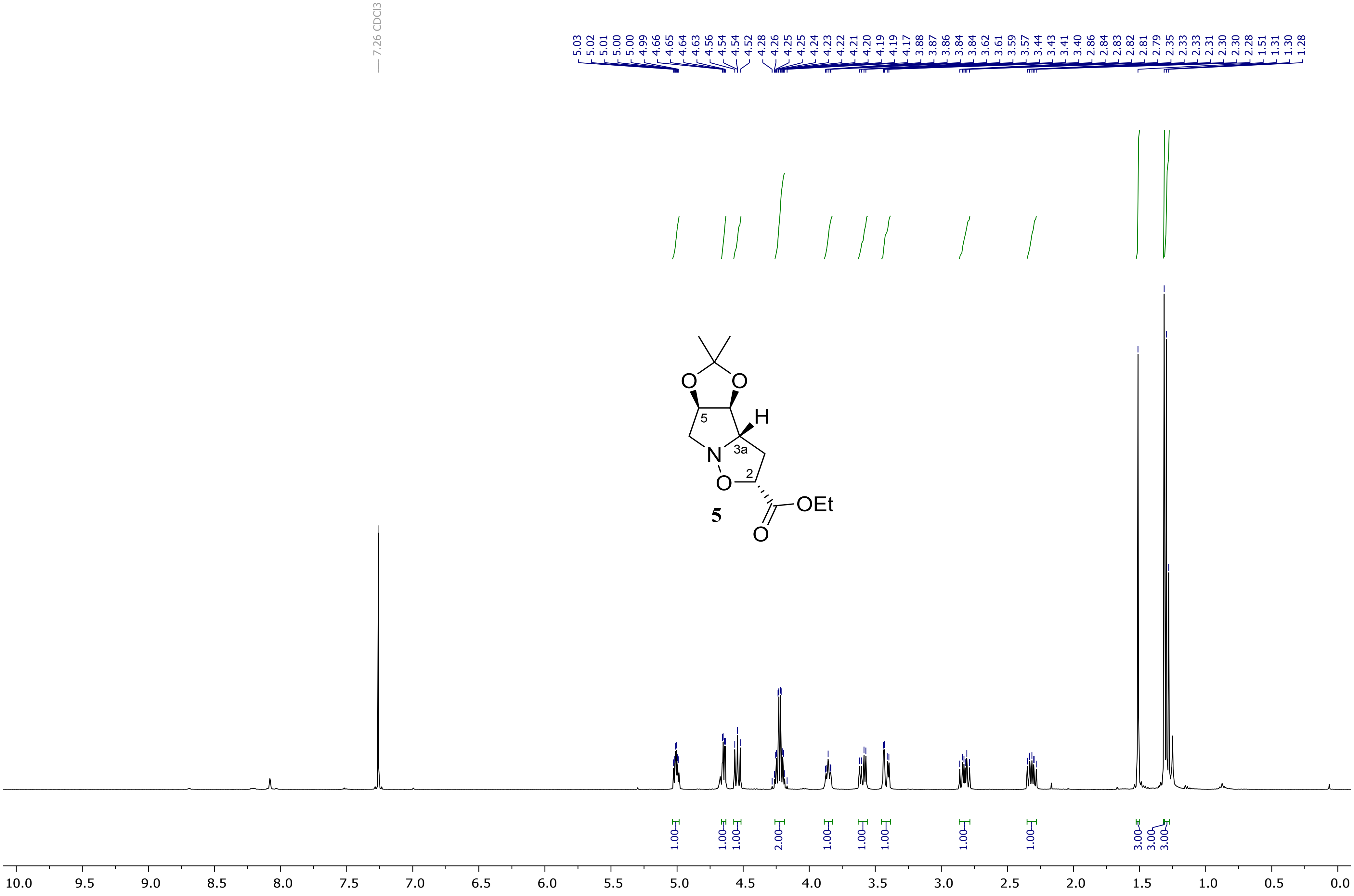

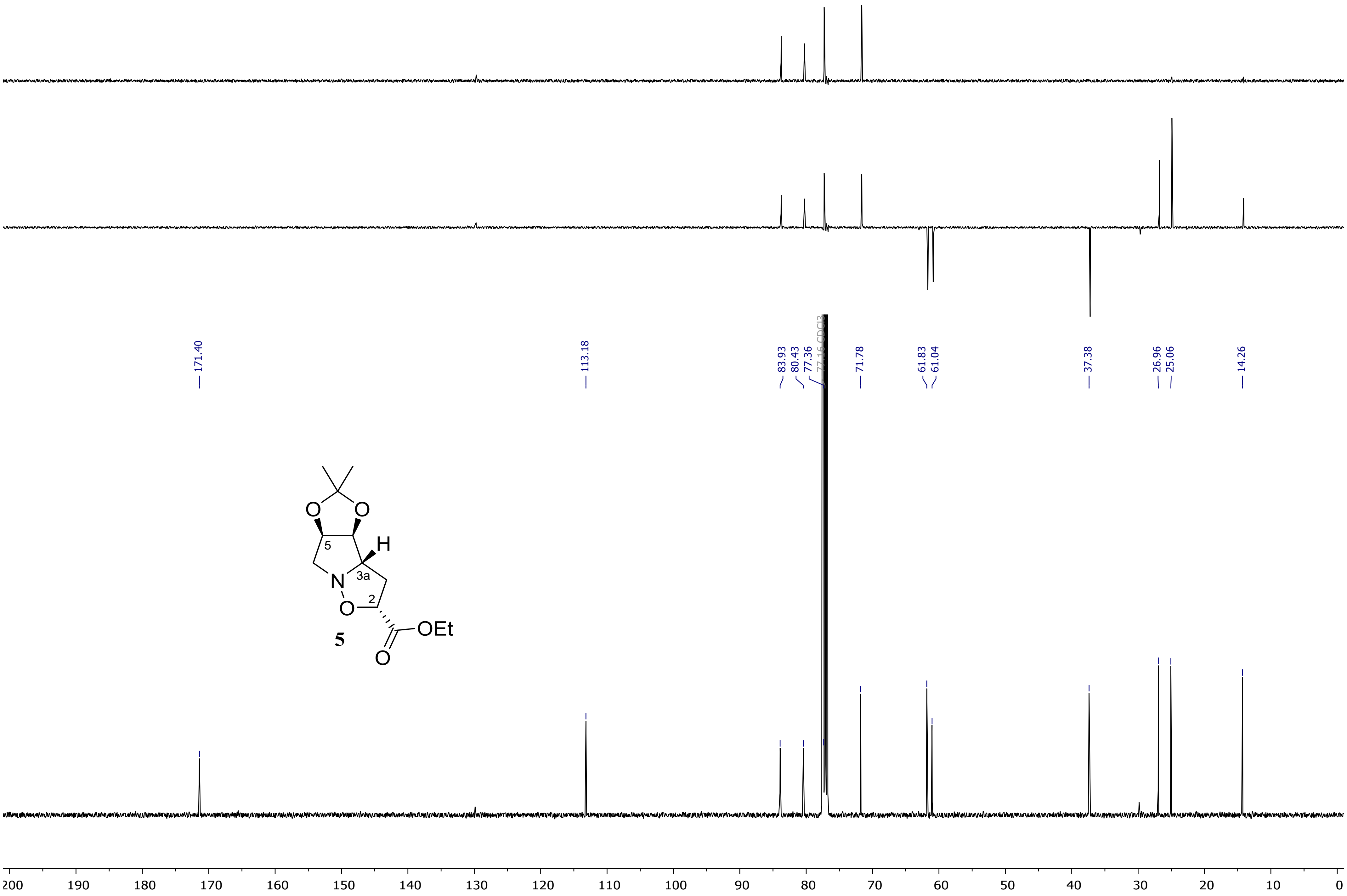
171.40
113.18
83.93
80.43
77.36
77.16 CDCl3
71.78
61.83
61.04
37.38
26.96
25.06
14.26
O
O
5
H
N
3a
2
O
OEt
O
5
200 190 180 170 160 150 140 130 120 110 100 90 80 70 60 50 40 30 20 10 0

2D NMR spectra HSQC, HMBC and COSY of **5**

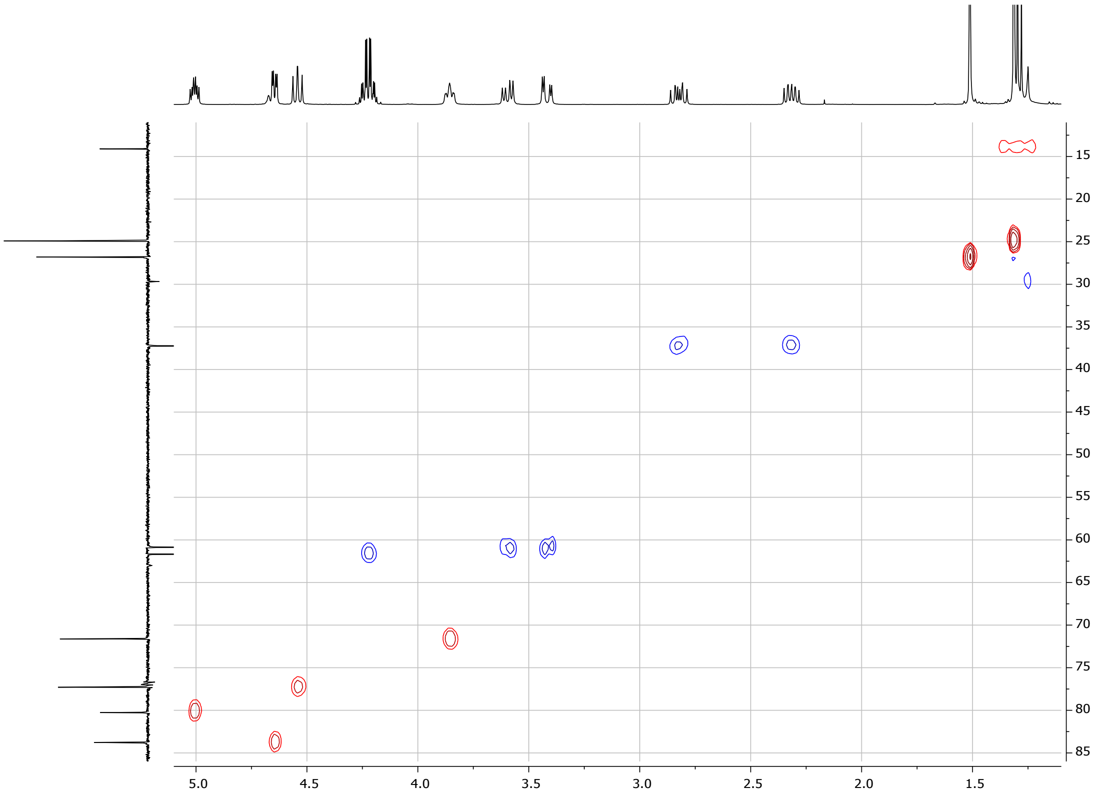

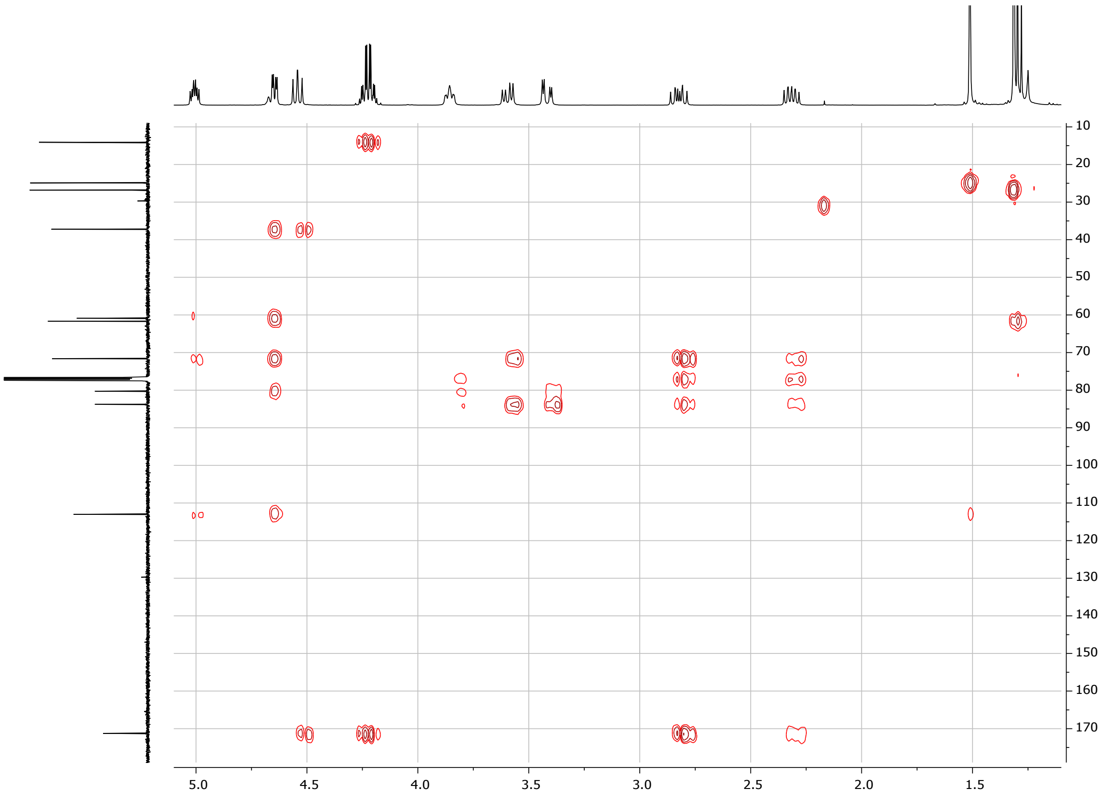

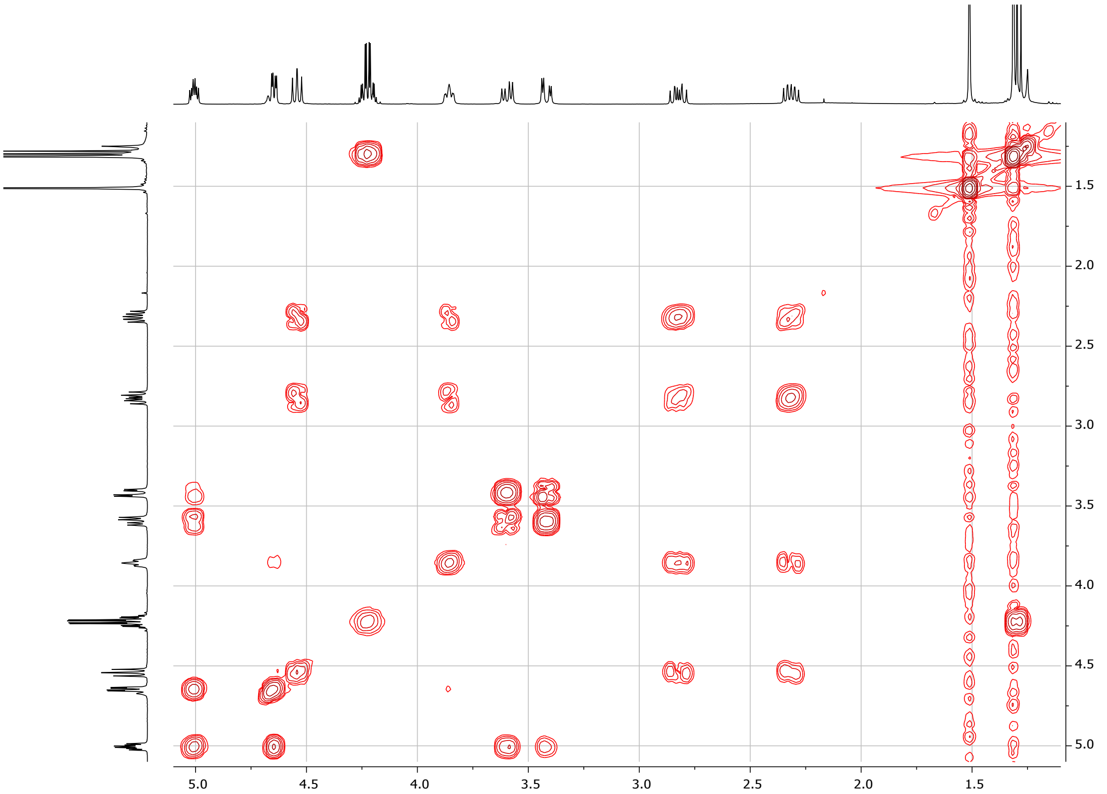

IR spectra of **5**

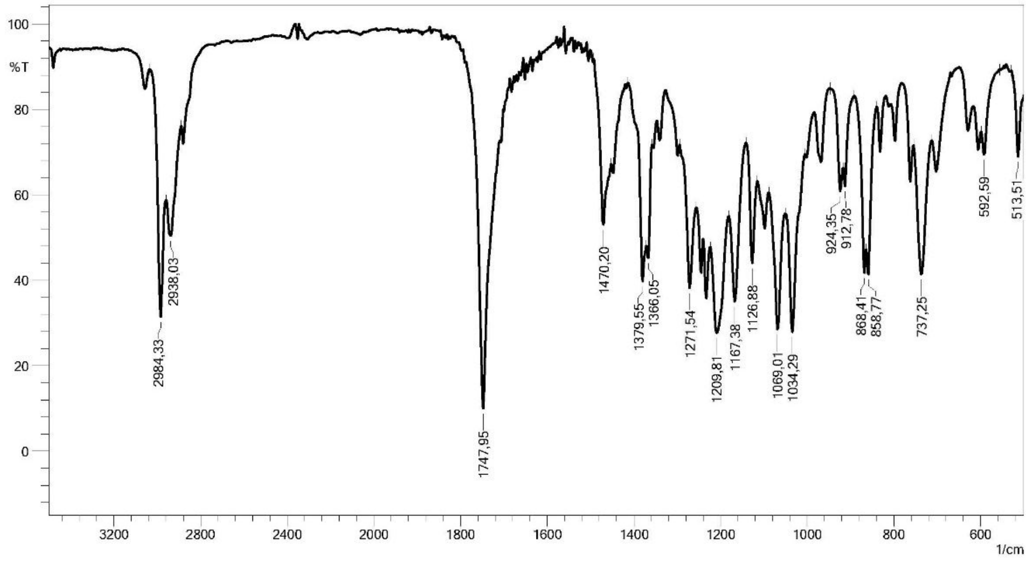

HRMS spectra of **5**

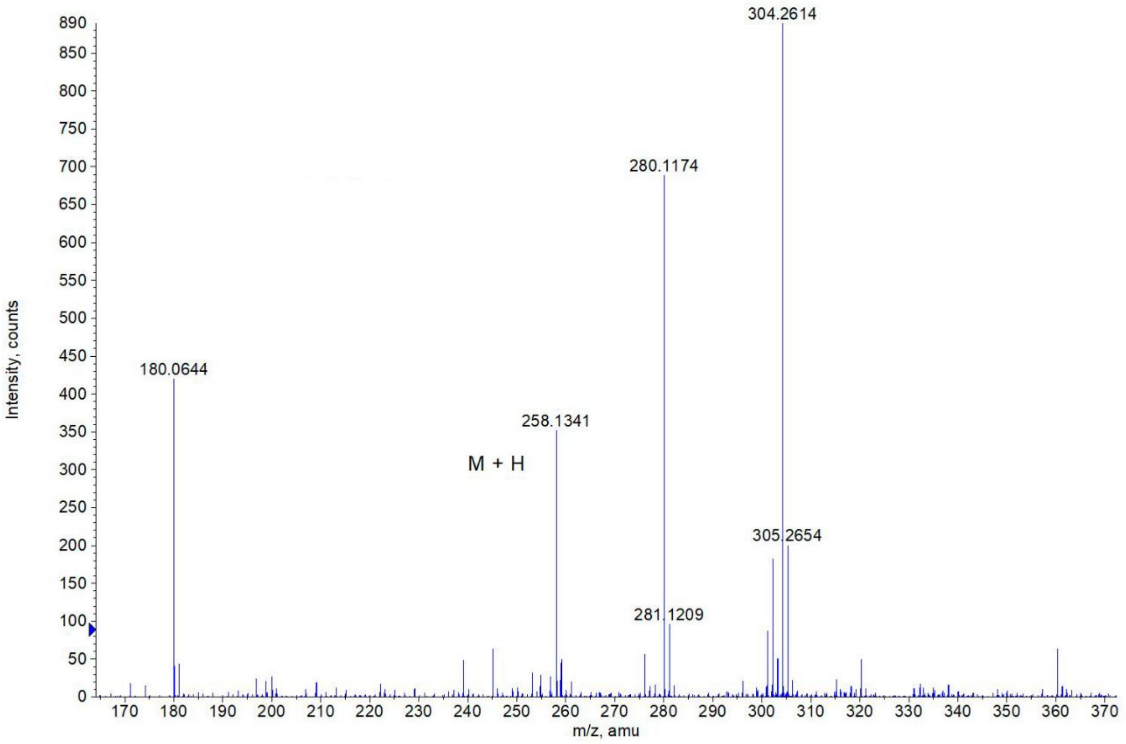

^1H NMR (400MHz, $CDCl_3$) and $^{13}C\{^1H\}$ NMR (100MHz, $CDCl_3$) spectra of **6**

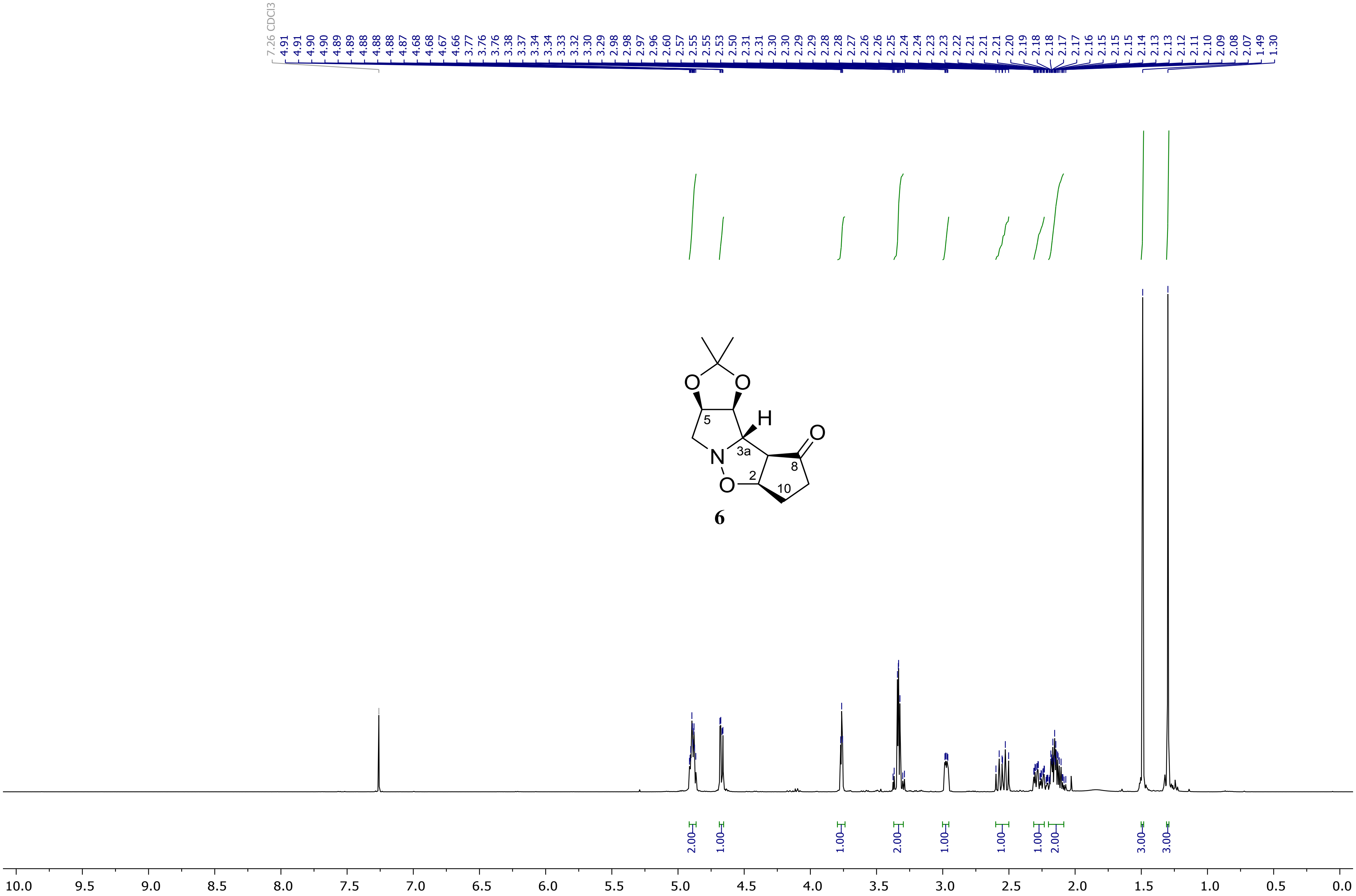

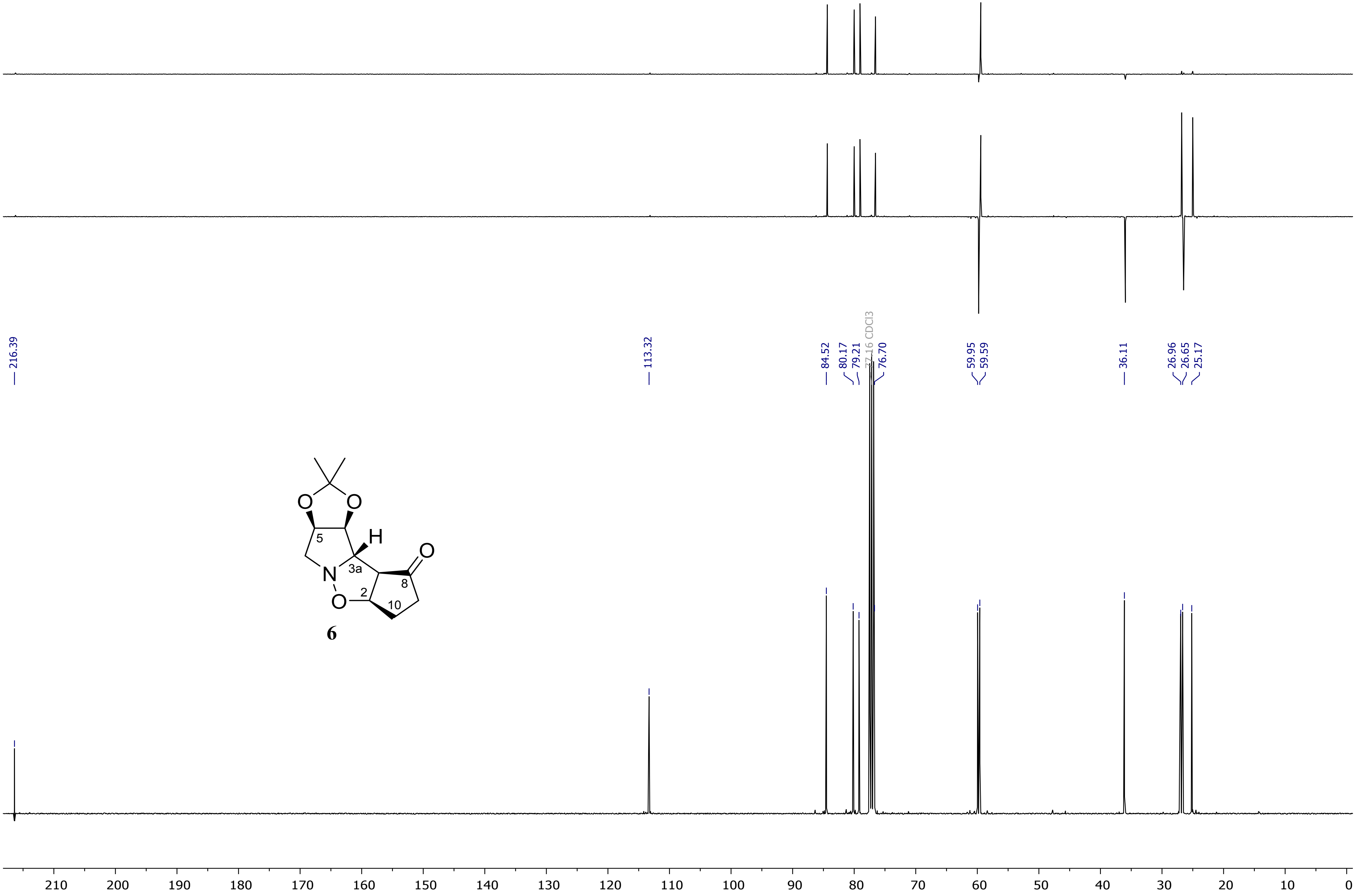
216.39
113.32
84.52
80.17
79.21
77.16 CDCl3
76.70
59.95
59.59
36.11
26.96
26.65
25.17
O
O
H
O
5
N
3a
8
O
2
10
6
210
200
190
180
170
160
150
140
130
120
110
100
90
80
70
60
50
40
30
20
10
0

2D NMR spectra HSQC, HMBC and COSY of **6**

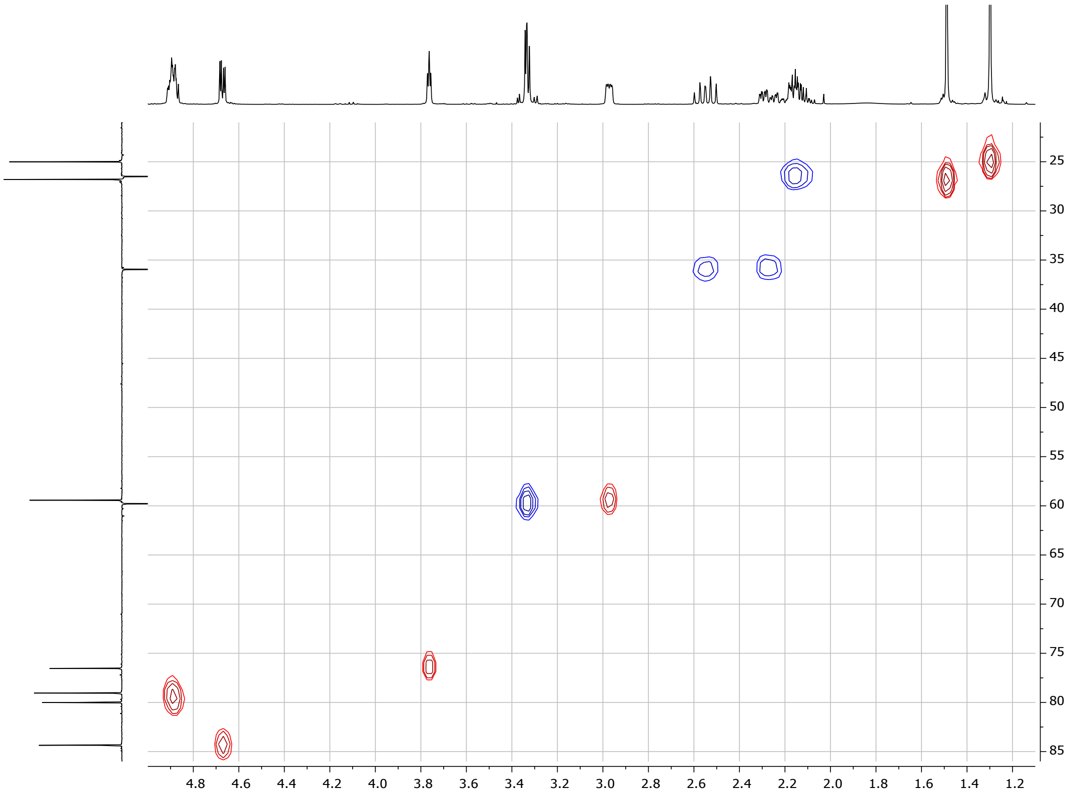

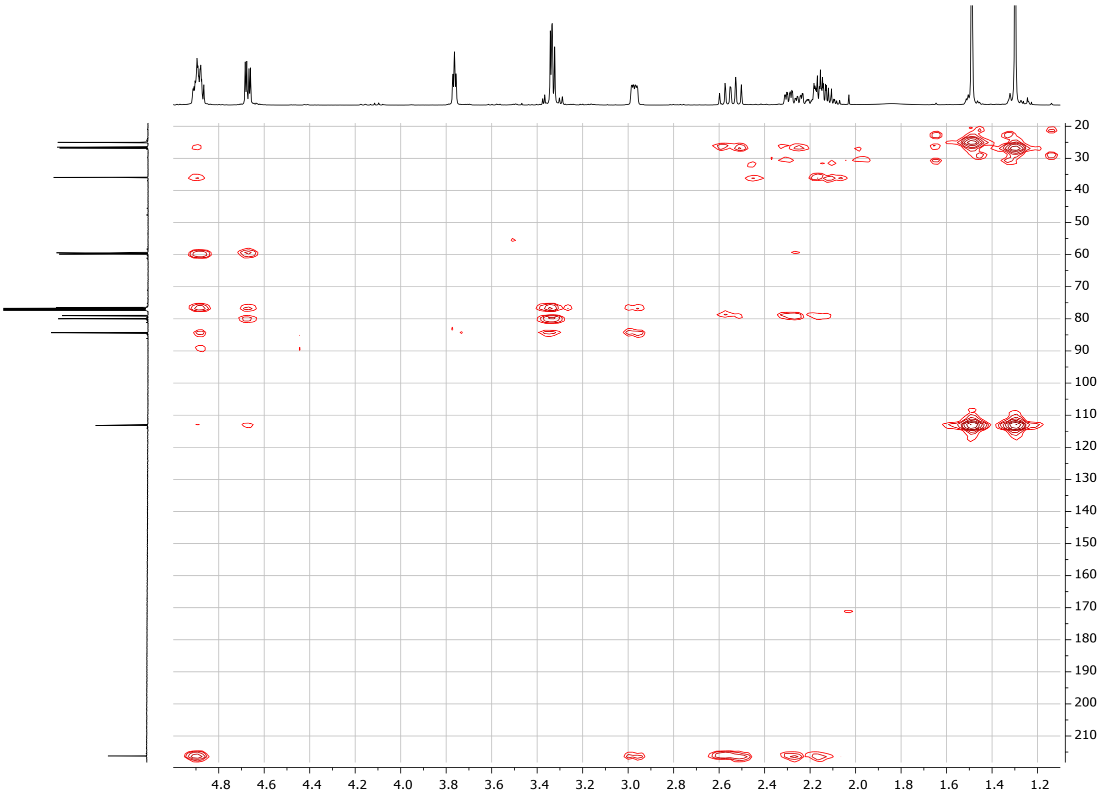

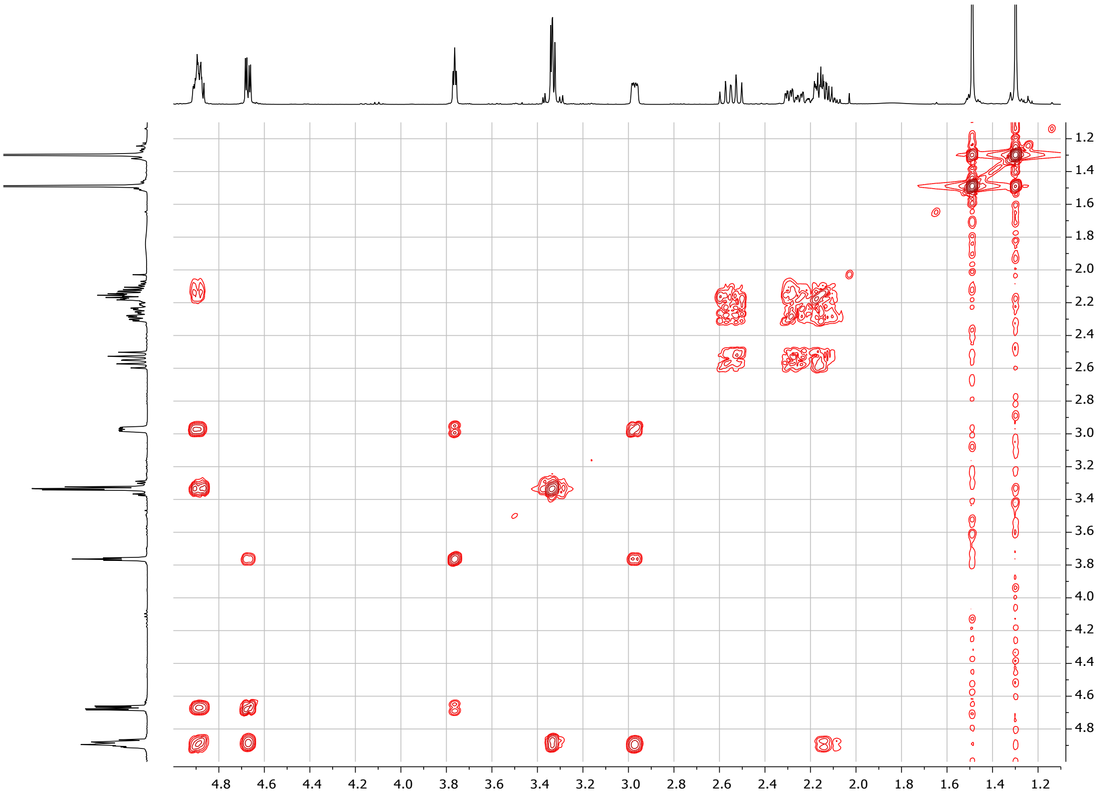

IR spectra of **6**

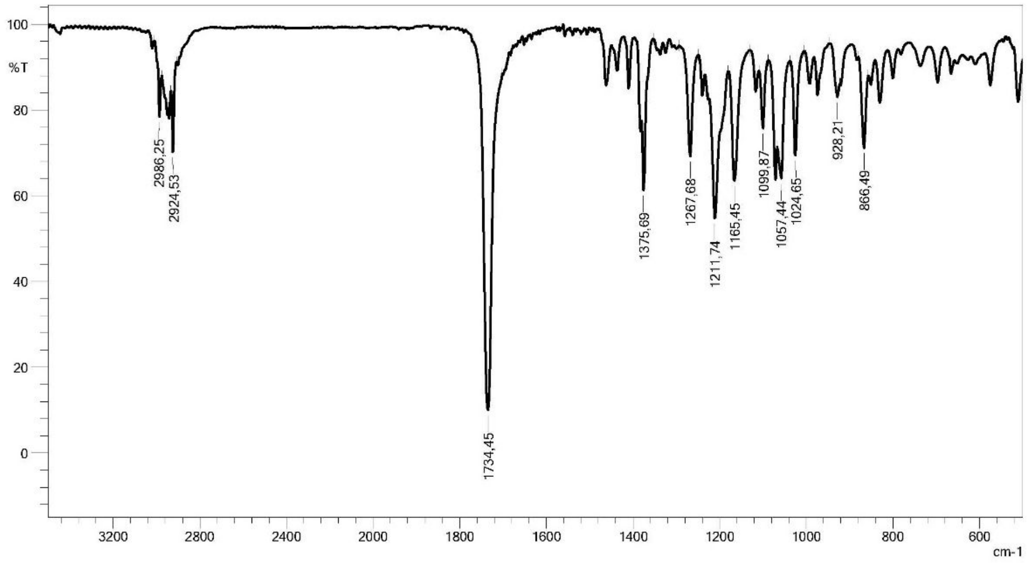

HRMS spectra of **6**

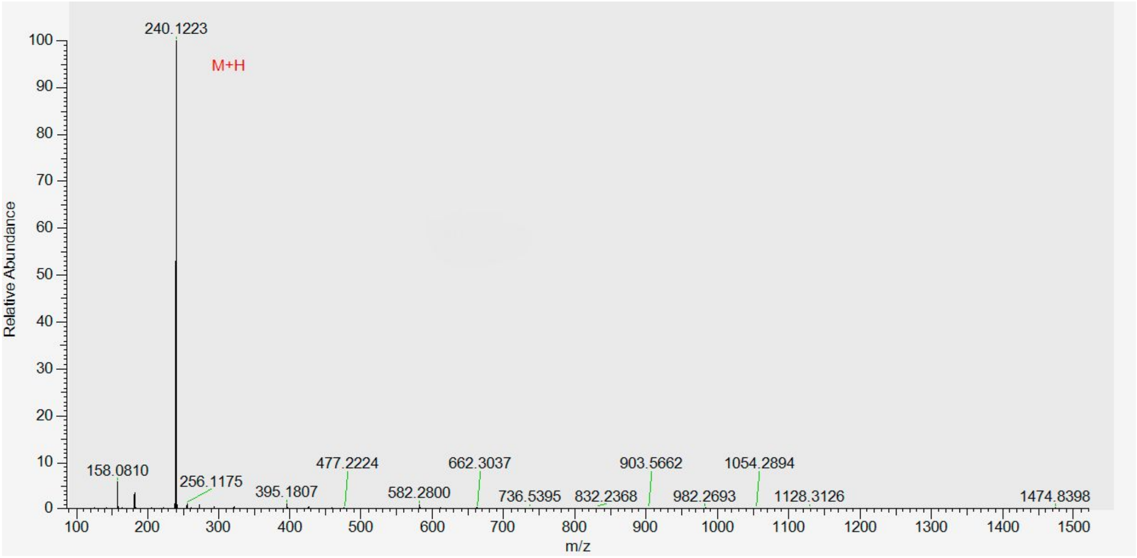

^{1}H NMR (400MHz, $CDCl_3$) and $^{13}C\{^{1}H\}$ NMR (100MHz, $CDCl_3$) spectra of **7**

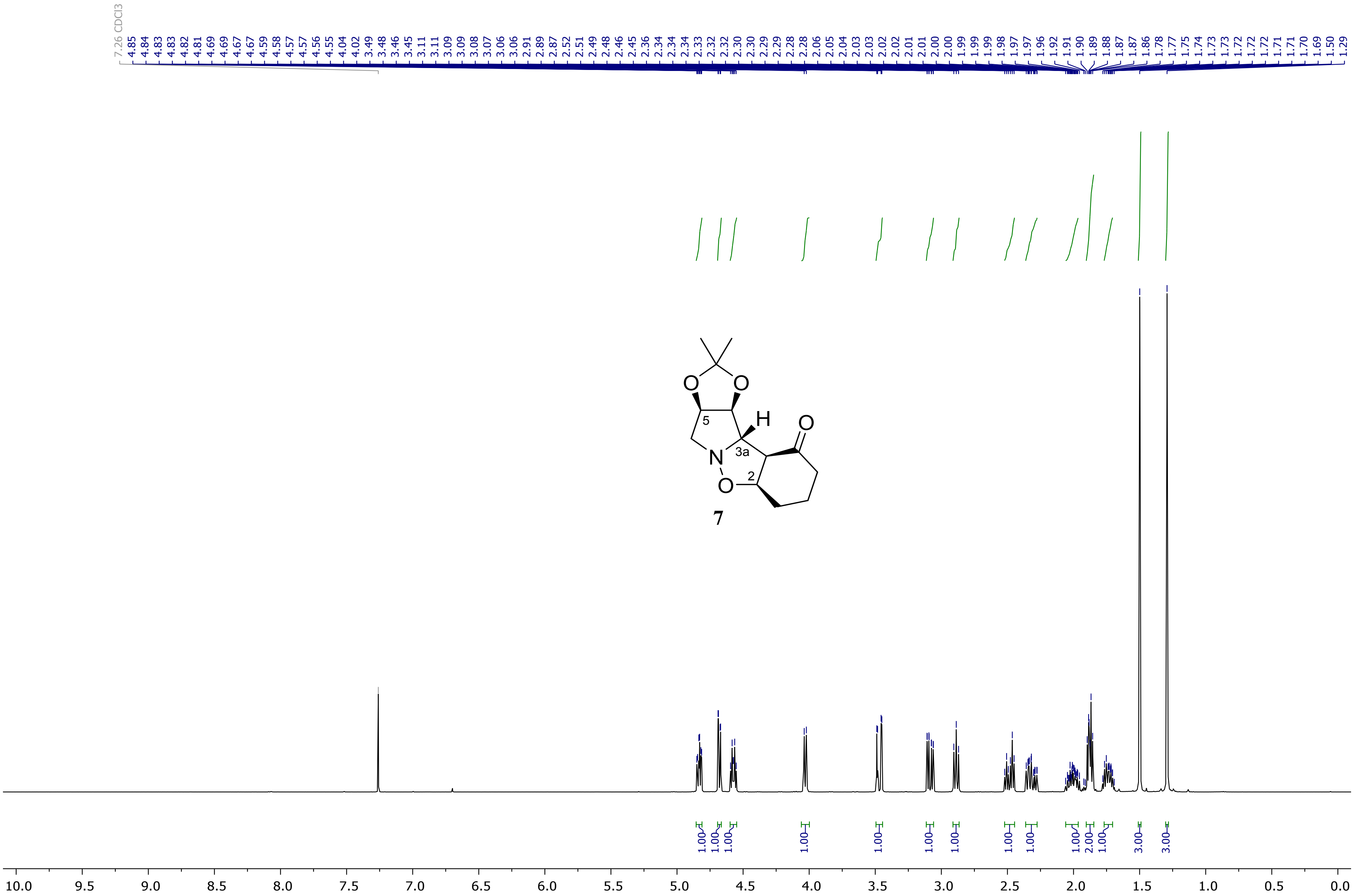

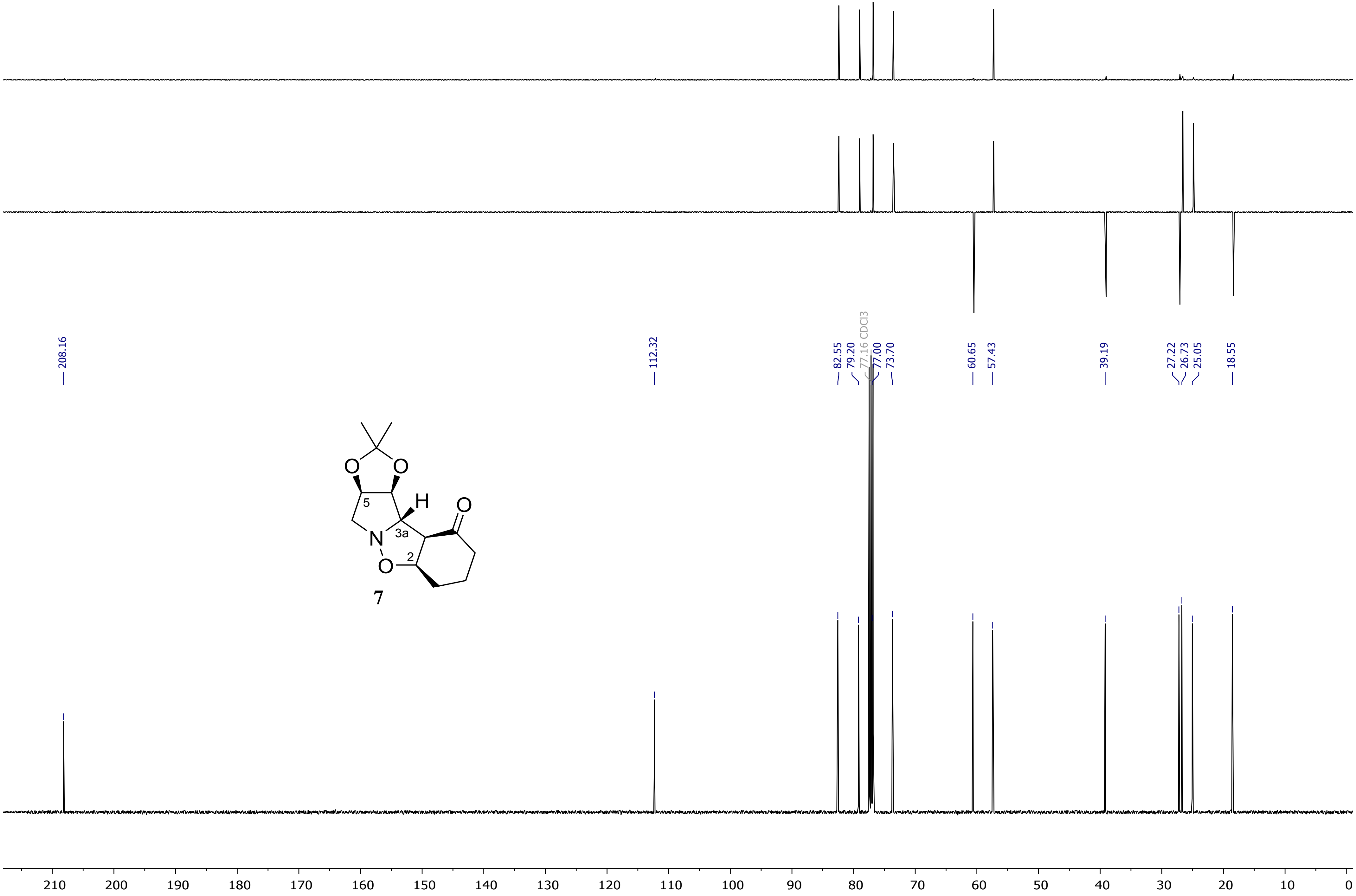
208.16
112.32
82.55
79.20
77.16 CDCl3
77.00
73.70
60.65
57.43
39.19
27.22
26.73
25.05
18.55
O
O
H
O
5
N
3a
2
O
7
210 200 190 180 170 160 150 140 130 120 110 100 90 80 70 60 50 40 30 20 10 0

2D NMR spectra HSQC, HMBC and COSY of **7**

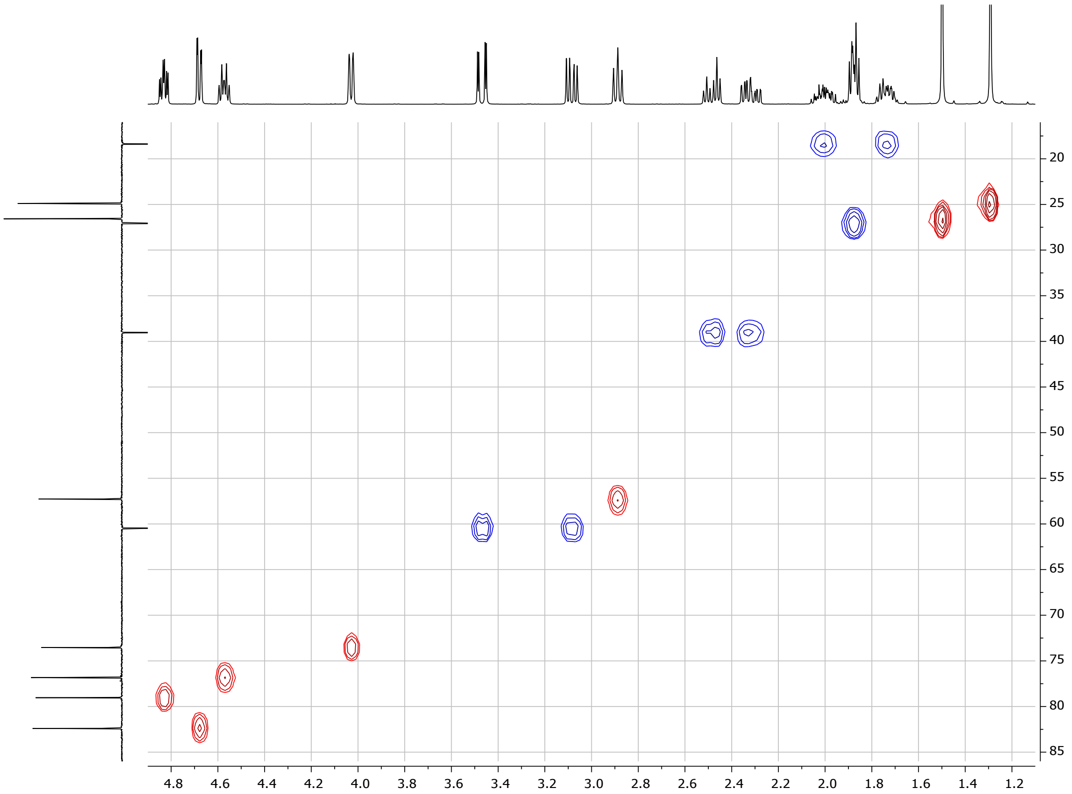

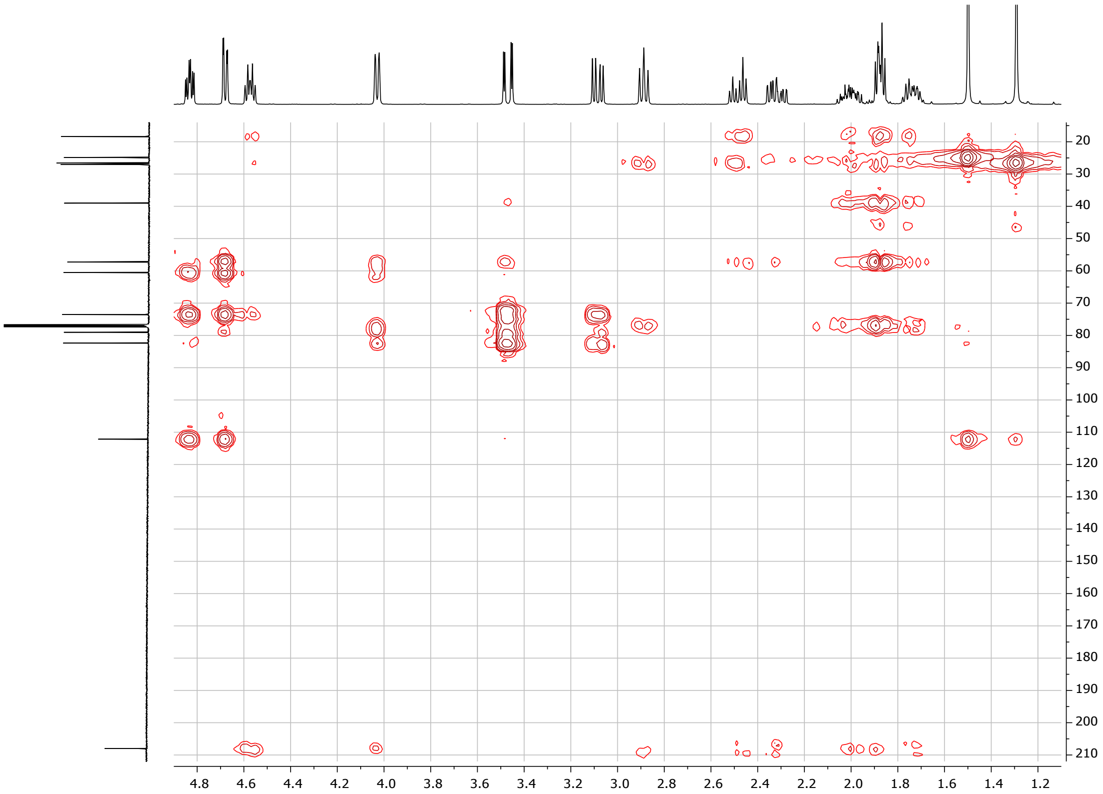

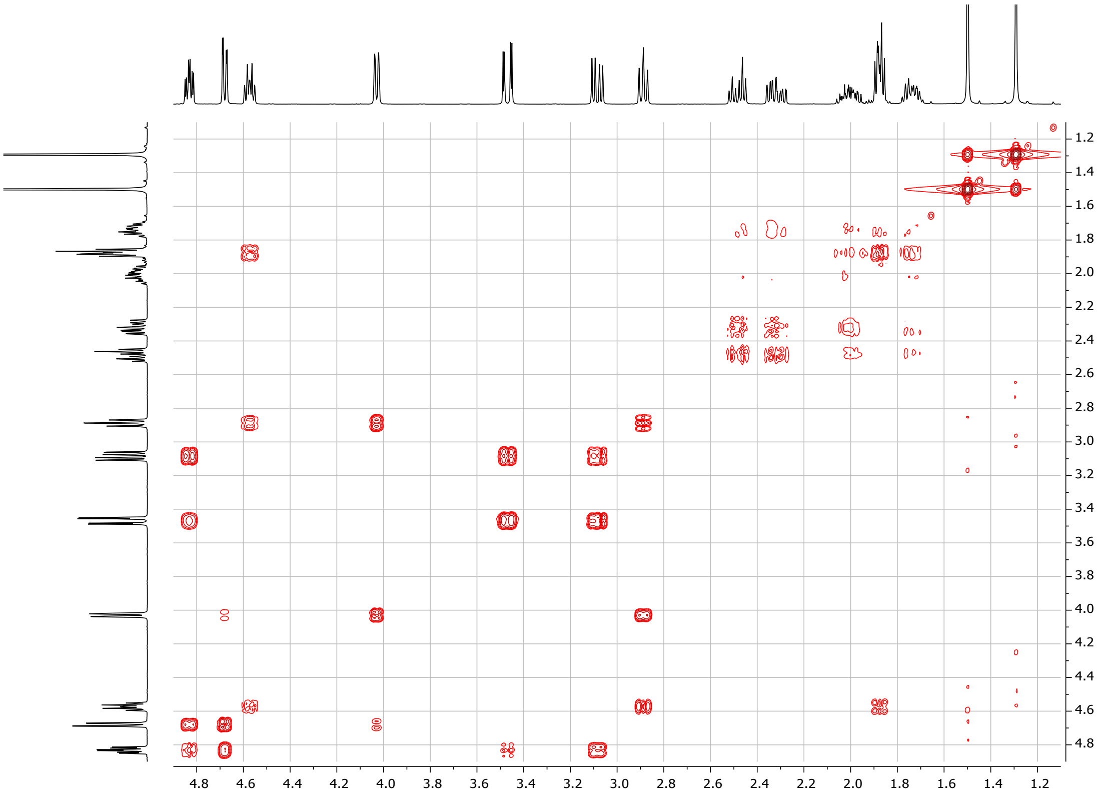

IR spectra of **7**

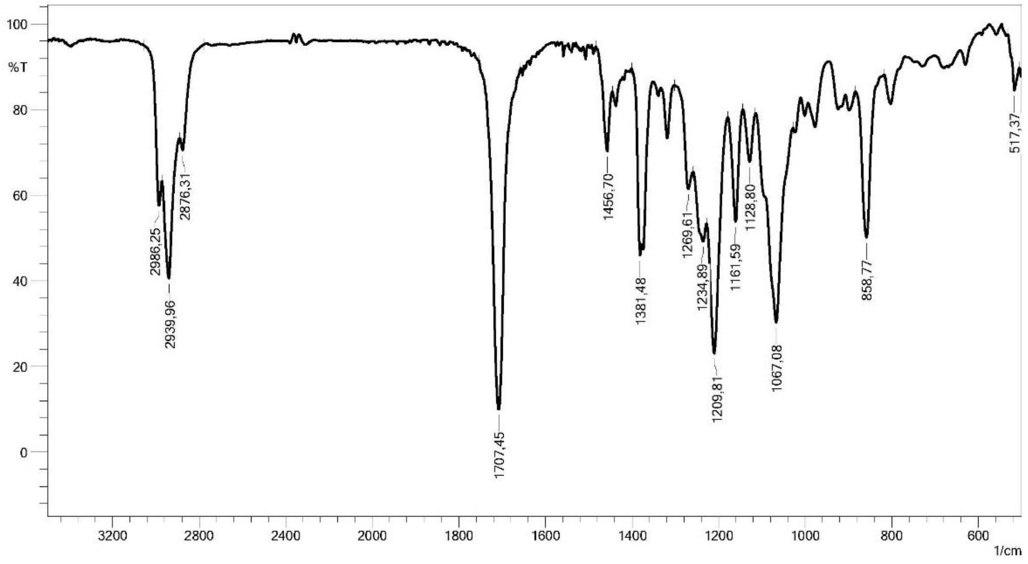

HRMS spectra of **7**

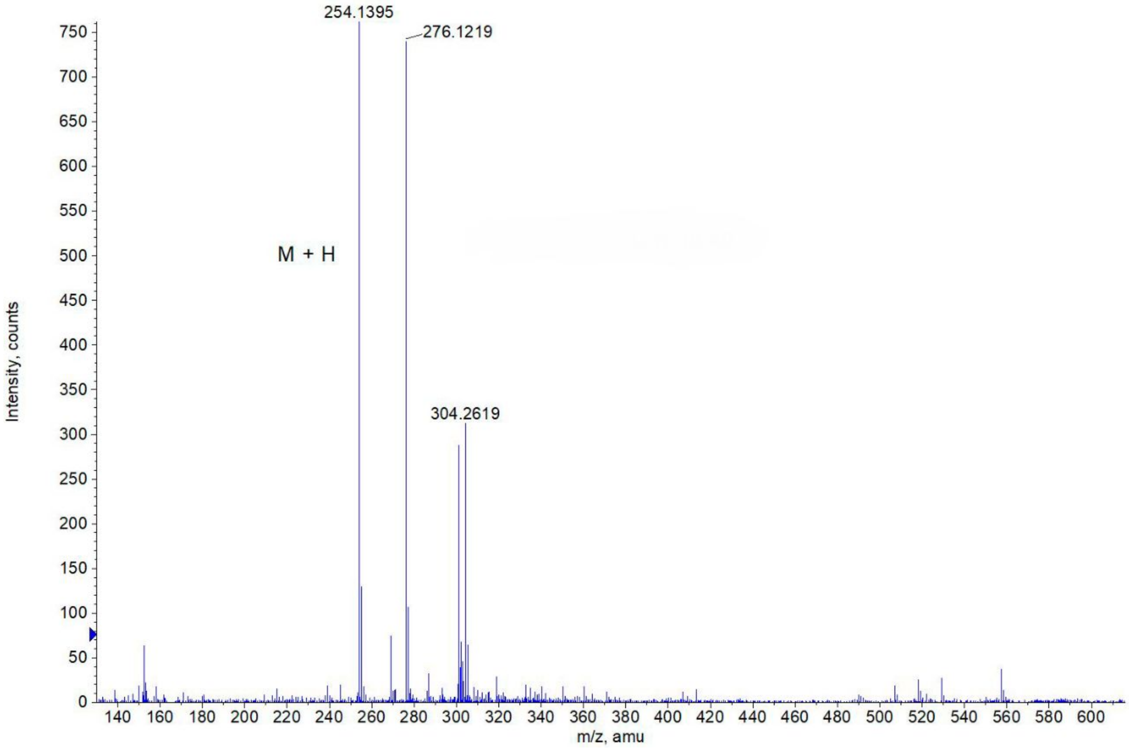

3D representation of the obtained transition states

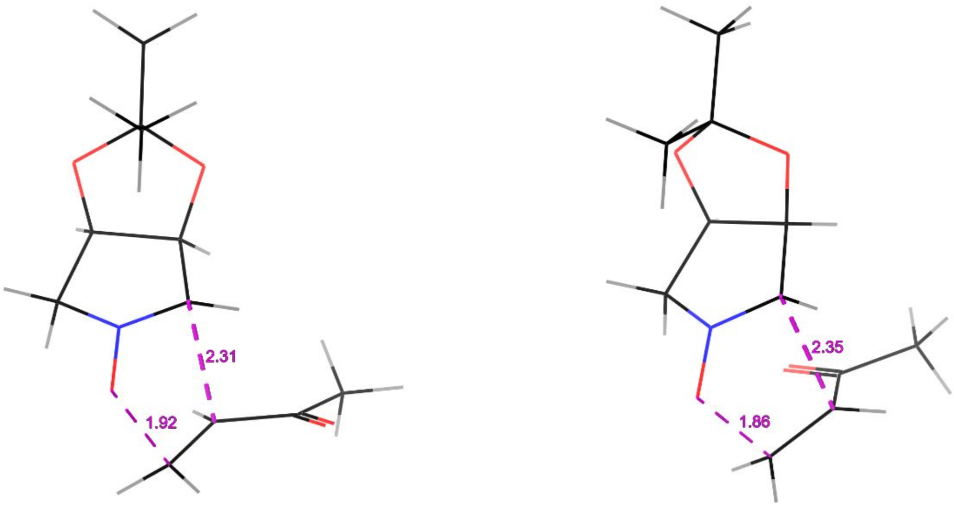

3D representation of the transition states of the reaction between nitrone 1 and MVK (methyl vinyl ketone), *meta-exo* (left hand side), and *meta-endo* (right hand side).

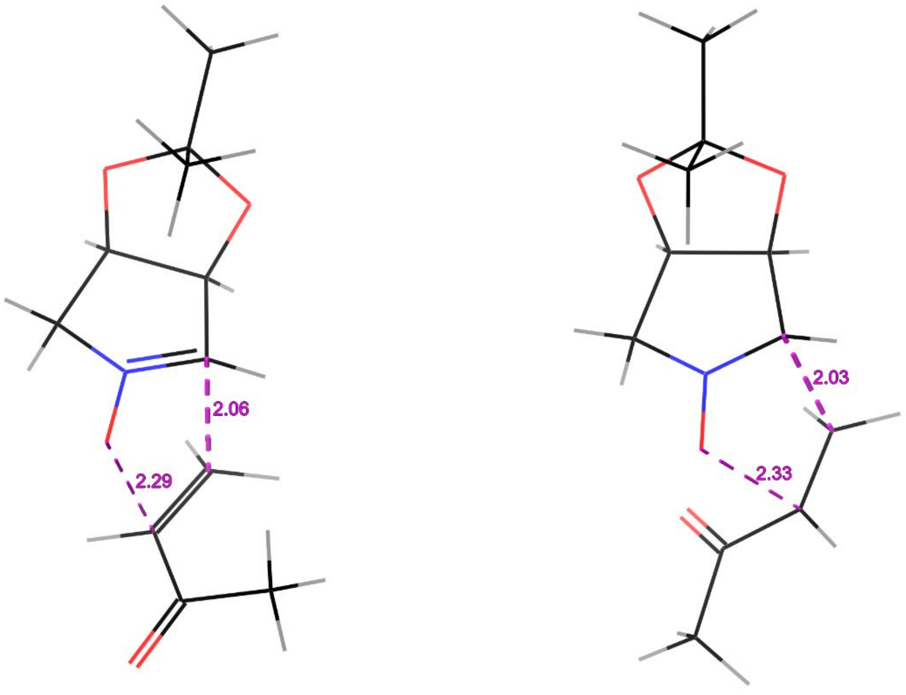

3D representation of the transition states of the reaction between nitrone 1 and MVK (methyl vinyl ketone), *ortho-exo* 2 (left hand side), and *ortho-endo* 3 (right hand side).

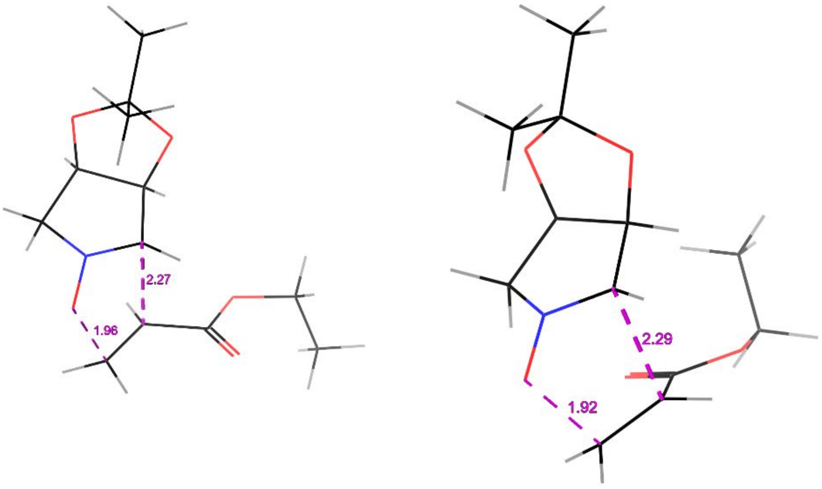

3D representation of the transition states of the reaction between nitrone 1 and EA (ethyl acrylate), *meta-exo* (left hand side), and *meta-endo* (right hand side).

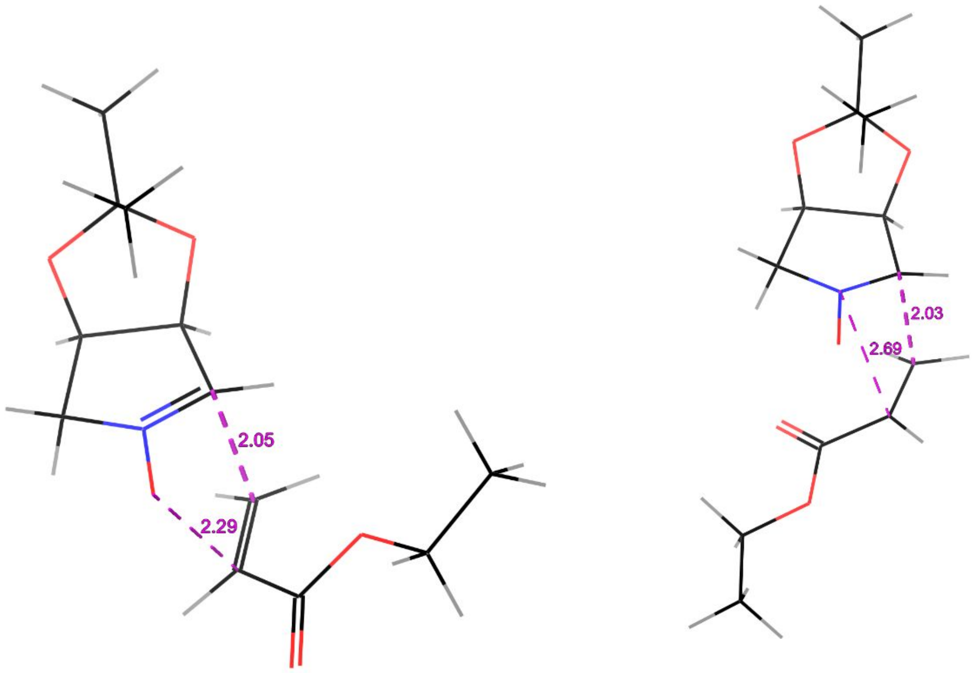

3D representation of the transition states of the reaction between nitrone 1 and EA (ethyl acrylate), *ortho-exo* 4 (left hand side), and *ortho-endo* 5 (right hand side).

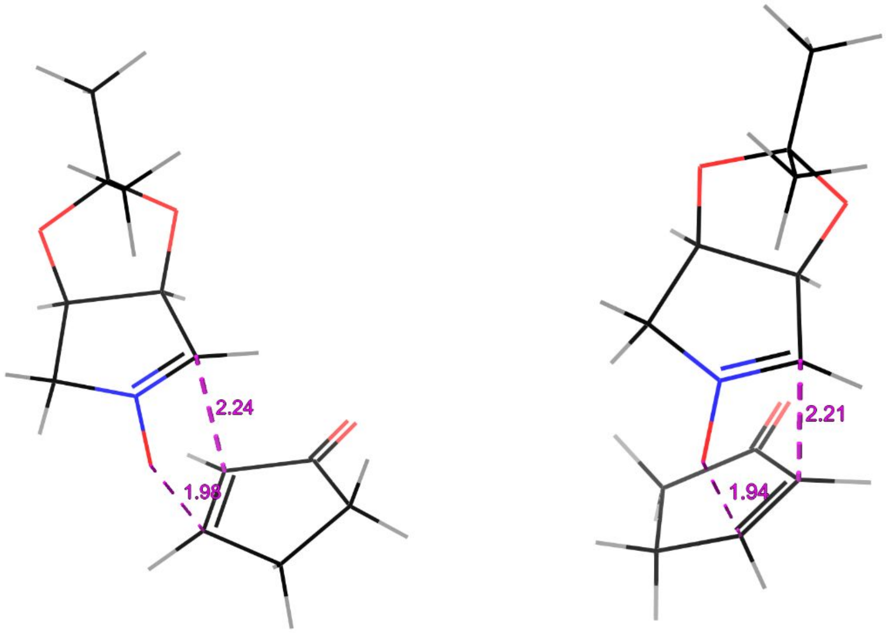

3D representation of the transition states of the reaction between nitrone 1 and CP (2-cyclopenten-1-one), *meta-exo* 6 (left hand side)+, and *meta-endo* (right hand side).

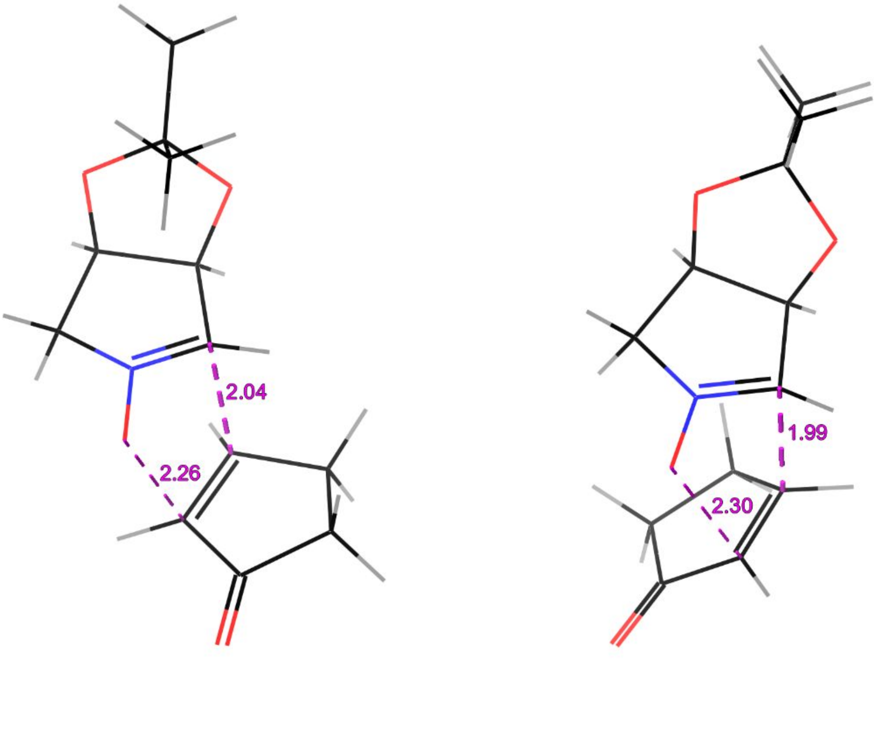

3D representation of the transition states of the reaction between nitrone 1 and CP (2-cyclopenten-1-one), *ortho-exo* (left hand side), and *ortho-endo* (right hand side).

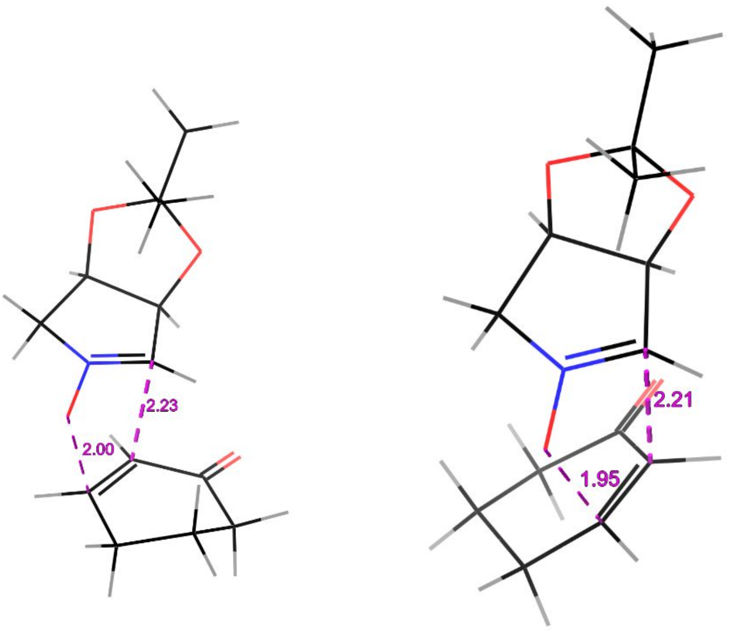

3D representation of the transition states of the reaction between nitrone 1 and CH (2-cyclohexen-1-one), *meta-exo* 7 (left hand side), and *meta-endo* (right hand side).

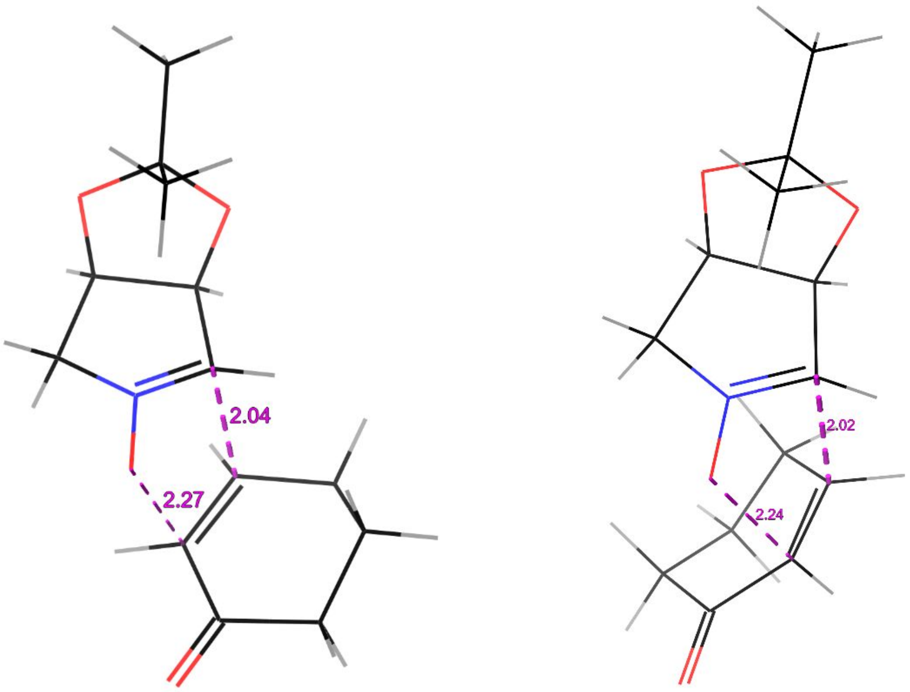

3D representation of the transition states of the reaction between nitrone 1 and CH (2-cyclohexen-1-one), *ortho-exo* (left hand side), and *ortho-endo* (right hand side).

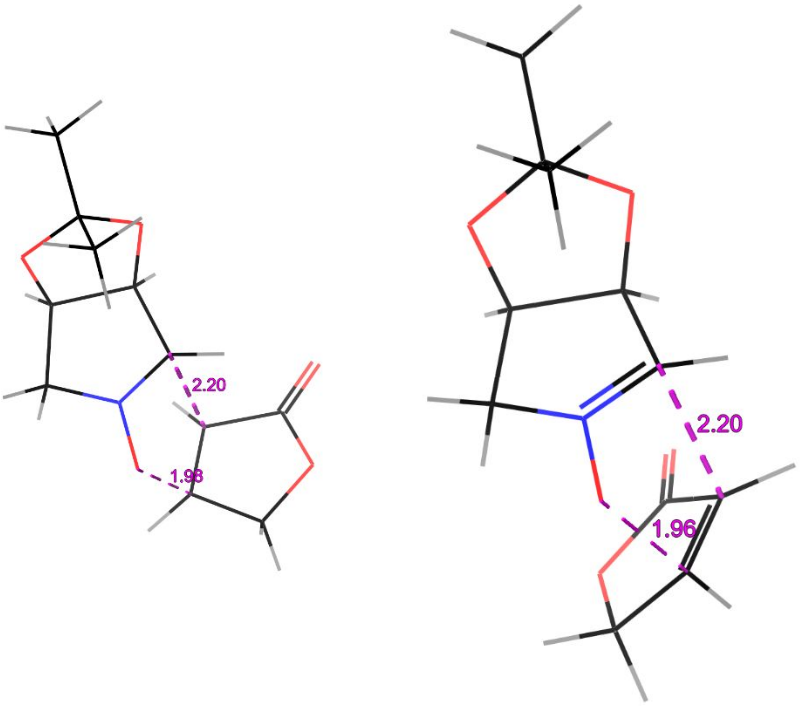

3D representation of the transition states of the reaction between nitrone 1 and FR (2(5*H*)-furanone), *meta-exo* 8 (left hand side), and *meta-endo* 9 (right hand side).

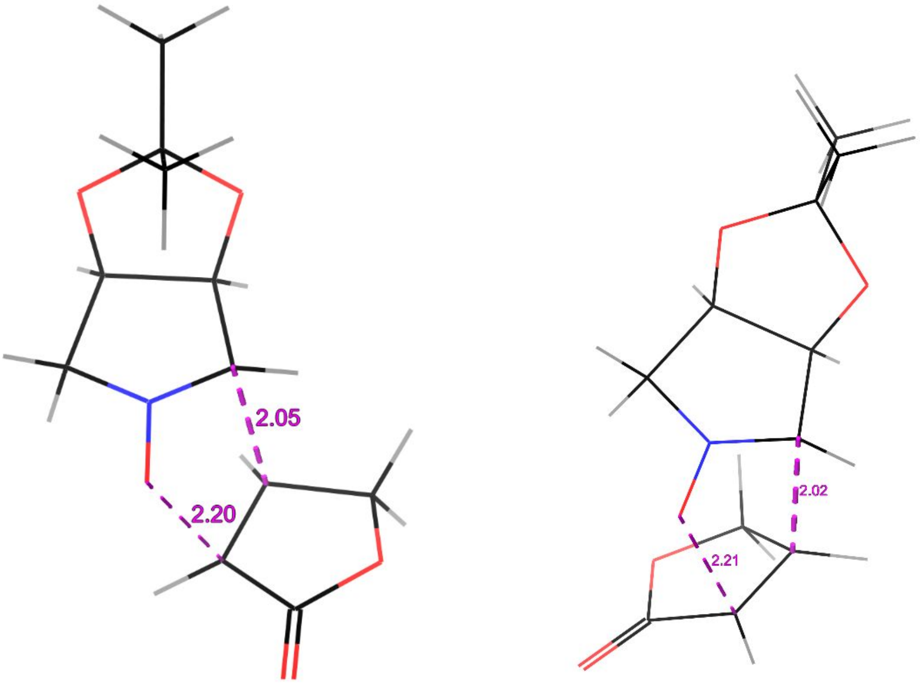

3D representation of the transition states of the reaction between nitrone 1 and FR (2(5*H*)-furanone), *ortho-exo* (left hand side), and *ortho-endo* (right hand side).

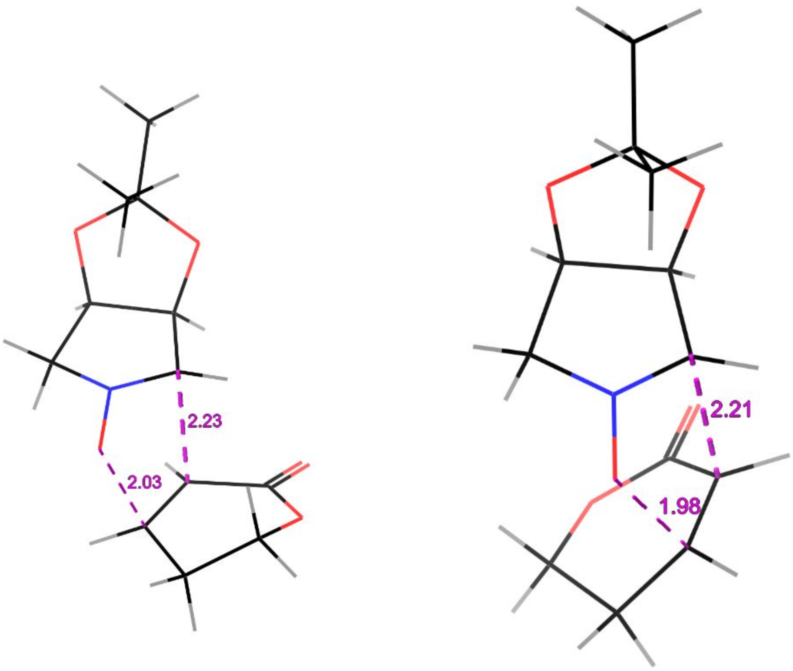

3D representation of the transition states of the reaction between nitrone 1 and PR (5,6-dihydro-2*H*-pyran-2-one), *meta-exo* 10 (left hand side), and *meta-endo* (right hand side).

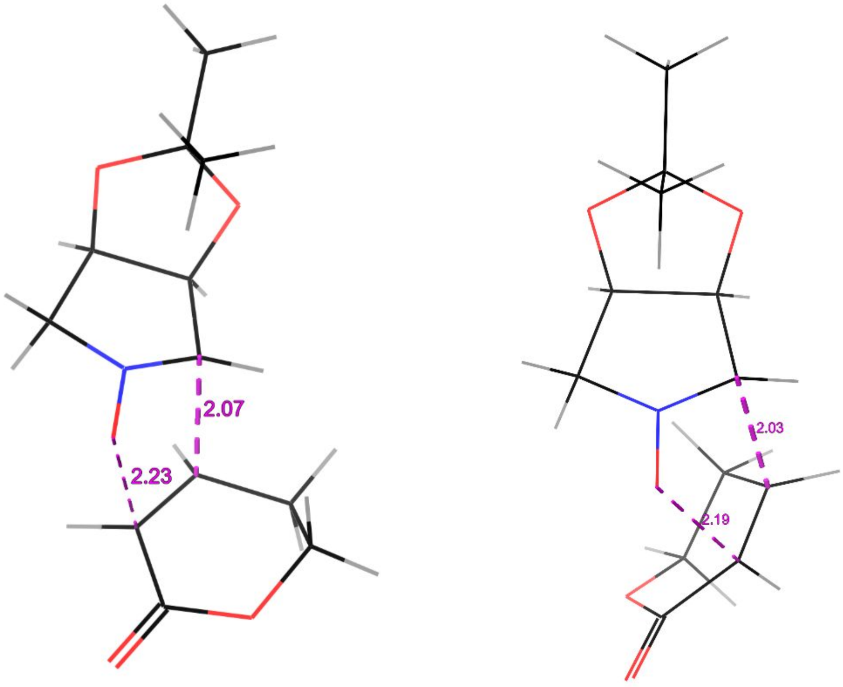

3D representation of the transition states of the reaction between nitrone 1 and PR (5,6-dihydro-2*H*-pyran-2-one), *ortho-exo* (left hand side), and *ortho-endo* (right hand side).

Cartesian coordinates for the optimized geometries

TS_MVK-*meta-exo*

ifreq= -356 cm^{-1}, ΔG= -784,12418 Hartree

C1	0.0207480000	0.3804360000	0.2889220000
C2	-0.6067790000	-0.6195410000	-0.6415820000
O3	-1.3692550000	-1.6187910000	-0.0264110000
C4	-0.5305850000	-2.7233330000	0.2791590000
C5	-1.2965870000	-3.9916420000	-0.0289050000
C6	-0.0461820000	-2.6643090000	1.7197970000
O7	0.5777690000	-2.6215540000	-0.6094850000
C8	0.6105690000	-1.3692500000	-1.2440050000
C9	1.8236990000	-0.5398590000	-0.8503590000
N10	1.3394090000	0.2574600000	0.2793040000
O11	2.0812320000	1.1505600000	0.8097580000
C12	1.4679970000	2.7818530000	-0.0032030000
C13	0.2274620000	2.5078490000	-0.5906820000
C14	-1.0082490000	2.9837510000	0.0463820000
C15	-2.2280930000	3.0846370000	-0.8407730000
O16	-1.0660600000	3.2680590000	1.2334870000
H17	-0.4730490000	0.8201800000	1.1582460000
H18	-1.2382560000	-0.1223690000	-1.3946930000
H19	-2.1834840000	-4.0656140000	0.6166330000
H20	-0.6582480000	-4.8695980000	0.1439140000
H21	-1.6164390000	-3.9784320000	-1.0801560000
H22	-0.9020940000	-2.7037040000	2.4088400000
H23	0.5138210000	-1.7375680000	1.9035780000
H24	0.6171050000	-3.5163730000	1.9269560000
H25	0.5420050000	-1.5040270000	-2.3351660000
H26	2.1389850000	0.1496210000	-1.6531730000
H27	2.6828210000	-1.1516420000	-0.5428160000
H28	1.4665510000	3.3489960000	0.9311630000
H29	2.3541360000	2.8949510000	-0.6322220000
H30	0.1797890000	2.2706510000	-1.6594020000
H31	-2.0494020000	3.8038330000	-1.6569000000
H32	-2.4454470000	2.1131710000	-1.3152850000
H33	-3.0949990000	3.4054670000	-0.2490550000

TS_MVK-*meta-endo*

ifreq= -370 cm^{-1}, ΔG= -784.126704 Hartree

C1	-0.3705180000	0.3249500000	-0.9351080000
C2	0.7485690000	-0.5092350000	-0.3836840000
O3	0.8717290000	-1.7794600000	-0.9716070000
C4	0.2328580000	-2.7450130000	-0.1472620000
C5	1.0803530000	-3.9986520000	-0.1393140000
C6	-1.1918580000	-3.0059180000	-0.6197030000
O7	0.2398950000	-2.1869830000	1.1560350000
C8	0.3239400000	-0.7873650000	1.0871210000
C9	-1.0020570000	-0.0531070000	1.2857930000
N10	-1.3599790000	0.4111790000	-0.0596540000
O11	-2.2998750000	1.2657730000	-0.2247020000
C12	-1.4157200000	2.9016120000	-0.3333770000
C13	-0.1180410000	2.6536300000	-0.8108520000
C14	1.0114490000	2.5995830000	0.1139460000
C15	2.3898610000	2.7662430000	-0.4856180000
O16	0.8772770000	2.4312730000	1.3225800000
H17	-0.5699300000	0.4570430000	-1.9986150000
H18	1.7186390000	0.0028920000	-0.4716320000
H19	1.1422300000	-4.4225170000	-1.1517210000
H20	0.6402620000	-4.7479560000	0.5337350000
H21	2.0915040000	-3.7488960000	0.2101810000
H22	-1.7847380000	-2.0811120000	-0.6198790000
H23	-1.6795340000	-3.7335640000	0.0447380000
H24	-1.1808400000	-3.4099060000	-1.6423270000
H25	1.0705860000	-0.4415890000	1.8157280000
H26	-1.7909970000	-0.7118830000	1.6762250000
H27	-0.8927430000	0.8312190000	1.9293610000
H28	-1.5166930000	3.1608450000	0.7248890000
H29	-2.1501440000	3.3590860000	-1.0000550000
H30	0.1027670000	2.8152880000	-1.8698190000
H31	2.4851500000	2.2489280000	-1.4528430000
H32	2.5700020000	3.8380760000	-0.6754310000
H33	3.1501820000	2.4044800000	0.2195220000

TS_MVK-*ortho-exo* (**2**)

ifreq= -371 cm^{-1}, ΔG= -784.122755 Hartree

C1	0.3426020000	0.0831240000	-0.1083410000
C2	0.3466360000	-1.5720930000	-1.3407110000
C3	0.8030750000	-3.4711420000	0.9766530000
C4	-0.4400100000	-3.5032160000	0.1211550000
O5	-1.2767130000	-4.3798130000	0.2529600000
C6	-0.6431830000	-2.4200130000	-0.8570030000
O7	-1.3907270000	-1.0421050000	0.8161270000
N8	-0.9430340000	-0.0071580000	0.2730730000
C9	-1.8130850000	0.8526680000	-0.5347070000
C10	-0.8767240000	1.9229500000	-1.0796360000
O11	-0.8756100000	3.1058700000	-0.3243040000
C12	0.3312690000	3.2393990000	0.4147480000
C13	0.8453630000	4.6537210000	0.2539140000
C14	0.1063680000	2.8621770000	1.8725690000
O15	1.2545610000	2.3586600000	-0.2069950000
C16	0.5583860000	1.3519240000	-0.8900850000
H17	1.1047290000	-0.2975860000	0.5750600000
H18	0.1610170000	-1.0594880000	-2.2909890000
H19	0.8356350000	-4.3728700000	1.6002370000
H20	1.7217000000	-3.4096610000	0.3729130000
H21	-1.6279980000	-2.4455610000	-1.3324220000
H22	-2.2572520000	0.2275740000	-1.3302710000
H23	-2.6214380000	1.2609300000	0.0875830000
H24	-1.1105670000	2.1679690000	-2.1270160000
H25	0.1259680000	5.3683040000	0.6783250000
H26	1.8082200000	4.7694130000	0.7717530000
H27	0.9819960000	4.8706080000	-0.8145560000
H28	-0.6237300000	3.5467050000	2.3279970000
H29	1.0519430000	2.9296130000	2.4297740000
H30	-0.2793100000	1.8373590000	1.9587280000
H31	1.0871800000	1.1562120000	-1.8360250000
H32	1.3990040000	-1.8081360000	-1.1563470000
H33	0.7633490000	-2.5810630000	1.6251790000

TS_MVK-*ortho-endo* (**3**)

ifreq= -369 cm^{-1}, ΔG= -784.127302 Hartree

C1	0.9369730000	0.0130040000	-0.4489060000
C2	1.7295800000	1.7284760000	0.2914510000
C3	1.0675980000	2.7585630000	-0.3619020000
O4	-0.5466610000	1.4332860000	-1.3887340000
N5	-0.3178060000	0.4543580000	-0.6466860000
C6	-1.2423800000	0.0519620000	0.4285410000
C7	-0.5271350000	-1.1044110000	1.1183130000
O8	-0.9993170000	-2.3750370000	0.7498000000
C9	-0.0893890000	-3.0124260000	-0.1334990000
C10	0.0478670000	-4.4627010000	0.2766940000
C11	-0.5430730000	-2.8551410000	-1.5793340000
O12	1.1569580000	-2.3690270000	0.0837720000
C13	0.9369000000	-1.0786120000	0.5880810000
C14	-0.0681560000	3.4441380000	0.2762690000
C15	-0.6778730000	4.5920590000	-0.4857730000
O16	-0.4943760000	3.1070040000	1.3736620000
H17	1.5968730000	-0.0331700000	-1.3184910000
H18	2.7572240000	1.4841220000	0.0075230000
H19	1.4770320000	1.5842200000	1.3482670000
H20	1.4299220000	3.1420030000	-1.3188190000
H21	-2.2022740000	-0.2519840000	-0.0130030000
H22	-1.3964420000	0.9137620000	1.0910630000
H23	-0.5888270000	-1.0120260000	2.2129130000
H24	0.3769870000	-4.5123860000	1.3237550000
H25	-0.9181160000	-4.9776570000	0.1766720000
H26	0.7882620000	-4.9690940000	-0.3587920000
H27	0.1899030000	-3.3173170000	-2.2564320000
H28	-1.5170860000	-3.3450550000	-1.7224470000
H29	-0.6448940000	-1.7950000000	-1.8487540000
H30	1.6859390000	-0.8872110000	1.3722030000
H31	-1.0710430000	4.2230930000	-1.4461960000
H32	0.0846530000	5.3539560000	-0.7146020000
H33	-1.4897020000	5.0416420000	0.1002510000

TS_EA-*meta-exo*

ifreq= -374 cm^{-1}, ΔG= -898.563692 Hartree

C1	-0.4543150000	-0.1722310000	-0.3772490000
C2	0.5334370000	-0.8515560000	0.5252880000
O3	1.5274270000	-1.6007190000	-0.1161250000
C4	1.0794770000	-2.9380360000	-0.2710840000
C5	2.2441600000	-3.8589700000	0.0218070000
C6	0.4856230000	-3.1618890000	-1.6540810000
O7	0.0785590000	-3.1272600000	0.7236660000
C8	-0.3288540000	-1.9015610000	1.2760530000
C9	-1.7722940000	-1.5482970000	0.9429050000
N10	-1.6605460000	-0.7108160000	-0.2552750000
O11	-2.6831170000	-0.1085710000	-0.7232990000
C12	-2.5192050000	1.6798940000	0.0689250000
C13	-1.2143930000	1.7500060000	0.5542520000
C14	-0.1937450000	2.5355570000	-0.1431000000
O15	-0.2871790000	2.9943670000	-1.2625590000
O16	0.9231000000	2.6430010000	0.6034600000
C17	2.0169250000	3.3717550000	0.0454530000
C18	1.8736250000	4.8582430000	0.2846830000
H19	-0.1937210000	0.3510460000	-1.2996880000
H20	1.0264700000	-0.1142360000	1.1778250000
H21	2.6302030000	-3.6485150000	1.0289850000
H22	1.9203150000	-4.9083120000	-0.0289750000
H23	3.0474490000	-3.6986670000	-0.7114890000
H24	-0.3658630000	-2.4890500000	-1.8231870000
H25	1.2446570000	-2.9729180000	-2.4267120000
H26	0.1322980000	-4.1988520000	-1.7487630000
H27	-0.1525690000	-1.9113490000	2.3631920000
H28	-2.3947170000	-2.4298530000	0.7366190000
H29	-2.2496160000	-0.9475980000	1.7371440000
H30	-3.3485000000	1.5176400000	0.7603330000
H31	-2.7561540000	2.2137500000	-0.8546240000
H32	-0.9996290000	1.5165590000	1.6011730000
H33	2.9095360000	2.9695780000	0.5455150000
H34	2.0869000000	3.1517040000	-1.0305330000
H35	2.7633030000	5.3881990000	-0.0882470000
H36	0.9932490000	5.2523310000	-0.2433810000
H37	1.7689250000	5.0737590000	1.3590080000

TS_EA-*meta-endo*

ifreq= -380 cm^{-1}, ΔG= -898.565999 Hartree

C1	-0.9007330000	0.2072730000	0.9040700000
C2	0.4787580000	0.6224050000	0.4875020000
O3	0.9435610000	1.8134110000	1.0678580000
C4	0.7073230000	2.8963650000	0.1800740000
C5	-0.5969930000	3.6076170000	0.5164750000
C6	1.9045720000	3.8202070000	0.2331200000
O7	0.6414340000	2.3148290000	-1.1132570000
C8	0.3292890000	0.9481130000	-1.0270270000
C9	-1.1137750000	0.5994300000	-1.3856440000
N10	-1.7652970000	0.3810780000	-0.0877680000
O11	-2.9261840000	-0.1490290000	-0.0268820000
C12	-2.5459060000	-1.9993140000	0.2902040000
C13	-1.2669380000	-2.0528480000	0.8562210000
C14	-0.1291650000	-2.4421680000	0.0224280000
O15	-0.1050990000	-2.4485640000	-1.1944780000
O16	0.9383190000	-2.7921710000	0.7667410000
C17	2.0957630000	-3.2727390000	0.0792790000
C18	2.9998690000	-2.1506630000	-0.3806220000
H19	-1.2506540000	0.2153270000	1.9368270000
H20	1.2067740000	-0.1726820000	0.7086420000
H21	-0.7709630000	4.4274250000	-0.1953730000
H22	-0.5493990000	4.0242090000	1.5329270000
H23	-1.4469840000	2.9140050000	0.4636300000
H24	1.7827380000	4.6405350000	-0.4883650000
H25	2.0123870000	4.2455720000	1.2409580000
H26	2.8102530000	3.2509050000	-0.0168870000
H27	1.0336330000	0.3885920000	-1.6602850000
H28	-1.1813800000	-0.3380350000	-1.9575830000
H29	-1.6195600000	1.4085630000	-1.9309820000
H30	-3.4214600000	-2.1471510000	0.9252700000
H31	-2.6576920000	-2.3076310000	-0.7526550000
H32	-1.1417730000	-2.1934540000	1.9320510000
H33	2.6076140000	-3.9193570000	0.8066540000
H34	1.7774180000	-3.8894590000	-0.7747210000
H35	3.3167400000	-1.5215810000	0.4656940000
H36	2.4903240000	-1.5226430000	-1.1253690000
H37	3.9042490000	-2.5668670000	-0.8504110000

TS_EA-*ortho-exo* (**4**)

ifreq= -379 cm^{-1}, ΔG= -898.562990 Hartree

C1	0.3273340000	-0.0620760000	-0.1786690000
C2	0.3239660000	-1.6236060000	-1.5009330000
O3	0.9650080000	-3.2106010000	0.6323010000
C4	-0.2230750000	-3.4961620000	0.0700580000
O5	-0.9008520000	-4.4388130000	0.4132260000
C6	-0.5854530000	-2.5297360000	-0.9735770000
O7	-1.4751160000	-1.1592500000	0.6356930000
N8	-0.9748660000	-0.1205090000	0.1478220000
C9	-1.7787440000	0.8011830000	-0.6604940000
C10	-0.7827560000	1.8658690000	-1.1029970000
O11	-0.7802060000	3.0036480000	-0.2809980000
C12	0.4001560000	3.0629540000	0.5094300000
C13	0.9636480000	4.4664910000	0.4376480000
C14	0.1048970000	2.6238390000	1.9369720000
O15	1.3199080000	2.1825460000	-0.1165210000
C16	0.6227440000	1.2357970000	-0.8810210000
H17	1.0352610000	-0.5376840000	0.5031640000
H18	0.0625830000	-1.1093890000	-2.4322770000
C19	1.3999270000	-4.0715490000	1.6819640000
H20	-1.5802710000	-2.6719160000	-1.3981990000
H21	-2.1994470000	0.2308120000	-1.5080600000
H22	-2.6046580000	1.2068670000	-0.0600960000
H23	-0.9588690000	2.1761340000	-2.1445490000
H24	0.2491140000	5.1834480000	0.8662830000
H25	1.9070860000	4.5270550000	0.9988970000
H26	1.1517760000	4.7287160000	-0.6126630000
H27	-0.6209030000	3.3096370000	2.3972650000
H28	1.0291700000	2.6318200000	2.5327920000
H29	-0.3159760000	1.6094280000	1.9560550000
H30	1.1885440000	1.0696760000	-1.8111400000
H31	1.3911550000	-1.8089250000	-1.3526150000
C32	2.7384940000	-3.5748920000	2.1671860000
H33	0.6452870000	-4.0712760000	2.4855680000
H34	1.4592730000	-5.1047830000	1.3015300000
H35	3.1115320000	-4.2213340000	2.9756880000
H36	3.4797100000	-3.5794160000	1.3534910000
H37	2.6606100000	-2.5484230000	2.5574320000

TS_EA-*ortho-exo* (**5**)

ifreq= -382 cm^{-1}, ΔG= -898.565335 Hartree

C1	0.9817060000	0.7877590000	0.5670990000
C2	2.0277850000	-0.8332080000	-0.0772910000
C3	1.3950070000	-1.9366860000	0.4760660000
O4	-0.4308110000	-0.8151520000	1.3019610000
N5	-0.2300930000	0.2055370000	0.6070440000
C6	-1.0351850000	0.5005330000	-0.5903450000
C7	-0.3936850000	1.7560850000	-1.1664490000
O8	-1.0598310000	2.9455300000	-0.8247290000
C9	-0.3203290000	3.6825510000	0.1374000000
C10	-0.3097650000	5.1393910000	-0.2721680000
C11	-0.8986340000	3.4718410000	1.5307690000
O12	1.0070500000	3.1879390000	0.0527030000
C13	0.9893950000	1.8845090000	-0.4649880000
C14	0.4762780000	-2.7458840000	-0.3321350000
O15	0.0846590000	-2.4645090000	-1.4499910000
O16	0.1180420000	-3.8703700000	0.2962300000
C17	-0.8155810000	-4.7115950000	-0.3761490000
C18	-1.0588480000	-5.9201690000	0.4912600000
H19	1.5232060000	0.8916590000	1.5101930000
H20	1.9134380000	-0.6893070000	-1.1574770000
H21	2.9776770000	-0.4973940000	0.3478600000
H22	1.6699260000	-2.3305470000	1.4557850000
H23	-2.0827640000	0.6581310000	-0.2973840000
H24	-0.9643040000	-0.3627460000	-1.2673910000
H25	-0.3165290000	1.6974570000	-2.2627510000
H26	0.2996250000	5.7277030000	0.4284610000
H27	0.1141910000	5.2278610000	-1.2818980000
H28	-1.3336690000	5.5391340000	-0.2742160000
H29	-0.2957530000	4.0113820000	2.2755010000
H30	-1.9309290000	3.8487840000	1.5688420000
H31	-0.9088660000	2.4057960000	1.7954020000
H32	1.8434480000	1.7810610000	-1.1522780000
H33	-0.4114580000	-4.9872770000	-1.3636380000
H34	-1.7429120000	-4.1446590000	-0.5600080000
H35	-1.4673510000	-5.6259780000	1.4694390000
H36	-0.1242420000	-6.4751590000	0.6615630000
H37	-1.7779190000	-6.5956360000	0.0048750000

TS_CP-*meta-exo* (**6**)

ifreq= -371 cm^{-1}, ΔG= -822.202609 Hartree

C1	-0.1291410000	0.1891810000	0.2232730000
C2	-0.7185460000	-0.9253840000	-0.5892330000
O3	-1.3069350000	-1.9554260000	0.1581940000
C4	-0.3539990000	-2.9817560000	0.3836220000
C5	0.3309290000	-2.8128890000	1.7333520000
C6	-1.0580690000	-4.3155840000	0.2503460000
O7	0.5920700000	-2.8551030000	-0.6715740000
C8	0.5204940000	-1.5825310000	-1.2610360000
C9	1.6980370000	-0.6752060000	-0.9154820000
N10	1.1984040000	0.1626290000	0.1782450000
O11	1.8737970000	1.1687840000	0.5800830000
C12	1.0666400000	2.6541170000	-0.4454210000
C13	0.8200010000	3.6640470000	0.6480540000
C14	-0.6645820000	3.5136100000	0.9874110000
C15	-1.2660200000	2.7325830000	-0.1788220000
O16	-2.4524810000	2.6247800000	-0.4214550000
C17	-0.1470190000	2.1229550000	-0.9086170000
H18	-0.6292340000	0.6115460000	1.0953760000
H19	-1.4705600000	-0.5398440000	-1.2952980000
H20	0.8493040000	-1.8464120000	1.7925690000
H21	1.0708660000	-3.6129930000	1.8796800000
H22	-0.4122150000	-2.8636230000	2.5421900000
H23	-0.3345690000	-5.1370850000	0.3500840000
H24	-1.8249600000	-4.4201230000	1.0311810000
H25	-1.5384220000	-4.3739270000	-0.7361050000
H26	0.4109070000	-1.6991520000	-2.3502390000
H27	2.5891190000	-1.2326080000	-0.5939440000
H28	1.9740090000	-0.0115370000	-1.7538650000
H29	1.9566690000	2.7366190000	-1.0737560000
H30	1.4917180000	3.4968090000	1.5036580000
H31	1.0387220000	4.6747230000	0.2635850000
H32	-0.8251370000	2.9249030000	1.9075050000
H33	-1.2043100000	4.4614060000	1.1265270000
H34	-0.2963290000	1.6750930000	-1.8940120000

TS_CP-*meta-endo*

ifreq= -408 cm^{-1}, ΔG= -822.199086 Hartree

C1	-0.3311130000	-0.0296510000	0.9598740000
C2	0.7665570000	0.8236510000	0.3977760000
O3	0.8305230000	2.1324980000	0.8962480000
C4	0.0759730000	2.9902180000	0.0550120000
C5	-1.3421170000	3.1752980000	0.5791130000
C6	0.8234210000	4.2986940000	-0.0768730000
O7	0.0514800000	2.3508940000	-1.2151990000
C8	0.3747350000	0.9894030000	-1.0968300000
C9	-0.8096170000	0.0527880000	-1.3168320000
N10	-1.2520160000	-0.2965780000	0.0378500000
O11	-2.0803390000	-1.2568740000	0.2323440000
C12	-0.9612000000	-2.7557890000	0.7368380000
C13	-0.7417160000	-3.5269430000	-0.5491000000
C14	0.6775860000	-3.1687440000	-0.9989550000
C15	1.2846640000	-2.3701100000	0.1552440000
O16	2.4413810000	-1.9863250000	0.1978310000
C17	0.2419860000	-2.1580480000	1.1629060000
H18	-0.6146500000	-0.0243160000	2.0132680000
H19	1.7433940000	0.3356510000	0.5487270000
H20	-1.3200110000	3.6286870000	1.5805550000
H21	-1.9083350000	3.8331980000	-0.0962450000
H22	-1.8650080000	2.2114500000	0.6406170000
H23	0.8913880000	4.7979730000	0.8997780000
H24	0.3016760000	4.9621410000	-0.7809350000
H25	1.8366520000	4.0997620000	-0.4518190000
H26	1.2026600000	0.7562280000	-1.7840300000
H27	-1.6298130000	0.5331620000	-1.8696430000
H28	-0.5209970000	-0.8678940000	-1.8440580000
H29	-1.7226060000	-3.1005930000	1.4393140000
H30	-0.8236730000	-4.6050090000	-0.3306540000
H31	-1.5185480000	-3.3060380000	-1.2958100000
H32	0.7269180000	-2.5763550000	-1.9267060000
H33	1.3122830000	-4.0509160000	-1.1789210000
H34	0.5163910000	-1.9792390000	2.2040030000

TS_CP-*ortho-exo*

ifreq= -392 cm^{-1}, ΔG= -822.196786 Hartree

C1	0.3600220000	0.0832670000	-0.0258870000
C2	0.6281290000	1.7923530000	1.0457400000
C3	1.8932530000	2.3689910000	0.4206390000
C4	1.3610200000	3.2510890000	-0.7082870000
C5	-0.0666830000	3.5928930000	-0.2947420000
O6	-0.7274800000	4.5162390000	-0.7237960000
C7	-0.4614900000	2.6060100000	0.7179730000
O8	-1.4867860000	1.1889930000	-0.7117780000
N9	-0.9727290000	0.1745880000	-0.1912740000
C10	-1.7035460000	-0.6351120000	0.7887450000
C11	-0.6987760000	-1.7009920000	1.2043010000
O12	-0.8276000000	-2.8955760000	0.4780460000
C13	0.2719920000	-3.0841740000	-0.4041480000
C14	0.7879740000	-4.4983750000	-0.2462510000
C15	-0.1400610000	-2.7675370000	-1.8350010000
O16	1.2790460000	-2.1930200000	0.0504210000
C17	0.6909020000	-1.1490040000	0.7760710000
H18	0.9936790000	0.3912400000	-0.8619340000
H19	0.6885600000	1.3021130000	2.0244510000
H20	2.4228650000	2.9763350000	1.1737570000
H21	2.6069240000	1.5990290000	0.0857750000
H22	1.9442820000	4.1622550000	-0.9031390000
H23	1.2979800000	2.6981400000	-1.6632410000
H24	-1.4040370000	2.6849890000	1.2602770000
H25	-2.6102280000	-1.0531480000	0.3301720000
H26	-1.9964780000	0.0237640000	1.6257760000
H27	-0.7642260000	-1.9223660000	2.2809420000
H28	1.0641300000	-4.6682200000	0.8035990000
H29	1.6722500000	-4.6534840000	-0.8808520000
H30	0.0111000000	-5.2198950000	-0.5361600000
H31	-0.9333830000	-3.4569190000	-2.1579170000
H32	0.7212440000	-2.8761380000	-2.5098430000
H33	-0.5224820000	-1.7408990000	-1.9140940000
H34	1.3529790000	-0.9124180000	1.6241430000

TS_CP-*ortho-endo*

ifreq= -387 cm^{-1}, ΔG= -822.194561 Hartree

C1	-0.0885870000	-0.0223060000	0.9818100000
C2	0.9177590000	-1.7327510000	1.0670420000
C3	1.7757340000	-1.9326610000	-0.1833710000
C4	1.0723910000	-3.0291780000	-0.9904340000
C5	-0.0169690000	-3.5829220000	-0.0703470000
O6	-0.6580530000	-4.5935480000	-0.2860760000
C7	-0.0733440000	-2.7214450000	1.1101360000
O8	-1.7743230000	-1.3811420000	0.3383100000
N9	-1.0511120000	-0.3912730000	0.1079890000
C10	-0.8051130000	0.0935380000	-1.2565820000
C11	0.1637690000	1.2449840000	-1.0587840000
O12	-0.5257700000	2.4677480000	-1.0253270000
C13	0.0271010000	3.2624810000	0.0062560000
C14	-1.0570940000	4.1572180000	0.5578580000
C15	1.2450090000	4.0258690000	-0.4966430000
O16	0.4132040000	2.3348440000	1.0101790000
C17	0.7108020000	1.1049120000	0.3952600000
H18	-0.3662760000	0.0020890000	2.0388110000
H19	1.4194860000	-1.4044730000	1.9841480000
H20	1.9276800000	-1.0051220000	-0.7585800000
H21	2.7837700000	-2.2551020000	0.1238960000
H22	0.6110100000	-2.6795960000	-1.9279230000
H23	1.7442020000	-3.8535600000	-1.2753480000
H24	-0.6854400000	-2.9765910000	1.9751550000
H25	-0.3972080000	-0.7273370000	-1.8647900000
H26	-1.7550160000	0.4239900000	-1.7001800000
H27	0.9636980000	1.2484290000	-1.8195560000
H28	-1.8972220000	3.5350070000	0.8953450000
H29	-0.6729550000	4.7400700000	1.4066680000
H30	-1.4085660000	4.8502550000	-0.2193660000
H31	1.6942600000	4.6056800000	0.3221870000
H32	0.9564150000	4.7123710000	-1.3054290000
H33	2.0068220000	3.3327490000	-0.8845320000
H34	1.7955310000	0.9038520000	0.4329100000

TS_CH-*meta-exo* (**7**)

ifreq= -376 cm^{-1}, ΔG= -861.457109 Hartree

C1	-0.0311730000	0.0425130000	0.2334340000
C2	-0.7093890000	-1.0707590000	-0.5052710000
O3	-1.2748470000	-2.0680250000	0.3013500000
C4	-0.3320350000	-3.1093800000	0.4944620000
C5	0.4303910000	-2.9296140000	1.8002390000
C6	-1.0685830000	-4.4298840000	0.4277090000
O7	0.5605990000	-3.0258040000	-0.6105350000
C8	0.4605200000	-1.7774710000	-1.2460470000
C9	1.6768140000	-0.8830000000	-1.0278230000
N10	1.2889780000	-0.0108310000	0.0849700000
O11	2.0049510000	1.0029090000	0.3865720000
C12	1.1163410000	2.4725170000	-0.6335350000
C13	1.1641300000	3.5394410000	0.4185990000
C14	0.0068220000	3.4100260000	1.3995020000
C15	-1.3332310000	3.3591990000	0.6732310000
C16	-1.3914870000	2.3510510000	-0.4593170000
O17	-2.4604030000	1.9533150000	-0.8991160000
C18	-0.1101130000	1.8974650000	-1.0066200000
H19	-0.4566900000	0.5070550000	1.1232080000
H20	-1.4979630000	-0.6653290000	-1.1594670000
H21	-0.2665280000	-2.9502950000	2.6505250000
H22	1.1629830000	-3.7407870000	1.9212300000
H23	0.9693650000	-1.9725410000	1.8088290000
H24	-1.7926120000	-4.5032390000	1.2518260000
H25	-1.6043230000	-4.4974610000	-0.5292420000
H26	-0.3568190000	-5.2641450000	0.5039470000
H27	0.2632450000	-1.9336650000	-2.3177580000
H28	2.5844910000	-1.4480720000	-0.7740760000
H29	1.8859440000	-0.2424890000	-1.9026480000
H30	1.9245710000	2.4826970000	-1.3709180000
H31	2.1322370000	3.4942190000	0.9413960000
H32	1.1293000000	4.5253490000	-0.0835370000
H33	0.0155980000	4.2431590000	2.1190830000
H34	0.1529970000	2.4918540000	1.9927130000
H35	-2.1766370000	3.1560220000	1.3510370000
H36	-1.5489760000	4.3392470000	0.2070270000
H37	-0.1916130000	1.3739520000	-1.9649280000

TS_CH-*meta-endo*

ifreq= -404 cm^{-1}, ΔG= -861.451301 Hartree

C1	-0.2938270000	0.2182210000	0.9508340000
C2	0.7931750000	1.0754970000	0.3722200000
O3	0.8350500000	2.3941530000	0.8451340000
C4	0.0319250000	3.2142560000	0.0121930000
C5	0.7527600000	4.5303110000	-0.1889110000
C6	-1.3634310000	3.3914460000	0.5938670000
O7	-0.0474040000	2.5361300000	-1.2363830000
C8	0.3911460000	1.2064490000	-1.1196800000
C9	-0.7190890000	0.1856670000	-1.3264280000
N10	-1.2009900000	-0.0923400000	0.0281400000
O11	-2.0362140000	-1.0403680000	0.2588690000
C12	-0.9648950000	-2.4800140000	1.0263570000
C13	-1.1741240000	-3.5694580000	0.0149350000
C14	-0.2750050000	-3.4318240000	-1.2036980000
C15	1.1780440000	-3.1864120000	-0.8113180000
C16	1.4062560000	-2.1573880000	0.2843710000
O17	2.5074200000	-1.6352000000	0.4143200000
C18	0.3043930000	-1.8896150000	1.2132250000
H19	-0.5936500000	0.2675150000	1.9985600000
H20	1.7758570000	0.6032910000	0.5321710000
H21	1.7490120000	4.3370710000	-0.6102550000
H22	0.1863410000	5.1699140000	-0.8804400000
H23	0.8632040000	5.0544180000	0.7711170000
H24	-1.8645460000	2.4196420000	0.7004120000
H25	-1.9702050000	4.0214880000	-0.0724370000
H26	-1.3045330000	3.8721120000	1.5810420000
H27	1.2316160000	1.0347310000	-1.8104070000
H28	-0.3311840000	-0.7472250000	-1.7655780000
H29	-1.5395790000	0.5600660000	-1.9541700000
H30	-1.6148570000	-2.5397690000	1.9046160000
H31	-0.9608710000	-4.5285560000	0.5258030000
H32	-2.2339670000	-3.6108770000	-0.2791640000
H33	-0.6466090000	-2.6021700000	-1.8256100000
H34	-0.3471780000	-4.3314880000	-1.8343360000
H35	1.6137840000	-4.1187380000	-0.4031130000
H36	1.8094530000	-2.9162550000	-1.6715820000
H37	0.6302030000	-1.5865890000	2.2115200000

TS_CH-*ortho-exo*

ifreq= -396 cm^{-1}, ΔG= -861.449382 Hartree

C1	0.3708140000	0.1300620000	-0.0009800000
C2	0.5276820000	-1.5827980000	-1.0911990000
C3	1.9165610000	-2.0531060000	-0.7049230000
C4	1.8989890000	-2.7635990000	0.6431920000
C5	0.9094500000	-3.9204160000	0.6415900000
C6	-0.4703020000	-3.5561910000	0.1272670000
O7	-1.4304220000	-4.2758860000	0.3453130000
C8	-0.5756830000	-2.3616300000	-0.7169750000
O9	-1.3629450000	-0.9911040000	0.9165180000
N10	-0.9271790000	0.0331010000	0.3462160000
C11	-1.7902530000	0.8422900000	-0.5194510000
C12	-0.8553520000	1.8938100000	-1.1037530000
O13	-0.8932990000	3.1248610000	-0.4305840000
C14	0.2774890000	3.3114270000	0.3534940000
C15	0.7777520000	4.7248520000	0.1480560000
C16	-0.0032330000	2.9995410000	1.8175420000
O17	1.2381120000	2.4177260000	-0.1879420000
C18	0.5836520000	1.3624920000	-0.8395290000
H19	1.1133350000	-0.1631180000	0.7439160000
H20	0.4519110000	-1.0722930000	-2.0602200000
H21	2.2878530000	-2.7425570000	-1.4853540000
H22	2.6239660000	-1.2068090000	-0.6938690000
H23	1.6127140000	-2.0493290000	1.4348540000
H24	2.9077720000	-3.1204610000	0.9026170000
H25	0.7913030000	-4.3763340000	1.6363190000
H26	1.2690430000	-4.7280340000	-0.0238140000
H27	-1.5384750000	-2.2535180000	-1.2230620000
H28	-2.2175650000	0.1754800000	-1.2897420000
H29	-2.6130140000	1.2748010000	0.0669030000
H30	-1.0632720000	2.0667370000	-2.1706820000
H31	0.0333390000	5.4467440000	0.5126630000
H32	1.7181250000	4.8777710000	0.6966560000
H33	0.9528060000	4.8957640000	-0.9231490000
H34	-0.7620050000	3.6925310000	2.2088260000
H35	0.9170590000	3.1095440000	2.4093610000
H36	-0.3761190000	1.9736100000	1.9385220000
H37	1.1465210000	1.1324920000	-1.7583440000

TS_CH-*ortho*-*endo*

ifreq= -399 cm^{-1}, ΔG= -861.445219 Hartree

C1	-0.1774640000	0.2623720000	0.9291240000
C2	0.6193930000	-1.5679490000	1.2127720000
C3	1.8341880000	-1.7262530000	0.3054390000
C4	1.9129920000	-3.1186380000	-0.3142270000
C5	0.6005040000	-3.4854560000	-0.9978910000
C6	-0.5671200000	-3.4259470000	-0.0282430000
O7	-1.4866910000	-4.2247290000	-0.0897530000
C8	-0.4898850000	-2.4058190000	1.0223420000
O9	-1.7925140000	-0.9573850000	-0.0789890000
N10	-0.9530900000	-0.0289060000	-0.1388900000
C11	-0.3698340000	0.4112800000	-1.4100890000
C12	0.6833000000	1.4409790000	-1.0036690000
O13	0.2855590000	2.7725310000	-1.1898740000
C14	-0.0825950000	3.3592390000	0.0506180000
C15	0.4643230000	4.7687590000	0.0965810000
C16	-1.5925030000	3.3117470000	0.2437970000
O17	0.5842850000	2.5901260000	1.0394490000
C18	0.8400710000	1.3037560000	0.5415230000
H19	-0.6738920000	0.3187380000	1.9013770000
H20	0.8450070000	-1.3096180000	2.2537410000
H21	1.8180670000	-0.9908560000	-0.5168600000
H22	2.7514110000	-1.5074440000	0.8738280000
H23	2.1256080000	-3.8586450000	0.4765900000
H24	2.7514050000	-3.1714340000	-1.0271670000
H25	0.3921570000	-2.7913860000	-1.8321520000
H26	0.6187960000	-4.4969810000	-1.4287480000
H27	-1.2559040000	-2.4890330000	1.7964140000
H28	-1.1560190000	0.8484830000	-2.0426320000
H29	0.0514110000	-0.4605340000	-1.9354680000
H30	1.6273080000	1.2822350000	-1.5475730000
H31	1.5529020000	4.7388930000	-0.0492560000
H32	0.0094430000	5.3782430000	-0.6970290000
H33	0.2422610000	5.2294720000	1.0695030000
H34	-1.9661570000	2.2797800000	0.1996480000
H35	-2.0901510000	3.8965430000	-0.5431110000
H36	-1.8587060000	3.7350620000	1.2230200000
H37	1.8546560000	1.0157160000	0.8572250000

TS_FR-*meta*-*exo* (**8**)

ifreq= -388 cm^{-1}, ΔG= -858.134476 Hartree

C1	-0.1144520000	0.2087790000	0.1950470000
C2	-0.7108570000	-0.9167620000	-0.6019960000
O3	-1.3068680000	-1.9296740000	0.1591260000
C4	-0.3617420000	-2.9589380000	0.4038090000
C5	0.3065030000	-2.7814070000	1.7599200000
C6	-1.0690850000	-4.2903890000	0.2729870000
O7	0.5997270000	-2.8476680000	-0.6395400000
C8	0.5245920000	-1.5930370000	-1.2626150000
C9	1.7038810000	-0.6803870000	-0.9433980000
N10	1.2153560000	0.1641410000	0.1502380000
O11	1.8988010000	1.1641170000	0.5482950000
C12	1.0650990000	2.6542010000	-0.4624380000
C13	0.7284610000	3.6098920000	0.6436990000
O14	-0.6759120000	3.4983180000	0.8334170000
C15	-1.2314650000	2.7307220000	-0.1426180000
O16	-2.4226800000	2.6003270000	-0.2493390000
C17	-0.1405330000	2.1132400000	-0.9132990000
H18	-0.6045000000	0.6325950000	1.0745910000
H19	-1.4592930000	-0.5381260000	-1.3154490000
H20	0.8229940000	-1.8138470000	1.8176510000
H21	1.0451740000	-3.5796790000	1.9217040000
H22	-0.4465990000	-2.8259370000	2.5597670000
H23	-0.3506950000	-5.1133770000	0.3949860000
H24	-1.8498370000	-4.3826970000	1.0413330000
H25	-1.5321140000	-4.3591260000	-0.7211160000
H26	0.4105840000	-1.7367720000	-2.3480330000
H27	2.5991440000	-1.2336870000	-0.6275170000
H28	1.9681340000	-0.0249610000	-1.7919770000
H29	1.9610850000	2.7906660000	-1.0688720000
H30	1.2436200000	3.3625180000	1.5852350000
H31	0.9749810000	4.6507630000	0.3719440000
H32	-0.3301060000	1.7047700000	-1.9066410000

TS_FR-*meta-endo* (**9**)

ifreq= -412 cm^{-1}, ΔG= -858.133436 Hartree

C1	-0.4138280000	0.0104750000	-0.9489150000
C2	0.7274560000	-0.8593520000	-0.5055840000
O3	0.6919170000	-2.1744240000	-0.9889900000
C4	0.0301610000	-3.0036710000	-0.0458550000
C5	0.7635970000	-4.3267160000	0.0157590000
C6	-1.4435560000	-3.1667640000	-0.3939110000
O7	0.1682700000	-2.3433180000	1.2054450000
C8	0.5137380000	-0.9934970000	1.0275560000
C9	-0.6087950000	-0.0194770000	1.3721360000
N10	-1.2206340000	0.2939530000	0.0714600000
O11	-2.0794100000	1.2358210000	-0.0489980000
C12	-1.0111930000	2.7337800000	-0.7110630000
C13	-0.6087210000	3.4519830000	0.5466140000
O14	0.7103970000	3.0023780000	0.8325420000
C15	1.2177670000	2.3178630000	-0.2337300000
O16	2.3626490000	1.9372900000	-0.2455260000
C17	0.1433410000	2.1215450000	-1.2167450000
H18	-0.8208280000	-0.0111020000	-1.9614550000
H19	1.6924230000	-0.4028280000	-0.7760580000
H20	0.3138260000	-4.9730280000	0.7826750000
H21	0.7089580000	-4.8382390000	-0.9558810000
H22	1.8174580000	-4.1453510000	0.2684260000
H23	-1.5495890000	-3.6392240000	-1.3811990000
H24	-1.9505000000	-2.1922580000	-0.4125890000
H25	-1.9380780000	-3.7991640000	0.3576170000
H26	1.4234020000	-0.7738230000	1.6067680000
H27	-0.2203200000	0.9081170000	1.8181990000
H28	-1.3595060000	-0.4593330000	2.0433390000
H29	-1.8298220000	3.1153700000	-1.3224610000
H30	-0.5878810000	4.5447800000	0.3863940000
H31	-1.2601860000	3.2436390000	1.4077280000
H32	0.3860730000	1.9651270000	-2.2673190000

TS_FR-*ortho-exo*

ifreq= -396 cm^{-1}, ΔG= -858.127630 Hartree

C1	0.3441810000	0.0952620000	-0.0197090000
C2	0.5746240000	1.8032900000	1.0937170000
C3	1.8190920000	2.4203230000	0.4914400000
O4	1.3407840000	3.2891280000	-0.5193070000
C5	0.0058390000	3.5281550000	-0.3506590000
O6	-0.5734630000	4.3605420000	-0.9934600000
C7	-0.4867040000	2.6102300000	0.6880440000
O8	-1.5066060000	1.2075300000	-0.6637170000
N9	-0.9889840000	0.1798520000	-0.1683980000
C10	-1.7038350000	-0.6349230000	0.8190060000
C11	-0.6889050000	-1.6979170000	1.2185120000
O12	-0.8250140000	-2.8954800000	0.5007280000
C13	0.2520810000	-3.0757290000	-0.4108110000
C14	0.7775830000	-4.4880650000	-0.2692070000
C15	-0.1952160000	-2.7565470000	-1.8299640000
O16	1.2661240000	-2.1801470000	0.0207020000
C17	0.6926470000	-1.1426630000	0.7657870000
H18	0.9634720000	0.4380510000	-0.8539570000
H19	0.6235870000	1.3438420000	2.0859280000
H20	2.3682770000	3.0072860000	1.2492580000
H21	2.5286340000	1.7072160000	0.0422360000
H22	-1.4446000000	2.7685420000	1.1788390000
H23	-2.6154960000	-1.0552860000	0.3727280000
H24	-1.9879470000	0.0195180000	1.6627250000
H25	-0.7358380000	-1.9159750000	2.2967550000
H26	1.0795260000	-4.6600290000	0.7732350000
H27	1.6464430000	-4.6390290000	-0.9256040000
H28	-0.0036820000	-5.2113590000	-0.5426150000
H29	-0.9928220000	-3.4485120000	-2.1364620000
H30	0.6509940000	-2.8612830000	-2.5242360000
H31	-0.5810500000	-1.7304520000	-1.8992100000
H32	1.3721320000	-0.9111190000	1.6015500000

TS_FR-*ortho-endo*

ifreq= -397 cm^{-1}, ΔG= -858.128086 Hartree

C1	-0.0720300000	-0.0246670000	1.0494700000
C2	0.9093150000	-1.7858760000	1.1520750000
C3	1.7605950000	-1.9712390000	-0.0898210000
O4	1.0247410000	-2.8374660000	-0.9333820000
C5	0.0042660000	-3.4312460000	-0.2306790000
O6	-0.6202430000	-4.3476240000	-0.6926790000
C7	-0.0972580000	-2.7499530000	1.0643390000
O8	-1.7530130000	-1.4083510000	0.4639000000
N9	-1.0447530000	-0.4124370000	0.1986140000
C10	-0.7954710000	0.0143700000	-1.1872860000
C11	0.1576770000	1.1841520000	-1.0265080000
O12	-0.5505530000	2.3963210000	-0.9978590000
C13	0.0233470000	3.2237090000	-0.0055480000
C14	-1.0551140000	4.1144710000	0.5629610000
C15	1.2120470000	3.9933840000	-0.5650240000
O16	0.4602410000	2.3267200000	1.0084850000
C17	0.7304280000	1.0789050000	0.4211310000
H18	-0.3233390000	0.0119850000	2.1126080000
H19	1.4038450000	-1.5404020000	2.0963480000
H20	1.9907580000	-1.0495060000	-0.6475800000
H21	2.7246070000	-2.4443530000	0.1685490000
H22	-0.7092430000	-3.1498640000	1.8697170000
H23	-0.3657350000	-0.8277850000	-1.7507930000
H24	-1.7451540000	0.3114610000	-1.6536140000
H25	0.9440140000	1.1877250000	-1.8013590000
H26	-1.8759080000	3.4880620000	0.9381160000
H27	-0.6520800000	4.7176320000	1.3885270000
H28	-1.4401130000	4.7884250000	-0.2151450000
H29	1.6810700000	4.5951080000	0.2264280000
H30	0.8856460000	4.6600550000	-1.3761820000
H31	1.9696420000	3.3029720000	-0.9659090000
H32	1.8139250000	0.8675680000	0.4454610000

TS_PR-*meta-exo* (**10**)

ifreq= -378 cm^{-1}, ΔG= -897.385826 Hartree

C1	-0.0624680000	0.0348900000	0.2512890000
C2	-0.7184640000	-1.0900170000	-0.4917420000
O3	-1.2631290000	-2.0977820000	0.3165840000
C4	-0.3235520000	-3.1513550000	0.4617090000
C5	0.4781750000	-3.0014760000	1.7476280000
C6	-1.0722530000	-4.4645180000	0.3912470000
O7	0.5312840000	-3.0549020000	-0.6704290000
C8	0.4631570000	-1.7742350000	-1.2377330000
C9	1.6824400000	-0.8993240000	-0.9557500000
N10	1.2618180000	-0.0202300000	0.1401900000
O11	1.9762830000	0.9856570000	0.4585890000
C12	1.1257320000	2.4431830000	-0.6641020000
C13	1.1472300000	3.5522230000	0.3378530000
C14	0.0003530000	3.3787510000	1.3077180000
O15	-1.2585610000	3.2257350000	0.6481050000
C16	-1.3590760000	2.4310200000	-0.4372460000
O17	-2.4538010000	2.2054520000	-0.8984530000
C18	-0.1122700000	1.8833300000	-0.9905640000
H19	-0.5166190000	0.5043820000	1.1254300000
H20	-1.5153910000	-0.7054620000	-1.1481690000
H21	-0.1924270000	-3.0394180000	2.6182740000
H22	1.2115050000	-3.8169260000	1.8281970000
H23	1.0182600000	-2.0450350000	1.7638740000
H24	-1.7726450000	-4.5484480000	1.2343500000
H25	-1.6358060000	-4.5081810000	-0.5509000000
H26	-0.3658330000	-5.3058210000	0.4297230000
H27	0.2887610000	-1.8760630000	-2.3194450000
H28	2.5684080000	-1.4801130000	-0.6634940000
H29	1.9449670000	-0.2578260000	-1.8150370000
H30	1.9391490000	2.4025710000	-1.3935530000
H31	2.0969560000	3.5613990000	0.8942930000
H32	1.0713330000	4.5216830000	-0.1882180000
H33	-0.1156010000	4.2483640000	1.9685270000
H34	0.1868650000	2.4950390000	1.9409350000
H35	-0.2519210000	1.3670960000	-1.9435940000

TS_PR-*meta-endo*

ifreq= -395 cm^{-1}, ΔG= -897.379899 Hartree

C1	-0.2963020000	0.1977780000	0.9470210000
C2	0.7969620000	1.0500400000	0.3705300000
O3	0.8454690000	2.3642260000	0.8521010000
C4	0.0525730000	3.1971860000	0.0206310000
C5	0.7878230000	4.5062390000	-0.1723830000
C6	-1.3421360000	3.3850220000	0.6004180000
O7	-0.0317420000	2.5247850000	-1.2305630000
C8	0.3954710000	1.1911120000	-1.1208520000
C9	-0.7240290000	0.1810930000	-1.3351220000
N10	-1.1978480000	-0.1154690000	0.0211970000
O11	-2.0461840000	-1.0499590000	0.2492350000
C12	-0.9735200000	-2.4977800000	1.0634240000
C13	-1.1241640000	-3.5918900000	0.0534820000
C14	-0.2681590000	-3.3363360000	-1.1648900000
O15	1.0535640000	-2.8883630000	-0.8628110000
C16	1.3550810000	-2.1802020000	0.2502530000
O17	2.4963370000	-1.7928980000	0.3810700000
C18	0.2909650000	-1.9139130000	1.2232930000
H19	-0.5969140000	0.2452990000	1.9947540000
H20	1.7791790000	0.5750050000	0.5260210000
H21	1.7836940000	4.3053100000	-0.5912180000
H22	0.2301050000	5.1544840000	-0.8631350000
H23	0.9000600000	5.0255120000	0.7901690000
H24	-1.8512270000	2.4169540000	0.7045580000
H25	-1.9426180000	4.0207320000	-0.0663650000
H26	-1.2821240000	3.8638330000	1.5885210000
H27	1.2348830000	1.0143830000	-1.8114370000
H28	-0.3466690000	-0.7428730000	-1.7995610000
H29	-1.5474050000	0.5765390000	-1.9457850000
H30	-1.6260280000	-2.5560100000	1.9389930000
H31	-0.8276690000	-4.5401350000	0.5388370000
H32	-2.1717400000	-3.7104220000	-0.2616840000
H33	-0.7511440000	-2.5781770000	-1.8030610000
H34	-0.1462070000	-4.2444880000	-1.7716310000
H35	0.6667790000	-1.6317990000	2.2076500000

TS_PR-*ortho-exo*

ifreq= -389 cm^{-1}, ΔG= -897.377507 Hartree

C1	0.3998850000	0.1616980000	-0.0220070000
C2	0.5661370000	-1.5950430000	-1.1048890000
C3	1.9122690000	-2.1204000000	-0.6623530000
C4	1.7661320000	-2.7857220000	0.6914600000
O5	0.7785380000	-3.8100510000	0.6819510000
C6	-0.4130330000	-3.5732240000	0.0943410000
O7	-1.2927630000	-4.3921340000	0.2087620000
C8	-0.5401490000	-2.3529340000	-0.7176460000
O9	-1.2854080000	-1.0225890000	0.9074120000
N10	-0.8866960000	0.0241410000	0.3459330000
C11	-1.7882740000	0.8206360000	-0.4931890000
C12	-0.8928280000	1.9071390000	-1.0738870000
O13	-0.9362710000	3.1143820000	-0.3594940000
C14	0.2692250000	3.3245140000	0.3642700000
C15	0.7245280000	4.7517130000	0.1498110000
C16	0.0696580000	2.9908150000	1.8363070000
O17	1.2239770000	2.4610000000	-0.2348890000
C18	0.5626950000	1.3985320000	-0.8653150000
H19	1.1691880000	-0.1180340000	0.7003200000
H20	0.5194400000	-1.0977020000	-2.0810170000
H21	2.2902290000	-2.8481000000	-1.4022590000
H22	2.6692420000	-1.3216790000	-0.5979280000
H23	1.4928940000	-2.0448410000	1.4634450000
H24	2.6996720000	-3.2695060000	1.0098250000
H25	-1.4985870000	-2.2779590000	-1.2336230000
H26	-2.2083040000	0.1556770000	-1.2687950000
H27	-2.6128050000	1.2196850000	0.1137180000
H28	-1.1350810000	2.1068430000	-2.1290410000
H29	-0.0185150000	5.4516930000	0.5573660000
H30	1.6865590000	4.9223870000	0.6538840000
H31	0.8434010000	4.9373470000	-0.9266610000
H32	-0.6838360000	3.6621470000	2.2728310000
H33	1.0155650000	3.1154310000	2.3829950000
H34	-0.2758130000	1.9559260000	1.9627000000
H35	1.0941070000	1.1790250000	-1.8052180000

TS_PR-*ortho-endo*

ifreq= -408 cm^{-1}, ΔG= -897.371939 Hartree

C1	-0.1750580000	0.2515000000	1.0159790000
C2	0.6181310000	-1.6029480000	1.2777390000
C3	1.8512990000	-1.7247590000	0.3938770000
C4	1.8703580000	-3.0796960000	-0.2811170000
O5	0.6412930000	-3.3470410000	-0.9500040000
C6	-0.4905610000	-3.3305410000	-0.1902500000
O7	-1.4269840000	-4.0127910000	-0.5206290000
C8	-0.4613200000	-2.4513840000	0.9902190000
O9	-1.8152480000	-0.9860420000	0.0850830000
N10	-0.9863050000	-0.0529630000	-0.0202220000
C11	-0.4105550000	0.3168410000	-1.3179050000
C12	0.6434450000	1.3654630000	-0.9706650000
O13	0.2321940000	2.6872440000	-1.1914620000
C14	-0.0916830000	3.3243890000	0.0363170000
C15	0.4800230000	4.7248900000	0.0159830000
C16	-1.5959780000	3.3096470000	0.2714510000
O17	0.5879240000	2.5808240000	1.0371020000
C18	0.8339250000	1.2798460000	0.5749300000
H19	-0.6350840000	0.3184170000	2.0050230000
H20	0.8061440000	-1.4012320000	2.3373040000
H21	1.8911870000	-0.9471930000	-0.3853200000
H22	2.7684190000	-1.6015290000	0.9902360000
H23	2.0559940000	-3.8773250000	0.4607790000
H24	2.6560510000	-3.1449610000	-1.0472670000
H25	-1.2312970000	-2.6617860000	1.7325620000
H26	-1.1993950000	0.7148890000	-1.9718400000
H27	0.0081580000	-0.5895860000	-1.7859630000
H28	1.5757240000	1.1935230000	-1.5309530000
H29	1.5639000000	4.6705780000	-0.1559170000
H30	0.0155990000	5.3113170000	-0.7894070000
H31	0.2912060000	5.2267150000	0.9756840000
H32	-1.9856100000	2.2825960000	0.2725490000
H33	-2.1059050000	3.8753290000	-0.5216820000
H34	-1.8287120000	3.7712360000	1.2419820000
H35	1.8548560000	1.0021230000	0.8790890000

Product_MVK-*meta-exo*

ΔG= -784,179939 Hartree

C1	-0.031900000	0.5982000000	0.2024000000
C2	-0.590500000	-0.543300000	-0.654300000
O3	-1.286100000	-1.516300000	0.0707000000
C4	-0.455400000	-2.643200000	0.3117000000
C5	-1.269900000	-3.897200000	0.0672000000
C6	0.1336000000	-2.591200000	1.7140000000
O7	0.5720000000	-2.571500000	-0.662400000
C8	0.6600000000	-1.281100000	-1.206400000
C9	1.8509000000	-0.462600000	-0.720200000
N10	1.3561000000	0.2342000000	0.4680000000
O11	2.0301000000	1.4279000000	0.7148000000
C12	1.4946000000	2.4276000000	-0.157800000
C13	0.0674000000	1.9924000000	-0.484900000
C14	-1.007000000	2.9531000000	0.0110000000
C15	-2.427600000	2.5934000000	-0.332300000
O16	-0.734500000	3.9465000000	0.6439000000
H17	-0.592900000	0.6693000000	1.1481000000
H18	-1.255200000	-0.159500000	-1.443100000
H19	-2.108000000	-3.954800000	0.7770000000
H20	-0.642300000	-4.791300000	0.1945000000
H21	-1.669600000	-3.884900000	-0.957200000
H22	-0.668600000	-2.608800000	2.4670000000
H23	0.7269000000	-1.673100000	1.8397000000
H24	0.7892000000	-3.459000000	1.8809000000
H25	0.6644000000	-1.367600000	-2.304000000
H26	2.1586000000	0.2482000000	-1.510900000
H27	2.7131000000	-1.099300000	-0.474000000
H28	2.1085000000	2.5303000000	-1.069600000
H29	1.5097000000	3.3797000000	0.3912000000
H30	-0.076200000	1.8738000000	-1.571800000
H31	-3.114000000	3.3482000000	0.0737000000
H32	-2.550300000	2.5326000000	-1.426100000
H33	-2.690600000	1.6033000000	0.0745000000

Product_MVK-*meta-endo*

ΔG= -784,177472 Hartree

C1	-0.300700000	0.5373000000	-0.843600000
C2	0.7560000000	-0.367200000	-0.199000000
O3	0.9051000000	-1.590400000	-0.867500000
C4	0.2671000000	-2.636100000	-0.146400000
C5	1.1982000000	-3.831200000	-0.110000000
C6	-1.083800000	-2.965900000	-0.765800000
O7	0.1117000000	-2.146400000	1.1723000000
C8	0.1815000000	-0.744900000	1.1922000000
C9	-1.163600000	-0.027800000	1.2814000000
N10	-1.529400000	0.2505000000	-0.110300000
O11	-2.335100000	1.3767000000	-0.249300000
C12	-1.486400000	2.5212000000	-0.163800000
C13	-0.134600000	2.0965000000	-0.751100000
C14	1.0495000000	2.5315000000	0.1006000000
C15	2.2724000000	2.9977000000	-0.636400000
O16	1.0068000000	2.4875000000	1.3114000000
H17	-0.415300000	0.2614000000	-1.901800000
H18	1.7436000000	0.1140000000	-0.145900000
H19	1.3832000000	-4.204400000	-1.127800000
H20	0.7540000000	-4.643000000	0.4845000000
H21	2.1562000000	-3.539800000	0.3442000000
H22	-1.721400000	-2.069200000	-0.778800000
H23	-1.586100000	-3.752000000	-0.182000000
H24	-0.953200000	-3.325400000	-1.797600000
H25	0.8367000000	-0.444000000	2.0228000000
H26	-1.925500000	-0.653700000	1.7685000000
H27	-1.040500000	0.9032000000	1.8610000000
H28	-1.365100000	2.8546000000	0.8814000000
H29	-1.975600000	3.3207000000	-0.737800000
H30	-0.006000000	2.4886000000	-1.769100000
H31	2.5622000000	2.2671000000	-1.409200000
H32	2.0423000000	3.9387000000	-1.164700000
H33	3.1037000000	3.1631000000	0.0616000000

Product_MVK-*ortho-exo* (**2**)

ΔG= -784,184817 Hartree

C1	-0.660600000	0.3538000000	0.4196000000
C2	-1.484100000	1.4883000000	-0.194000000
C3	-0.387700000	2.4110000000	-0.749000000
O4	0.7772000000	1.6277000000	-0.778800000
N5	0.7032000000	0.9143000000	0.4763000000
C6	1.6235000000	-0.199800000	0.3662000000
C7	0.9519000000	-1.256200000	-0.525700000
O8	1.0997000000	-2.558400000	-0.013800000
C9	-0.186700000	-3.141700000	0.1117000000
C10	-0.550500000	-3.898100000	-1.161100000
C11	-0.209200000	-4.020600000	1.3419000000
O12	-1.063400000	-2.051800000	0.3164000000
C13	-0.558400000	-0.950800000	-0.400000000
C14	-0.210700000	3.6909000000	0.0790000000
C15	1.1940000000	4.1847000000	0.2639000000
O16	-1.180200000	4.2706000000	0.5107000000
H17	-0.978000000	0.0897000000	1.4392000000
H18	-2.150300000	1.1153000000	-0.983600000
H19	-2.095800000	2.0253000000	0.5428000000
H20	-0.580900000	2.7352000000	-1.785800000
H21	2.5976000000	0.1577000000	0.0070000000
H22	1.7549000000	-0.628000000	1.3719000000
H23	1.3057000000	-1.202500000	-1.568400000
H24	0.1597000000	-4.720300000	-1.333500000
H25	-0.519400000	-3.231800000	-2.037100000
H26	-1.564500000	-4.318000000	-1.083400000
H27	-1.209200000	-4.454800000	1.4853000000
H28	0.0506000000	-3.420900000	2.2258000000
H29	0.5171000000	-4.839600000	1.2391000000
H30	-1.053700000	-0.858000000	-1.382100000
H31	1.7661000000	3.4379000000	0.8367000000
H32	1.7002000000	4.2934000000	-0.708500000
H33	1.1839000000	5.1447000000	0.7966000000

Product_MVK-*ortho-endo* (**3**)

ΔG= -784,181848 Hartree

C1	0.6763000000	0.0760000000	-0.925600000
C2	1.3626000000	1.4405000000	-0.753100000
C3	0.2016000000	2.4251000000	-0.859700000
O4	-0.995800000	1.6343000000	-0.980500000
N5	-0.716700000	0.3327000000	-0.553100000
C6	-0.791800000	0.1854000000	0.9014000000
C7	0.1369000000	-0.984700000	1.2120000000
O8	-0.482100000	-2.243600000	1.1907000000
C9	-0.130500000	-2.962700000	0.0203000000
C10	0.2361000000	-4.379700000	0.4139000000
C11	-1.261200000	-2.912100000	-0.997300000
O12	1.0313000000	-2.324100000	-0.484700000
C13	1.1427000000	-1.026600000	0.0285000000
C14	0.1088000000	3.4284000000	0.2959000000
C15	-1.213900000	4.1192000000	0.4647000000
O16	1.0694000000	3.6673000000	0.9914000000
H17	0.7308000000	-0.271600000	-1.968500000
H18	2.1310000000	1.6147000000	-1.517700000
H19	1.8456000000	1.5434000000	0.2305000000
H20	0.2517000000	3.0349000000	-1.780100000
H21	-1.823100000	-0.010400000	1.2294000000
H22	-0.426900000	1.0888000000	1.4262000000
H23	0.6188000000	-0.851900000	2.1927000000
H24	1.0571000000	-4.358700000	1.1451000000
H25	-0.629600000	-4.887800000	0.8632000000
H26	0.5584000000	-4.952000000	-0.468300000
H27	-0.967400000	-3.434100000	-1.920400000
H28	-2.159600000	-3.400400000	-0.590400000
H29	-1.505200000	-1.866200000	-1.236200000
H30	2.1877000000	-0.864100000	0.3332000000
H31	-2.005900000	3.3778000000	0.6510000000
H32	-1.489800000	4.6379000000	-0.468200000
H33	-1.161100000	4.8397000000	1.2913000000

Product_EA-*meta-exo*

ΔG= -898,622060 Hartree

C1	-0.461800000	-0.009000000	-0.276400000
C2	0.5849000000	-0.805500000	0.5051000000
O3	1.4260000000	-1.587400000	-0.293100000
C4	0.9994000000	-2.941400000	-0.290600000
C5	2.2202000000	-3.825800000	-0.135100000
C6	0.2033000000	-3.260100000	-1.548000000
O7	0.1875000000	-3.081800000	0.8633000000
C8	-0.238700000	-1.830500000	1.3336000000
C9	-1.699800000	-1.504200000	1.0337000000
N10	-1.659700000	-0.836800000	-0.268600000
O11	-2.731100000	0.0290000000	-0.474000000
C12	-2.446600000	1.2735000000	0.1795000000
C13	-0.934800000	1.3147000000	0.3914000000
C14	-0.249800000	2.5163000000	-0.213500000
O15	-0.762100000	3.3202000000	-0.950400000
O16	1.0330000000	2.5500000000	0.1565000000
C17	1.8487000000	3.6089000000	-0.361700000
C18	1.7602000000	4.8507000000	0.4941000000
H19	-0.124800000	0.1735000000	-1.308800000
H20	1.2087000000	-0.134800000	1.1141000000
H21	2.7595000000	-3.553700000	0.7838000000
H22	1.9215000000	-4.882500000	-0.074200000
H23	2.8947000000	-3.702400000	-0.995100000
H24	-0.681500000	-2.609000000	-1.608800000
H25	0.8257000000	-3.106800000	-2.442400000
H26	-0.130000000	-4.308700000	-1.529600000
H27	-0.038400000	-1.779900000	2.4149000000
H28	-2.322400000	-2.409800000	0.9960000000
H29	-2.103000000	-0.839600000	1.8216000000
H30	-2.993100000	1.3504000000	1.1351000000
H31	-2.786100000	2.0789000000	-0.487700000
H32	-0.676100000	1.2943000000	1.4613000000
H33	2.8691000000	3.1996000000	-0.367000000
H34	1.5466000000	3.8180000000	-1.398700000
H35	2.4536000000	5.6156000000	0.1105000000
H36	0.7447000000	5.2742000000	0.4762000000
H37	2.0351000000	4.6339000000	1.5381000000

Product_EA-*meta-endo*

ΔG= -898,619398 Hartree

C1	-0.807000000	0.0732000000	0.8646000000
C2	0.5316000000	0.5253000000	0.2750000000
O3	1.0753000000	1.6365000000	0.9333000000
C4	0.8875000000	2.8162000000	0.1631000000
C5	-0.286900000	3.6231000000	0.6985000000
C6	2.1835000000	3.6017000000	0.1664000000
O7	0.6357000000	2.3695000000	-1.156300000
C8	0.1975000000	1.0364000000	-1.152300000
C9	-1.310500000	0.8461000000	-1.307400000
N10	-1.818200000	0.7555000000	0.0650000000
O11	-2.974600000	-0.012100000	0.1734000000
C12	-2.586200000	-1.385200000	0.1474000000
C13	-1.190400000	-1.444800000	0.7819000000
C14	-0.222600000	-2.271500000	-0.032500000
O15	-0.152000000	-2.260500000	-1.239400000
O16	0.5640000000	-3.010800000	0.7482000000
C17	1.5500000000	-3.835700000	0.1121000000
C18	2.8302000000	-3.079800000	-0.157100000
H19	-0.871900000	0.3910000000	1.9150000000
H20	1.2823000000	-0.278900000	0.2937000000
H21	-0.444900000	4.5190000000	0.0794000000
H22	-0.089000000	3.9425000000	1.7329000000
H23	-1.202200000	3.0127000000	0.6842000000
H24	2.0885000000	4.4986000000	-0.462900000
H25	2.4379000000	3.9168000000	1.1889000000
H26	2.9963000000	2.9743000000	-0.227200000
H27	0.7384000000	0.4941000000	-1.941500000
H28	-1.502800000	-0.082800000	-1.871400000
H29	-1.771700000	1.6899000000	-1.841000000
H30	-3.344200000	-1.939700000	0.7182000000
H31	-2.562400000	-1.776500000	-0.884300000
H32	-1.219700000	-1.858700000	1.7972000000
H33	1.7175000000	-4.661100000	0.8187000000
H34	1.1256000000	-4.244500000	-0.816900000
H35	3.2323000000	-2.637500000	0.7677000000
H36	2.6745000000	-2.279400000	-0.895700000
H37	3.5878000000	-3.769400000	-0.561600000

Product_EA-*ortho-exo* (**4**)

ΔG= -898,622880 Hartree

C1	-0.636900000	-0.286600000	0.5256000000
C2	-1.774700000	0.6992000000	0.2451000000
C3	-1.110900000	1.6706000000	-0.744100000
O4	0.0091000000	0.9903000000	-1.228500000
N5	0.5474000000	0.3977000000	-0.025900000
C6	1.4734000000	-0.629200000	-0.457900000
C7	0.6370000000	-1.838800000	-0.906500000
O8	1.1503000000	-3.053100000	-0.413300000
C9	0.1117000000	-3.735400000	0.2690000000
C10	0.7023000000	-4.473700000	1.4490000000
C11	-0.635100000	-4.656400000	-0.689400000
O12	-0.739200000	-2.714900000	0.7509000000
C13	-0.715300000	-1.658200000	-0.178700000
C14	-0.752400000	3.0027000000	-0.079600000
O15	-1.422600000	3.5137000000	0.7839000000
O16	0.3307000000	3.5483000000	-0.616800000
C17	0.7684000000	4.8037000000	-0.085300000
C18	1.6651000000	4.6115000000	1.1156000000
H19	-0.476200000	-0.467800000	1.5987000000
H20	-2.637400000	0.1903000000	-0.205600000
H21	-2.117300000	1.2305000000	1.1417000000
H22	-1.748800000	1.9107000000	-1.610500000
H23	2.1411000000	-0.219400000	-1.227200000
H24	2.0786000000	-0.929200000	0.4113000000
H25	0.5210000000	-1.878400000	-2.002200000
H26	1.2116000000	-3.759100000	2.1111000000
H27	1.4294000000	-5.223300000	1.1048000000
H28	-0.087800000	-4.986600000	2.0163000000
H29	-1.466700000	-5.158100000	-0.172200000
H30	0.0446000000	-5.422600000	-1.090700000
H31	-1.048300000	-4.089400000	-1.538000000
H32	-1.576400000	-1.718400000	-0.866800000
H33	1.3089000000	5.2860000000	-0.912600000
H34	-0.111300000	5.4146000000	0.1673000000
H35	1.1152000000	4.1539000000	1.9516000000
H36	2.0505000000	5.5863000000	1.4539000000
H37	2.5236000000	3.9686000000	0.8674000000

Product_EA-*ortho-endo* (**5**)

ΔG= -898,623414 Hartree

C1	0.7480000000	-0.683800000	0.6934000000
C2	0.4118000000	0.6783000000	1.3168000000
C3	0.8018000000	1.6436000000	0.2017000000
O4	0.8981000000	0.8565000000	-0.997600000
N5	0.5269000000	-0.469900000	-0.734300000
C6	-0.900100000	-0.706200000	-0.977900000
C7	-1.248800000	-1.874900000	-0.062000000
O8	-1.086500000	-3.145800000	-0.638000000
C9	0.0792000000	-3.780600000	-0.143500000
C10	-0.260700000	-5.216500000	0.2026000000
C11	1.2156000000	-3.675100000	-1.151100000
O12	0.4045000000	-3.097000000	1.0575000000
C13	-0.191200000	-1.830900000	1.0752000000
C14	-0.221100000	2.7501000000	-0.018300000
O15	-1.401500000	2.5655000000	-0.188100000
O16	0.3508000000	3.9488000000	-0.020600000
C17	-0.506100000	5.0756000000	-0.241600000
C18	0.3378000000	6.3226000000	-0.204000000
H19	1.7965000000	-0.960900000	0.8854000000
H20	-0.665200000	0.7533000000	1.5315000000
H21	0.9643000000	0.8689000000	2.2465000000
H22	1.7885000000	2.1014000000	0.3715000000
H23	-1.083700000	-0.950600000	-2.034500000
H24	-1.499500000	0.1823000000	-0.711500000
H25	-2.284600000	-1.792100000	0.3010000000
H26	0.6135000000	-5.724800000	0.6351000000
H27	-1.082600000	-5.235100000	0.9330000000
H28	-0.572500000	-5.763500000	-0.699200000
H29	2.1311000000	-4.130000000	-0.743800000
H30	0.9470000000	-4.200700000	-2.079900000
H31	1.4116000000	-2.618400000	-1.387700000
H32	-0.624000000	-1.666300000	2.0739000000
H33	-1.288900000	5.0838000000	0.5341000000
H34	-1.012000000	4.9495000000	-1.212400000
H35	1.1130000000	6.3011000000	-0.985300000
H36	0.8320000000	6.4395000000	0.7728000000
H37	-0.296800000	7.2055000000	-0.374600000

Product_CP-*meta-exo* (**6**)

ΔG= -822,259012 Hartree

C1	0.2644000000	0.4385000000	-0.175900000
C2	0.0756000000	-0.885200000	0.5951000000
O3	1.0812000000	-1.815800000	0.2810000000
C4	0.4629000000	-3.068900000	0.0534000000
C5	0.3019000000	-3.834600000	1.3622000000
C6	1.2700000000	-3.829200000	-0.974400000
O7	-0.803200000	-2.755700000	-0.497800000
C8	-1.216000000	-1.513500000	0.0191000000
C9	-1.655400000	-0.544000000	-1.090700000
N10	-1.027000000	0.7339000000	-0.817500000
O11	-1.771500000	1.3122000000	0.2790000000
C12	-0.902300000	2.2863000000	0.8050000000
C13	-0.895900000	3.6068000000	0.0067000000
C14	0.5619000000	4.0542000000	-0.042900000
C15	1.3463000000	2.7593000000	-0.050300000
O16	2.4318000000	2.5778000000	-0.539000000
C17	0.5159000000	1.6909000000	0.6565000000
H18	1.0375000000	0.2879000000	-0.944900000
H19	0.0142000000	-0.721200000	1.6849000000
H20	-0.295200000	-3.257900000	2.0856000000
H21	1.2852000000	-4.037300000	1.8123000000
H22	-0.209300000	-4.792500000	1.1849000000
H23	1.3535000000	-3.229000000	-1.891500000
H24	0.7808000000	-4.784200000	-1.214800000
H25	2.2788000000	-4.039000000	-0.590800000
H26	-1.994800000	-1.644800000	0.7884000000
H27	-2.741200000	-0.408000000	-1.180300000
H28	-1.265800000	-0.914900000	-2.051000000
H29	-1.205600000	2.4377000000	1.8514000000
H30	-1.571500000	4.3576000000	0.4401000000
H31	-1.252700000	3.3766000000	-1.009000000
H32	0.8464000000	4.5949000000	0.8792000000
H33	0.8363000000	4.6918000000	-0.895100000
H34	1.0085000000	1.4437000000	1.6096000000

Product_CP-*meta-endo*

ΔG= -822,253638 Hartree

C1	-0.249800000	-0.211100000	0.8601000000
C2	0.8199000000	0.8106000000	0.4516000000
O3	0.6446000000	2.0755000000	1.0272000000
C4	-0.029200000	2.9325000000	0.1197000000
C5	-1.522300000	2.9800000000	0.4169000000
C6	0.6205000000	4.2996000000	0.1863000000
O7	0.2014000000	2.3799000000	-1.165800000
C8	0.6062000000	1.0382000000	-1.066200000
C9	-0.453200000	0.0226000000	-1.476900000
N10	-1.200200000	-0.261400000	-0.253500000
O11	-1.740900000	-1.571500000	-0.305300000
C12	-1.120900000	-2.409800000	0.6783000000
C13	-0.696200000	-3.738300000	0.0556000000
C14	0.6583000000	-3.467400000	-0.594600000
C15	1.2289000000	-2.264400000	0.1404000000
O16	2.3578000000	-1.849500000	0.0423000000
C17	0.1553000000	-1.677500000	1.0596000000
H18	-0.749200000	0.1392000000	1.7773000000
H19	1.8248000000	0.4446000000	0.6999000000
H20	-1.697600000	3.3440000000	1.4405000000
H21	-2.025900000	3.6603000000	-0.286500000
H22	-1.964000000	1.9779000000	0.3076000000
H23	0.4966000000	4.7350000000	1.1887000000
H24	0.1612000000	4.9773000000	-0.548100000
H25	1.6940000000	4.2087000000	-0.033200000
H26	1.5241000000	0.9057000000	-1.659200000
H27	-1.111900000	0.4172000000	-2.265100000
H28	0.0322000000	-0.891100000	-1.867300000
H29	-1.819800000	-2.543300000	1.5221000000
H30	-0.597000000	-4.485100000	0.8593000000
H31	-1.460100000	-4.107200000	-0.643800000
H32	0.5676000000	-3.193500000	-1.659400000
H33	1.3679000000	-4.307100000	-0.551700000
H34	0.4727000000	-1.866100000	2.0975000000

Product_CP-*ortho-exo*

ΔG= -822,251511 Hartree

C1	0.4629000000	0.2425000000	-0.009600000
C2	0.6302000000	1.5126000000	0.8559000000
C3	1.8430000000	2.3845000000	0.4790000000
C4	1.3467000000	3.3021000000	-0.636400000
C5	-0.113000000	3.5337000000	-0.306200000
O6	-0.784300000	4.4851000000	-0.605100000
C7	-0.617700000	2.3220000000	0.4894000000
O8	-1.387700000	1.5136000000	-0.421800000
N9	-0.964700000	0.1877000000	-0.305100000
C10	-1.601600000	-0.502100000	0.8191000000
C11	-0.596000000	-1.576200000	1.2321000000
O12	-0.779200000	-2.826900000	0.6258000000
C13	0.1517000000	-3.016100000	-0.426200000
C14	0.7089000000	-4.421800000	-0.328900000
C15	-0.492600000	-2.734400000	-1.776100000
O16	1.2036000000	-2.097200000	-0.171000000
C17	0.7740000000	-1.080100000	0.6905000000
H18	1.0461000000	0.3265000000	-0.941100000
H19	0.6469000000	1.2424000000	1.9220000000
H20	2.1517000000	2.9893000000	1.3461000000
H21	2.7104000000	1.7737000000	0.1865000000
H22	1.8912000000	4.2502000000	-0.747400000
H23	1.3720000000	2.7912000000	-1.616500000
H24	-1.236400000	2.6440000000	1.3439000000
H25	-2.566200000	-0.938100000	0.5209000000
H26	-1.779800000	0.1887000000	1.6650000000
H27	-0.607200000	-1.720200000	2.3232000000
H28	1.1597000000	-4.572500000	0.6628000000
H29	1.4774000000	-4.583600000	-1.098800000
H30	-0.091600000	-5.162000000	-0.473100000
H31	-1.323300000	-3.434000000	-1.953100000
H32	0.2458000000	-2.857100000	-2.582700000
H33	-0.881500000	-1.705400000	-1.801600000
H34	1.5369000000	-0.943600000	1.4727000000

Product_CP-*ortho-endo*

ΔG= -822,247482 Hartree

C1	0.0432000000	-0.152700000	1.1007000000
C2	0.7145000000	-1.544500000	1.1203000000
C3	1.7759000000	-1.874400000	0.0382000000
C4	1.4304000000	-3.277500000	-0.461500000
C5	-0.074900000	-3.366900000	-0.322000000
O6	-0.831500000	-4.018000000	-0.992700000
C7	-0.483100000	-2.470800000	0.8569000000
O8	-1.629200000	-1.652700000	0.6244000000
N9	-1.207700000	-0.346200000	0.3621000000
C10	-0.921400000	-0.117100000	-1.053200000
C11	0.0745000000	1.0323000000	-1.041200000
O12	-0.579100000	2.2760000000	-1.063200000
C13	-0.006800000	3.1065000000	-0.074700000
C14	-1.085500000	4.0050000000	0.4864000000
C15	1.1788000000	3.8782000000	-0.642900000
O16	0.4230000000	2.2228000000	0.9444000000
C17	0.7436000000	0.9839000000	0.3639000000
H18	-0.180100000	0.1643000000	2.1304000000
H19	1.1410000000	-1.732300000	2.1151000000
H20	1.7270000000	-1.168900000	-0.804900000
H21	2.7967000000	-1.807000000	0.4398000000
H22	1.7590000000	-3.514200000	-1.483300000
H23	1.8550000000	-4.048000000	0.2089000000
H24	-0.735800000	-3.130900000	1.7041000000
H25	-0.480500000	-1.006400000	-1.542300000
H26	-1.836800000	0.1487000000	-1.602000000
H27	0.8026000000	0.9500000000	-1.867100000
H28	-1.904500000	3.3834000000	0.8753000000
H29	-0.684200000	4.6243000000	1.3015000000
H30	-1.478900000	4.6676000000	-0.298000000
H31	1.6483000000	4.4925000000	0.1398000000
H32	0.8519000000	4.5379000000	-1.460500000
H33	1.9405000000	3.1897000000	-1.041500000
H34	1.8365000000	0.8430000000	0.3199000000

Product_CH-*meta*-*exo* (**7**)

ΔG= -861,510679 Hartree

C1	-0.152500000	0.2063000000	0.0997000000
C2	-0.796000000	-1.026900000	-0.529900000
O3	-1.211700000	-1.988000000	0.3994000000
C4	-0.296000000	-3.071800000	0.4472000000
C5	0.6184000000	-2.948200000	1.6577000000
C6	-1.083900000	-4.366200000	0.4413000000
O7	0.4488000000	-2.993000000	-0.756200000
C8	0.3574000000	-1.710900000	-1.316100000
C9	1.5812000000	-0.821800000	-1.092200000
N10	1.2763000000	-0.076200000	0.1320000000
O11	1.9076000000	1.1616000000	0.1865000000
C12	1.1543000000	2.1218000000	-0.585700000
C13	1.1850000000	3.4350000000	0.1787000000
C14	0.2197000000	3.4003000000	1.3548000000
C15	-1.219000000	3.2967000000	0.8582000000
C16	-1.423000000	2.3517000000	-0.309200000
O17	-2.496200000	2.2509000000	-0.861000000
C18	-0.249000000	1.5028000000	-0.752600000
H19	-0.536700000	0.3688000000	1.1189000000
H20	-1.658800000	-0.745400000	-1.152300000
H21	0.0276000000	-2.981200000	2.5855000000
H22	1.3414000000	-3.777600000	1.6739000000
H23	1.1686000000	-1.996100000	1.6187000000
H24	-1.716200000	-4.434300000	1.3386000000
H25	-1.725100000	-4.403600000	-0.451200000
H26	-0.402500000	-5.229400000	0.4273000000
H27	0.1468000000	-1.820000000	-2.390600000
H28	2.4989000000	-1.416400000	-0.974800000
H29	1.7145000000	-0.144100000	-1.956100000
H30	1.6290000000	2.2464000000	-1.574400000
H31	2.2203000000	3.6088000000	0.5121000000
H32	0.9262000000	4.2605000000	-0.506800000
H33	0.3341000000	4.2955000000	1.9852000000
H34	0.4731000000	2.5377000000	1.9928000000
H35	-1.913300000	2.9833000000	1.6564000000
H36	-1.592300000	4.2773000000	0.5156000000
H37	-0.437600000	1.2319000000	-1.801400000

Product_CH-*meta-endo*

ΔG= -861,506351 Hartree

C1	-0.248000000	0.0065000000	0.8357000000
C2	0.7992000000	1.0842000000	0.5315000000
O3	0.4769000000	2.3306000000	1.0951000000
C4	-0.079700000	3.1889000000	0.1133000000
C5	0.5273000000	4.5673000000	0.2825000000
C6	-1.600100000	3.2057000000	0.2084000000
O7	0.3375000000	2.6611000000	-1.133300000
C8	0.7169000000	1.3149000000	-0.998300000
C9	-0.313100000	0.3082000000	-1.507200000
N10	-1.131200000	-0.020500000	-0.341900000
O11	-1.625600000	-1.351200000	-0.469900000
C12	-1.213300000	-2.097500000	0.6698000000
C13	-1.204100000	-3.572200000	0.3184000000
C14	-0.078500000	-3.932200000	-0.638000000
C15	1.2684000000	-3.495000000	-0.063800000
C16	1.3072000000	-2.016200000	0.2372000000
O17	2.2424000000	-1.323100000	-0.098500000
C18	0.1339000000	-1.466700000	1.0442000000
H19	-0.818700000	0.3298000000	1.7208000000
H20	1.7959000000	0.7874000000	0.8760000000
H21	1.6219000000	4.4998000000	0.2033000000
H22	0.1539000000	5.2489000000	-0.495800000
H23	0.2654000000	4.9832000000	1.2665000000
H24	-1.998800000	2.1905000000	0.0592000000
H25	-2.022100000	3.8675000000	-0.563100000
H26	-1.916400000	3.5741000000	1.1961000000
H27	1.6808000000	1.1738000000	-1.508500000
H28	0.1953000000	-0.593000000	-1.895100000
H29	-0.924100000	0.7341000000	-2.317300000
H30	-1.929500000	-1.922000000	1.4962000000
H31	-1.100300000	-4.142100000	1.2575000000
H32	-2.187100000	-3.835600000	-0.103700000
H33	-0.245100000	-3.437400000	-1.608600000
H34	-0.069200000	-5.015900000	-0.832200000
H35	1.4454000000	-4.022500000	0.8924000000
H36	2.1115000000	-3.726100000	-0.730600000
H37	0.3784000000	-1.668800000	2.1012000000

Product_CH-*ortho-exo*

ΔG= -861,505112 Hartree

C1	0.1436000000	-0.204100000	-0.059800000
C2	0.3443000000	-1.385500000	-1.015700000
C3	1.7234000000	-2.040200000	-1.028500000
C4	2.0112000000	-2.887400000	0.2053000000
C5	0.9007000000	-3.918700000	0.4240000000
C6	-0.413600000	-3.203200000	0.6160000000
O7	-1.100400000	-3.324700000	1.5994000000
C8	-0.810200000	-2.277000000	-0.540100000
O9	-1.848700000	-1.401400000	-0.152100000
N10	-1.313900000	-0.102400000	0.0690000000
C11	-1.770500000	0.7970000000	-0.991000000
C12	-0.693500000	1.8620000000	-1.059600000
O13	-0.911200000	2.8657000000	-0.099400000
C14	0.3207000000	3.1805000000	0.5089000000
C15	1.0517000000	4.2647000000	-0.274300000
C16	0.0636000000	3.5603000000	1.9500000000
O17	1.0693000000	1.9744000000	0.4800000000
C18	0.6008000000	1.1624000000	-0.565600000
H19	0.6060000000	-0.406600000	0.9206000000
H20	0.1123000000	-1.038800000	-2.036600000
H21	1.7988000000	-2.680900000	-1.923900000
H22	2.4932000000	-1.259300000	-1.148600000
H23	2.0944000000	-2.250000000	1.1020000000
H24	2.9823000000	-3.395600000	0.0964000000
H25	1.0823000000	-4.553700000	1.3030000000
H26	0.8264000000	-4.570900000	-0.464900000
H27	-1.184500000	-2.927500000	-1.352300000
H28	-1.852700000	0.2705000000	-1.960200000
H29	-2.752800000	1.2289000000	-0.748400000
H30	-0.598100000	2.2904000000	-2.072300000
H31	0.4654000000	5.1958000000	-0.283100000
H32	2.0327000000	4.4699000000	0.1792000000
H33	1.2163000000	3.9502000000	-1.316800000
H34	-0.548500000	4.4723000000	2.0033000000
H35	1.0128000000	3.7427000000	2.4742000000
H36	-0.472200000	2.7397000000	2.4480000000
H37	1.3676000000	1.0616000000	-1.352900000

Product_CH-*ortho-endo*

ΔG= -861,499234 Hartree

C1	0.2986000000	0.1378000000	0.9358000000
C2	0.7768000000	-1.338900000	0.9563000000
C3	1.7555000000	-1.810400000	-0.117700000
C4	1.8496000000	-3.334500000	-0.084900000
C5	0.5155000000	-3.968800000	-0.504000000
C6	-0.676700000	-3.057500000	-0.294800000
O7	-1.655500000	-3.098000000	-1.000400000
C8	-0.567200000	-2.109500000	0.8971000000
O9	-1.603800000	-1.145700000	0.9601000000
N10	-1.104900000	0.0861000000	0.5252000000
C11	-1.146700000	0.2460000000	-0.929200000
C12	-0.038100000	1.2474000000	-1.228000000
O13	-0.437000000	2.5936000000	-1.199300000
C14	0.0386000000	3.2365000000	-0.029100000
C15	0.6196000000	4.5817000000	-0.416400000
C16	-1.073400000	3.3565000000	1.0041000000
O17	1.0929000000	2.4174000000	0.4515000000
C18	0.9591000000	1.1138000000	-0.046000000
H19	0.3719000000	0.5546000000	1.9517000000
H20	1.2472000000	-1.532600000	1.9317000000
H21	1.4244000000	-1.487700000	-1.121300000
H22	2.7460000000	-1.359200000	0.0498000000
H23	2.1282000000	-3.650700000	0.9345000000
H24	2.6535000000	-3.690600000	-0.747200000
H25	0.5050000000	-4.242100000	-1.570100000
H26	0.3178000000	-4.901600000	0.0529000000
H27	-0.683500000	-2.757200000	1.7857000000
H28	-2.130000000	0.6149000000	-1.256400000
H29	-0.948100000	-0.703300000	-1.455800000
H30	0.4203000000	1.0438000000	-2.208500000
H31	1.4170000000	4.4390000000	-1.160000000
H32	-0.161100000	5.2244000000	-0.848600000
H33	1.0395000000	5.0871000000	0.4655000000
H34	-1.490100000	2.3628000000	1.2271000000
H35	-1.881100000	3.9962000000	0.6172000000
H36	-0.686500000	3.8045000000	1.9317000000
H37	1.9560000000	0.7682000000	-0.353000000

Product_FR-*meta-exo* (**8**)

ΔG= -858,194445 Hartree

C1	0.3220000000	0.4403000000	-0.133100000
C2	0.0232000000	-0.876500000	0.6123000000
O3	1.0451000000	-1.821300000	0.4277000000
C4	0.4453000000	-3.055900000	0.0719000000
C5	1.3785000000	-3.799500000	-0.855800000
C6	0.0887000000	-3.855800000	1.3196000000
O7	-0.722400000	-2.698200000	-0.642700000
C8	-1.198400000	-1.479600000	-0.124900000
C9	-1.521500000	-0.470100000	-1.239700000
N10	-0.900100000	0.7884000000	-0.869200000
O11	-1.724600000	1.3248000000	0.1962000000
C12	-0.891200000	2.2149000000	0.8977000000
C13	-0.842400000	3.6286000000	0.2979000000
O14	0.4444000000	3.8127000000	-0.280100000
C15	1.2566000000	2.7658000000	-0.079800000
O16	2.3767000000	2.7279000000	-0.501000000
C17	0.5380000000	1.6864000000	0.7145000000
H18	1.1463000000	0.2677000000	-0.841300000
H19	-0.172100000	-0.708300000	1.6856000000
H20	1.5954000000	-3.175800000	-1.734600000
H21	2.3217000000	-4.034100000	-0.341800000
H22	0.9175000000	-4.740500000	-1.189100000
H23	-0.417300000	-4.792400000	1.0421000000
H24	0.9963000000	-4.100800000	1.8909000000
H25	-0.585900000	-3.283600000	1.9754000000
H26	-2.054700000	-1.642600000	0.5504000000
H27	-2.592400000	-0.312900000	-1.422700000
H28	-1.049600000	-0.814200000	-2.172200000
H29	-1.249400000	2.2274000000	1.9361000000
H30	-0.993800000	4.4090000000	1.0592000000
H31	-1.606300000	3.7378000000	-0.486000000
H32	1.0953000000	1.4827000000	1.6392000000

Product_FR-*meta-endo* (**9**)

ΔG= -858,189351 Hartree

C1	-0.278600000	0.2482000000	-0.946000000
C2	0.7917000000	-0.761100000	-0.519200000
O3	0.5846000000	-2.044800000	-1.038800000
C4	0.0074000000	-2.893300000	-0.057600000
C5	0.7263000000	-4.226900000	-0.097900000
C6	-1.492600000	-3.030600000	-0.277100000
O7	0.2752000000	-2.272000000	1.1880000000
C8	0.6034000000	-0.919200000	1.0137000000
C9	-0.511300000	0.0574000000	1.3868000000
N10	-1.251700000	0.2561000000	0.1399000000
O11	-1.911100000	1.4960000000	0.0971000000
C12	-1.123200000	2.4615000000	-0.610500000
C13	-0.581000000	3.5756000000	0.2795000000
O14	0.7519000000	3.2110000000	0.6312000000
C15	1.2210000000	2.2219000000	-0.147000000
O16	2.3530000000	1.8321000000	-0.073300000
C17	0.1252000000	1.7306000000	-1.082400000
H18	-0.747300000	-0.076600000	-1.887500000
H19	1.7957000000	-0.418300000	-0.802900000
H20	0.3370000000	-4.895100000	0.6841000000
H21	0.5826000000	-4.710500000	-1.075200000
H22	1.8020000000	-4.071200000	0.0684000000
H23	-1.694900000	-3.476000000	-1.262800000
H24	-1.974200000	-2.042700000	-0.223700000
H25	-1.930700000	-3.679400000	0.4963000000
H26	1.5179000000	-0.704800000	1.5865000000
H27	-0.081100000	1.0031000000	1.7647000000
H28	-1.163100000	-0.355000000	2.1708000000
H29	-1.749900000	2.8649000000	-1.419700000
H30	-0.549600000	4.5370000000	-0.257400000
H31	-1.172500000	3.6951000000	1.1979000000
H32	0.4274000000	1.9727000000	-2.111500000

Product_FR-*ortho-exo*

ΔG= -858,189104 Hartree

C1	0.4721000000	-0.330000000	0.1931000000
C2	1.0628000000	-1.493400000	-0.611500000
C3	1.9326000000	-2.488400000	0.1576000000
O4	1.0737000000	-3.525600000	0.6270000000
C5	-0.136000000	-3.483200000	0.0513000000
O6	-0.999000000	-4.275300000	0.2877000000
C7	-0.207800000	-2.298000000	-0.926200000
O8	-1.293100000	-1.469800000	-0.647100000
N9	-0.867600000	-0.812600000	0.5688000000
C10	-1.714000000	0.3547000000	0.7224000000
C11	-1.186200000	1.4338000000	-0.236900000
O12	-1.023100000	2.6744000000	0.4075000000
C13	0.2603000000	3.1749000000	0.0861000000
C14	0.2150000000	3.9864000000	-1.203800000
C15	0.7832000000	3.9698000000	1.2609000000
O16	1.0682000000	2.0217000000	-0.080300000
C17	0.2562000000	0.9891000000	-0.578600000
H18	1.0429000000	-0.104600000	1.1067000000
H19	1.5849000000	-1.125300000	-1.503900000
H20	2.4394000000	-2.042700000	1.0259000000
H21	2.6968000000	-2.941900000	-0.493800000
H22	-0.307900000	-2.664800000	-1.958200000
H23	-1.603600000	0.7206000000	1.7544000000
H24	-2.761500000	0.0677000000	0.5628000000
H25	-1.815600000	1.5348000000	-1.136700000
H26	-0.449300000	4.8553000000	-1.086000000
H27	-0.162900000	3.3783000000	-2.040300000
H28	1.2211000000	4.3450000000	-1.467600000
H29	0.7936000000	3.3344000000	2.1579000000
H30	1.8048000000	4.3235000000	1.0600000000
H31	0.1409000000	4.8423000000	1.4479000000
H32	0.4065000000	0.8560000000	-1.664000000

Product_FR-*ortho-endo*

ΔG= -858,183916 Hartree

C1	-0.442900000	-0.296500000	1.1184000000
C2	0.0984000000	-1.696400000	1.4101000000
C3	1.5821000000	-1.957300000	1.1529000000
O4	1.6812000000	-2.515900000	-0.157200000
C5	0.4929000000	-2.928000000	-0.633900000
O6	0.3765000000	-3.498200000	-1.677500000
C7	-0.615800000	-2.525000000	0.3378000000
O8	-1.507000000	-1.662800000	-0.329700000
N9	-1.784500000	-0.591700000	0.6087000000
C10	-2.176600000	0.5452000000	-0.205400000
C11	-0.896700000	1.1341000000	-0.819300000
O12	-0.806900000	2.5240000000	-0.612100000
C13	0.5010000000	2.8176000000	-0.163100000
C14	0.4432000000	4.0146000000	0.7583000000
C15	1.4456000000	3.0228000000	-1.341600000
O16	0.8885000000	1.6793000000	0.5912000000
C17	0.2567000000	0.5531000000	0.0347000000
H18	-0.526700000	0.3181000000	2.0271000000
H19	-0.189600000	-1.999600000	2.4239000000
H20	2.2042000000	-1.051300000	1.1888000000
H21	1.9948000000	-2.685000000	1.8687000000
H22	-1.130900000	-3.421100000	0.7266000000
H23	-2.622700000	1.2963000000	0.4638000000
H24	-2.934200000	0.2296000000	-0.934200000
H25	-0.790100000	0.8840000000	-1.887700000
H26	-0.253500000	3.8088000000	1.5834000000
H27	1.4388000000	4.2267000000	1.1739000000
H28	0.0963000000	4.9024000000	0.2104000000
H29	2.4684000000	3.2143000000	-0.984600000
H30	1.4697000000	2.1322000000	-1.988400000
H31	1.1181000000	3.8792000000	-1.949500000
H32	0.9648000000	-0.031300000	-0.576400000

Product_PR-*meta-exo* (**10**)

ΔG= -897,443206 Hartree

C1	0.1560000000	0.2129000000	-0.112900000
C2	0.7930000000	-1.012800000	0.5399000000
O3	1.2351000000	-1.975200000	-0.373900000
C4	0.3262000000	-3.064700000	-0.437400000
C5	-0.565600000	-2.949800000	-1.665500000
C6	1.1207000000	-4.354700000	-0.412600000
O7	-0.441900000	-2.986100000	0.7516000000
C8	-0.373400000	-1.699900000	1.3037000000
C9	-1.598600000	-0.821700000	1.0442000000
N10	-1.269300000	-0.077300000	-0.174600000
O11	-1.905200000	1.1590000000	-0.247700000
C12	-1.186000000	2.1110000000	0.5589000000
C13	-1.178000000	3.4309000000	-0.185700000
C14	-0.129900000	3.3942000000	-1.272500000
O15	1.1783000000	3.2453000000	-0.714400000
C16	1.3870000000	2.3823000000	0.2861000000
O17	2.4833000000	2.2854000000	0.7723000000
C18	0.2263000000	1.5190000000	0.7298000000
H19	0.5662000000	0.3688000000	-1.122800000
H20	1.6394000000	-0.719700000	1.1791000000
H21	-1.122100000	-2.001100000	-1.641300000
H22	-1.282800000	-3.783800000	-1.692100000
H23	0.0430000000	-2.983200000	-2.581400000
H24	1.7670000000	-4.423100000	-1.299600000
H25	0.4432000000	-5.220800000	-0.406300000
H26	1.7476000000	-4.384800000	0.4901000000
H27	-0.187800000	-1.799400000	2.3836000000
H28	-2.507900000	-1.424400000	0.9069000000
H29	-1.759700000	-0.142900000	1.9023000000
H30	-1.680800000	2.2174000000	1.5387000000
H31	-0.961300000	4.2566000000	0.5113000000
H32	-2.173300000	3.6121000000	-0.619100000
H33	-0.096800000	4.3291000000	-1.847900000
H34	-0.326300000	2.5692000000	-1.978300000
H35	0.4249000000	1.2681000000	1.7804000000

Product_PR-*meta-endo*

ΔG= -897,440969 Hartree

C1	-0.360900000	0.2677000000	-1.076900000
C2	0.1986000000	-0.681200000	0.0074000000
O3	0.8507000000	-1.780400000	-0.576600000
C4	0.4910000000	-2.934400000	0.1561000000
C5	0.5119000000	-4.125800000	-0.774400000
C6	1.3993000000	-3.114500000	1.3668000000
O7	-0.844800000	-2.698600000	0.5734000000
C8	-1.039900000	-1.310900000	0.6890000000
C9	-2.225100000	-0.816600000	-0.155500000
N10	-1.803700000	0.4024000000	-0.816800000
O11	-1.824600000	1.4345000000	0.1848000000
C12	-1.061400000	2.4671000000	-0.395200000
C13	-0.603800000	3.4090000000	0.6943000000
C14	0.4647000000	2.7453000000	1.5295000000
O15	1.6127000000	2.4119000000	0.7473000000
C16	1.4984000000	1.9171000000	-0.492500000
O17	2.4927000000	1.6353000000	-1.106300000
C18	0.1130000000	1.7361000000	-1.089000000
H19	-0.210300000	-0.220300000	-2.050200000
H20	0.8589000000	-0.174100000	0.7320000000
H21	-0.148200000	-3.933400000	-1.632200000
H22	1.5322000000	-4.308100000	-1.141600000
H23	0.1624000000	-5.025900000	-0.248500000
H24	1.3537000000	-2.235100000	2.0276000000
H25	1.0893000000	-3.995200000	1.9484000000
H26	2.4431000000	-3.251300000	1.0472000000
H27	-1.131600000	-1.016200000	1.7473000000
H28	-3.148500000	-0.628300000	0.4082000000
H29	-2.435100000	-1.567700000	-0.932200000
H30	-1.665900000	2.9910000000	-1.157400000
H31	-1.457100000	3.6943000000	1.3286000000
H32	-0.207000000	4.3309000000	0.2389000000
H33	0.0728000000	1.8313000000	2.0066000000
H34	0.8360000000	3.4074000000	2.3236000000
H35	0.2125000000	2.0688000000	-2.130500000

Product_PR-*ortho*-*exo*

ΔG= -897,435323 Hartree

C1	0.4769000000	0.0368000000	-0.133600000
C2	0.5105000000	-1.301700000	-0.919500000
C3	1.8011000000	-2.085400000	-0.685200000
C4	1.7299000000	-2.819200000	0.6364000000
O5	0.6982000000	-3.808600000	0.6007000000
C6	-0.485900000	-3.499800000	0.0692000000
O7	-1.336200000	-4.338700000	-0.054000000
C8	-0.706600000	-2.049600000	-0.342200000
O9	-1.040000000	-1.342400000	0.8626000000
N10	-0.765100000	0.0084000000	0.6364000000
C11	-1.802300000	0.6642000000	-0.164100000
C12	-1.072900000	1.7684000000	-0.922600000
O13	-1.048800000	3.0120000000	-0.273400000
C14	0.2304000000	3.2638000000	0.2834000000
C15	0.2338000000	2.9653000000	1.7763000000
C16	0.6208000000	4.6945000000	-0.025300000
O17	1.1187000000	2.3990000000	-0.408600000
C18	0.4145000000	1.3244000000	-0.963800000
H19	1.3408000000	0.0901000000	0.5481000000
H20	0.3624000000	-1.123400000	-1.994600000
H21	1.9588000000	-2.825600000	-1.486800000
H22	2.6731000000	-1.412700000	-0.693100000
H23	1.5429000000	-2.131500000	1.4782000000
H24	2.6559000000	-3.371200000	0.8479000000
H25	-1.560600000	-2.057300000	-1.037600000
H26	-2.262000000	-0.036200000	-0.885400000
H27	-2.599500000	1.0681000000	0.4767000000
H28	-1.511600000	1.9101000000	-1.922300000
H29	1.2409000000	3.1155000000	2.1934000000
H30	-0.079300000	1.9263000000	1.9591000000
H31	-0.463800000	3.6377000000	2.2978000000
H32	-0.080800000	5.3941000000	0.4518000000
H33	1.6331000000	4.9050000000	0.3496000000
H34	0.6021000000	4.8557000000	-1.112800000
H35	0.7948000000	1.1561000000	-1.982800000

Product_PR-*ortho-endo*

ΔG= -897,433743 Hartree

C1	-0.280200000	0.0615000000	-1.183700000
C2	0.1803000000	1.5070000000	-1.319300000
C3	1.6367000000	1.8547000000	-1.045900000
C4	1.7098000000	3.2647000000	-0.494800000
O5	1.0460000000	3.3436000000	0.7679000000
C6	-0.124600000	2.7119000000	0.9442000000
O7	-0.606000000	2.6207000000	2.0379000000
C8	-0.788200000	2.2044000000	-0.328800000
O9	-1.775300000	1.2671000000	0.0027000000
N10	-1.724600000	0.2294000000	-1.013500000
C11	-2.201600000	-0.970400000	-0.347100000
C12	-1.064900000	-1.474200000	0.5546000000
O13	-0.780200000	-2.836300000	0.3338000000
C14	0.6237000000	-2.980100000	0.2710000000
C15	0.9552000000	-4.134100000	-0.647500000
C16	1.2173000000	-3.145600000	1.6655000000
O17	1.0854000000	-1.775500000	-0.322500000
C18	0.2009000000	-0.745200000	0.0412000000
H19	-0.087000000	-0.534600000	-2.088800000
H20	-0.090000000	1.8448000000	-2.330100000
H21	2.2417000000	1.7805000000	-1.962000000
H22	2.0837000000	1.1725000000	-0.304900000
H23	1.2642000000	3.9944000000	-1.194400000
H24	2.7451000000	3.5769000000	-0.300800000
H25	-1.233900000	3.0968000000	-0.812700000
H26	-3.140000000	-0.748900000	0.1774000000
H27	-2.399200000	-1.726600000	-1.121700000
H28	-1.255400000	-1.275500000	1.6220000000
H29	0.4934000000	-3.964600000	-1.630600000
H30	0.5732000000	-5.077100000	-0.230700000
H31	2.0439000000	-4.222300000	-0.772900000
H32	2.3132000000	-3.223300000	1.6085000000
H33	0.8237000000	-4.054600000	2.1441000000
H34	0.9683000000	-2.285300000	2.3059000000
H35	0.6437000000	-0.104800000	0.8222000000

Methyl vinyl ketone (MVK)

ΔG= -230,994493 Hartree

C1	-0.715000000	1.7258000000	-0.009400000
C2	0.0920000000	0.4560000000	0.0036000000
O3	1.3053000000	0.4600000000	0.0043000000
C4	-0.688500000	-0.819200000	0.0145000000
C5	-0.075500000	-2.006200000	-0.011200000
H6	-1.361500000	1.7721000000	0.8830000000
H7	-0.051200000	2.5999000000	-0.031500000
H8	-1.383900000	1.7425000000	-0.886000000
H9	-1.781400000	-0.746400000	0.0401000000
H10	-0.634400000	-2.946800000	-0.006400000
H11	1.0189000000	-2.052600000	-0.037700000

Ethyl acrylate (EA)

ΔG= -345,436608 Hartree

C1	-0.138200000	-2.567600000	0.7595000000
C2	0.2079000000	-1.857600000	-0.529400000
O3	0.7664000000	-0.563800000	-0.286700000
C4	-0.085100000	0.4569000000	-0.137700000
O5	-1.286400000	0.3517000000	-0.210800000
C6	0.6492000000	1.7210000000	0.1201000000
C7	-0.006800000	2.8706000000	0.2918000000
H8	-0.931400000	-2.034600000	1.3050000000
H9	-0.501200000	-3.583800000	0.5391000000
H10	0.7433000000	-2.653500000	1.4137000000
H11	-0.678500000	-1.751100000	-1.172800000
H12	0.9856000000	-2.400900000	-1.085600000
H13	1.7405000000	1.6590000000	0.1618000000
H14	-1.100600000	2.8947000000	0.2439000000
H15	0.5236000000	3.8082000000	0.4819000000

Cyclopentanone (CP)

ΔG= -269,078690 Hartree

O1	-0.015100000	2.0258000000	0.0000000000
C2	0.0076000000	0.8159000000	0.0000000000
C3	-1.212600000	-0.102200000	0.0000000000
C4	-0.656400000	-1.527500000	0.0000000000
C5	0.8327000000	-1.343000000	0.0000000000
C6	1.2049000000	-0.052700000	0.0000000000
H7	-1.830500000	0.1259000000	0.8827000000
H8	-1.830500000	0.1259000000	-0.882700000
H9	-0.972500000	-2.113900000	0.8800000000
H10	-0.972500000	-2.113900000	-0.880000000
H11	1.5218000000	-2.193400000	0.0000000000
H12	2.2237000000	0.3401000000	0.0000000000

Cyclohexanone (CH)

ΔG= -308,331569 Hartree

O1	-0.001300000	-2.281400000	0.0739000000
C2	0.0095000000	-1.069700000	-0.022100000
C3	-1.250000000	-0.267500000	-0.277900000
C4	-1.188800000	1.1278000000	0.3327000000
C5	0.0543000000	1.8818000000	-0.126100000
C6	1.2893000000	1.0449000000	-0.023100000
C7	1.2680000000	-0.298300000	0.0497000000
H8	-2.108500000	-0.850900000	0.0866000000
H9	-1.357900000	-0.196100000	-1.377000000
H10	-1.163600000	1.0371000000	1.4326000000
H11	-2.098500000	1.6968000000	0.0853000000
H12	-0.054900000	2.2083000000	-1.179200000
H13	0.1882000000	2.8116000000	0.4513000000
H14	2.2549000000	1.5653000000	-0.017100000
H15	2.1882000000	-0.884200000	0.1388000000

2(5*H*)-Furanone (FR)

ΔG= -305,010461 Hartree

O1	0.0244000000	-1.968900000	0.0000000000
C2	0.0568000000	-0.770500000	0.0000000000
O3	-1.070600000	-0.009500000	0.0000000000
C4	-0.736600000	1.3627000000	0.0000000000
C5	0.7535000000	1.4039000000	0.0000000000
C6	1.2162000000	0.1501000000	0.0000000000
H7	-1.171100000	1.8508000000	-0.890800000
H8	-1.171100000	1.8508000000	0.8908000000
H9	1.3299000000	2.3314000000	0.0000000000
H10	2.2472000000	-0.202100000	0.0000000000

5,6-Dihydro-2*H*-pyran-2-one (PR)

ΔG= -344,259190 Hartree

O1	0.0532000000	-2.218200000	0.0982000000
C2	0.0413000000	-1.018600000	-0.008700000
O3	-1.123800000	-0.352000000	0.1002000000
C4	-1.175600000	1.0156000000	-0.298600000
C5	-0.033600000	1.8253000000	0.2769000000
C6	1.2547000000	1.1215000000	9.0E-4000000
C7	1.2662000000	-0.206100000	-0.187000000
H8	-2.153900000	1.3881000000	0.0348000000
H9	-1.150200000	1.0630000000	-1.402000000
H10	-0.155000000	1.9637000000	1.3677000000
H11	-0.038500000	2.8350000000	-0.163300000
H12	2.1876000000	1.6952000000	-0.027900000
H13	2.1817000000	-0.770300000	-0.378200000

Nitrone (**1**)

ΔG= -553,177172 Hartree

C1	-0.053400000	3.0997000000	-0.128700000
C2	-0.261200000	1.5999000000	-0.154600000
C3	-1.557500000	1.2098000000	-0.854100000
O4	0.8535000000	0.9973000000	-0.802200000
C5	1.2928000000	-0.128500000	-0.089800000
C6	0.9746000000	-1.449900000	-0.773700000
N7	-0.166000000	-2.019900000	-0.013100000
O8	-0.706700000	-3.058500000	-0.427400000
C9	-0.443300000	-1.315600000	1.0543000000
C10	0.4340000000	-0.119900000	1.2038000000
O11	-0.223800000	1.1241000000	1.1771000000
H12	-0.883500000	3.5933000000	0.3976000000
H13	-0.003900000	3.4960000000	-1.153300000
H14	0.8872000000	3.3319000000	0.3910000000
H15	-1.563500000	1.5968000000	-1.884300000
H16	-2.420000000	1.6327000000	-0.317500000
H17	-1.680500000	0.1177000000	-0.896300000
H18	2.3701000000	-0.025500000	0.1109000000
H19	0.6596000000	-1.332000000	-1.819400000
H20	1.7912000000	-2.184100000	-0.722800000
H21	-1.251200000	-1.633000000	1.7141000000
H22	1.0272000000	-0.152700000	2.1325000000

Procedure for the single crystal preparation

Single crystals suitable for X-ray diffraction measurements were obtained by slowly evaporating saturated solutions of the compounds **2**, **3**, **4**, **5**, **6** and **7** in DCM. 10 mg of the products **2**, **3**, **4**, **5**, **6** and **7** were dissolved in 1 mL of DCM in a clean and dry 10 mL glass vial. The mouth of the glass vial was covered with a cap having a small hole and kept it for slow evaporation at room temperature. At the end of the process, prismatic single crystals of **2**, **3**, **4**, **5**, **6** and **7** were obtained after around 7 days.

X-Ray Crystallography

Suitable single crystals of compounds **2**, **3**, **4**, **5**, **6** and **7** were mounted on glass fibre for data collection on a Bruker Kappa APEX II diffractometer. Data were collected at 298(2) K using Cu K_α radiation (λ = 1.54178 Å) and ω scan technique, and were corrected for Lorentz and polarization effects. The detector was placed at a distance of approximately 37.5 mm from the crystal.

A series of narrow frames of data were collected with a scan width of 0.5° in ω and an exposure time of 10 s per frame. The data were integrated with SAINT[1] to a resolution of 0.78 Å using a narrow-frame algorithm. Data were corrected for absorption effects using the multi-scan method using SADABS.[2]

Subsequent structure solution and refinement were carried out with SHELXT and SHELXL, respectively.[3,4]The structures were solved by direct methods combined with difference Fourier synthesis and refined by full-matrix least-squares procedures, with anisotropic thermal parameters in the last cycles of refinement for all non-hydrogen atoms. The refinement was based on F^2 for all reflections, weighted R factors (*wR*) and goodness-of-fit (GoF) values are based on F^2, while conventional R factors (R) are based on F. The $Fo^2 > 2\sigma(Fo^2)$ criterion was used only for calculating R factors and it is not relevant to the choice of reflections for the refinement. The R factors based on F^2 are about twice as large as those based on F. Scattering factors were taken from the International Tables for Crystallography.[5] H atoms of SP^3 hybridized carbons were located directly in a difference Fourier map and freely refined. The rest of the hydrogen atoms were positioned geometrically. Mercury 4.2.0 program was used for analysis and molecular and crystal structure drawings preparation.[6]

a. Crystal Data

Table S1. Crystal data and structure refinement for compound **2**. **CCDC 2432659.**

Empirical formula	$C_{11}H_{17}NO_4$
Formula weight	227.26
Temperature (K)	298(2)
Wavelength (Å)	1.54178
Crystal system	Monoclinic
Space group	$P2_1$
Unit cell dimensions:	
a (Å)	6.1357(3)
b (Å)	5.6714(2)
c (Å)	16.5496(7)
α [º], β[º], γ[º]	90.00, 99.345(2), 90.00
Volume	568.25(4)
Z, Density (calculated) (Mg/m^3)	2, 1.328
Absorption coefficient (mm^{-1})	0.841
F(000)	244
Crystal size (mm)	0.25 x 0.22 x 0.18
2Θ range for data collection (°)	5.42 to 66.36
Limiting indices	$-7\leq h \leq7$, $-5\leq k \leq6$, $-18\leq l \leq19$
Reflections collected/Independent	4113/1740 (Rint = 0.0280)
Refinement method	Full-matrix least-squares on F^2
Data / restraints / parameters	1740 / 1 / 165
Goodness-of-fit on F^2	1.079
Final R indices [$I>2\sigma(I)$]	R1 = 0.0268, wR2 = 0.0644
R indices (all data)	R1 = 0.0276, wR2 = 0.0652
Largest diff. peak and hole ($eÅ^{-3}$)	0.140 and -0.140

Table S2. Crystal data and structure refinement for compound **3**. **CCDC 2432660.**

Empirical formula	$C_{11}H_{17}NO_4$
Formula weight	227.26
Temperature (K)	298(2)
Wavelength(Å)	1.54178
Crystal system	Monoclinic
Space group	$P2_1$
Unit celldimensions:	
a (Å)	10.175(2)
b (Å)	5.9743(13)
c (Å)	20.099(5)
α [º], β[º], γ[º]	90.00, 103.466(16), 90.00
Volume	1188.2(5)
Z, Density (calculated) (Mg/m^3)	4, 1.270
Absorption coefficient (mm^{-1})	0.804
F(000)	488
Crystal size (mm)	0.12 x 0.10 x 0.08
2Θrange for data collection (°)	4.47 to 66.89
Limiting indices	$-11\leq h \leq 10$, $-6\leq k \leq 6$, $-19\leq l \leq 23$
Reflections collected/Independent	5112 /2911(Rint = 0.0316)
Refinement method	Full-matrix least-squares on F^2
Data / restraints / parameters	2911 / 1 / 327
Goodness-of-fit on F^2	1.058
Final R indices [I>2σ(I)]	R1 = 0.0386, wR2 = 0.0882
R indices (all data)	R1 = 0.0494, wR2 = 0.0959
Largest diff. peak and hole ($eÅ^{-3}$)	0.131 and -0.151

Table S3. Crystal data and structure refinement for compound **5**. **CCDC 2432661.**

Empirical formula	$C_{12}H_{19}NO_5$
Formula weight	257.28
Temperature (K)	298(2)
Wavelength (Å)	1.54178
Crystal system	Orthorhombic
Space group	$P2_12_12_1$
Unit cell dimensions:	
a (Å)	8.8214(11)
b (Å)	26.372(3)
c (Å)	5.8630(6)
α [º], β[º], γ[º]	90.00, 90.00, 90.00
Volume	1363.9(3)
Z, Density (calculated) (Mg/m3)	4, 1.253
Absorption coefficient (mm-1)	0.817
F(000)	552
Crystal size (mm)	0.18 x 0.16 x 0.12
2Θ range for data collection (°)	3.35 to 66.46
Limiting indices	-10≤ h ≤10, -30≤ k ≤29, -4≤ l ≤6
Reflections collected/Independent	5962 / 2214 (Rint = 0.0761)
Refinement method	Full-matrixl east-squares on F^2
Data / restraints / parameters	2214 / 0 / 183
Goodness-of-fit on F2	1.034
Final R indices[I>2σ(I)]	R1 = 0.0528, wR2 = 0.1274
R indices (all data)	R1 = 0.0831, wR2 = 0.1453
Largest diff. peak and hole (eÅ-3)	0.166 and -0.153

Table S5. Crystal data and structure refinement for compound **6**. **CCDC 2432663.**

Empirical formula	$C_{12}H_{17}NO_4$
Formula weight	239.27
Temperature (K)	298(2)
Wavelength (Å)	1.54178
Crystal system	Monoclinic
Space group	$P2_1$
Unit cell dimensions:	
a (Å)	5.9342(2)
b (Å)	9.5808(3)
c (Å)	10.3586(4)
α [º], β[º], γ[º]	90.00, 94.280(2), 90.00
Volume	587.29(4)
Z, Density (calculated) (Mg/m^3)	2, 1.353
Absorption coefficient (mm^{-1})	0.844
F(000)	256
Crystal size (mm)	0.14 x 0.12 x 0.10
2Θ range for data collection (°)	4.28 to 66.33
Limiting indices	$-6\leq h \leq 6$, $-11\leq k \leq 6$, $-11\leq l \leq 11$
Reflections collected/Independent	4220 / 1203 (Rint = 0.0348)
Refinement method	Full-matrix least-squares on F^2
Data / restraints / parameters	1203 / 1 / 156
Goodness-of-fit on F^2	1.067
Final R indices [$I>2\sigma(I)$]	R1 = 0.0358, wR2 = 0.0981
R indices (all data)	R1 = 0.0363, wR2 = 0.0989
Largest diff. peak and hole ($eÅ^{-3}$)	0.237 and -0.196

Table S4. Crystal data and structure refinement for compound **7**. **CCDC 2432662.**

Empirical formula	$C_{13}H_{19}NO_4$
Formula weight	253.29
Temperature (K)	298(2)
Wavelength(Å)	1.54178
Crystal system	Triclinic
Space group	P1
Unit celldimensions:	
a (Å)	5.7707(2)
b (Å)	6.5220(3)
c (Å)	9.0485(4)
α [º], β[º], γ[º]	79.014(3), 83.589(3), 88.810(2)
Volume	332.22(2)
Z, Density (calculated) (Mg/m^3)	1, 1.266
Absorption coefficient (mm^{-1})	0.774
F(000)	136
Crystal size (mm)	0.14 x 0.12 x 0.10
2Θ range for data collection (°)	5.01 to 66.19
Limiting indices	$-6\leq h \leq 6$, $-7\leq k \leq 7$, $-7\leq l \leq 10$
Reflections collected/Independent	1463 /1008 (Rint = 0.0125)
Refinement method	Full-matrix least-squares on F^2
Data / restraints / parameters	1008 / 3 / 186
Goodness-of-fit on F^2	1.089
Final R indices [I>2σ(I)]	R1 = 0.0292, wR2 = 0.0782
R indices (all data)	R1 = 0.0294, wR2 = 0.0784
Largest diff. peak and hole ($eÅ^{-3}$)	0.116 and -0.116

b. Molecular structures of the title compounds

Compound 2: $C_{11}H_{17}NO_4$

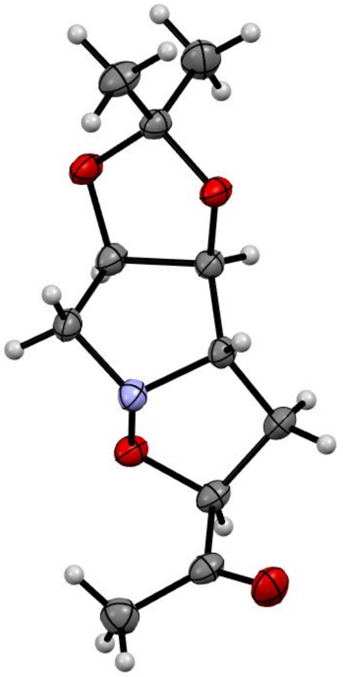

Figure S1. Molecular structure of compound **2.** Displacement ellipsoids are drawn at 50% probability level. Hydrogen atoms are shown as spheres of arbitrary radius.

Compound 3: $C_{11}H_{17}NO_4$

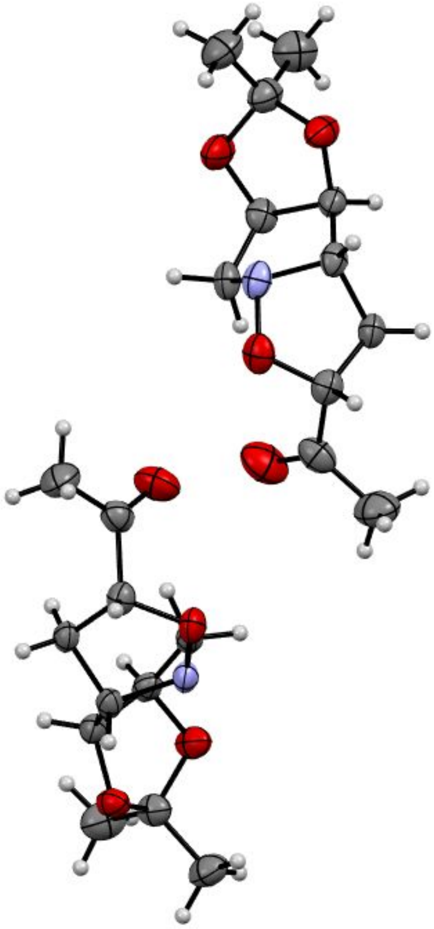

Figure S2. Molecular structure of compound **3**. Displacement ellipsoids are drawn at 50% probability level. Hydrogen atoms are shown as spheres of arbitrary radius.

Compound 5: $C_{12}H_{18}NO_5$

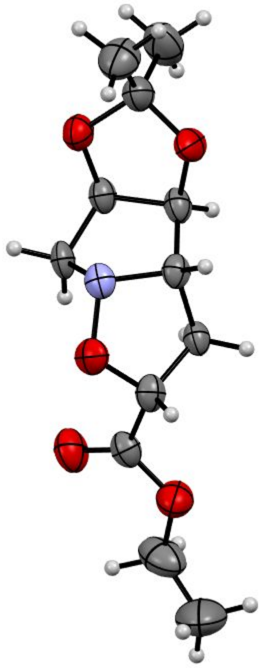

Figure S3. Molecular structure of compound **5**. Displacement ellipsoids are drawn at 50% probability level. Hydrogen atoms are shown as spheres of arbitrary radius.

Compound 6: $C_{12}H_{17}NO_4$

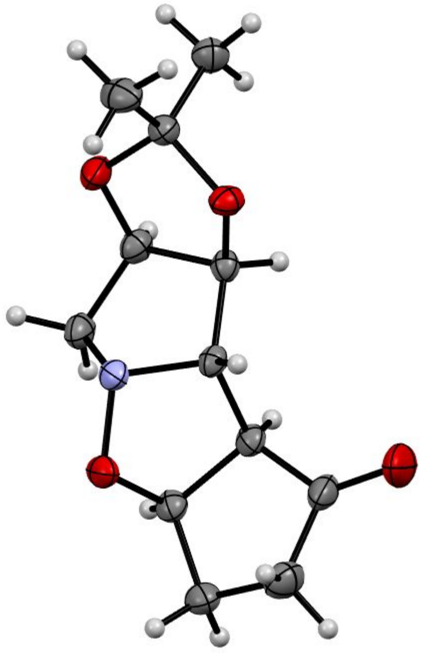

Figure S5. Molecular structure of compound **6**. Displacement ellipsoids are drawn at 50% probability level. Hydrogen atoms are shown as spheres of arbitrary radius.

Compound 7: $C_{13}H_{19}NO_4$

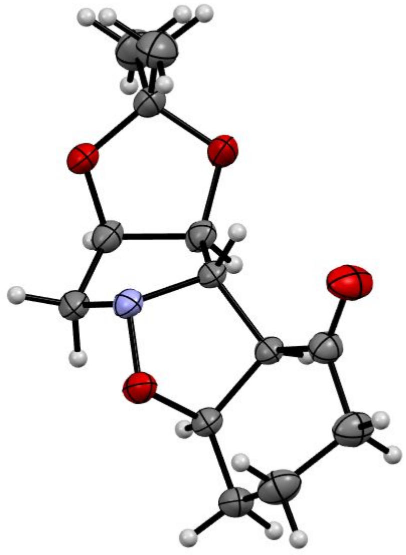

Figure S4. Molecular structure of compound **7**. Displacement ellipsoids are drawn at 50% probability level. Hydrogen atoms are shown as spheres of arbitrary radius.

References

1. SAINT-NT Version 6.0, Madison, Wisconsin, USA: Bruker –AXS, **2001**.
2. SADABS 2008/1, L. Krause, R. Herbst-Irmer, G. M. Sheldrick, D. Stalke. *J ApplCrystallogr*, **2015,** 48:3.
3. SHELXTL 2014/4, G. M. Sheldrick,*Acta Cryst. A,* **2015**, *71*, 3-8.
4. SHELXTL 2014/7, G. M. Sheldrick, *ActaCryst. A,***2015**, *71*, 3-8.
5. U. Schmueli, ed. International Tables for Crystallography. New York: Springer; **2006**.
6. C. F. Macrae, P. R. Edgington, P. McCabe, E. Pidcock, G. P. Shields, R. Taylor, M. Towler, J. J. Van de Streek, *J. Appl.Cryst,* **2006**, *39*, 453-457.
